# 3D Mass Spectrometry Imaging as a Novel Screening Method for Evaluating Biocontrol Agents

**DOI:** 10.1021/acs.jafc.5c00349

**Published:** 2025-03-31

**Authors:** Justyna Szulc, Tomasz Grzyb, Beata Gutarowska, Joanna Nizioł, Sumi Krupa, Tomasz Ruman

**Affiliations:** † Department of Environmental Biotechnology, Faculty of Biotechnology and Food Sciences, Lodz University of Technology, Wólczańska Street 171/173, 90-530 Łódź, Poland; ‡ Department of Inorganic and Analytical Chemistry, Faculty of Chemistry, Rzeszów University of Technology, Powstańców Warszawy Street 6, 35-959 Rzeszów, Poland

**Keywords:** antagonism, phytopathogens, biocontrol agents, metabolomic analysis, mass spectrometry imaging, 2D/3D biological sample imaging

## Abstract

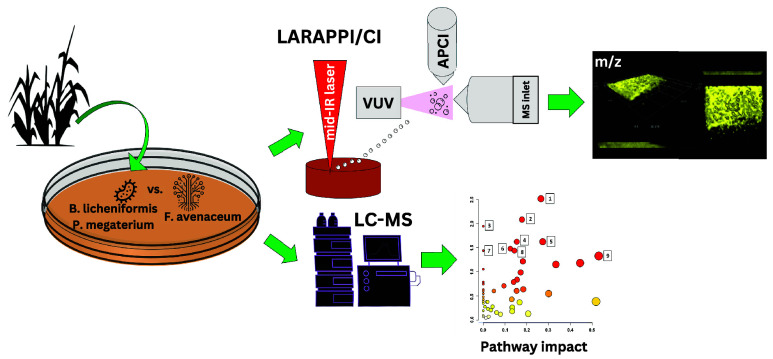

The aim of this study was to evaluate innovative mass spectrometry imaging (MSI) for determining the metabolic potential of selected soil bacteria from the genera *Bacillus* and *Priestia* in the presence of the phytopathogen *Fusarium*. This research marks the first application of direct 3D MSI that to visualize interactions between potential biocontrol agents and plant pathogens on agar medium. The LARAPPI/CI-3D-MSI (Laser-Assisted Remote Atmospheric Pressure Imaging/Chemical Ionization-3D Mass Spectrometry Imaging) setup provided valuable insights into the compounds produced by the tested microorganisms, revealing their spatial distributions and their ability to diffuse into the agar medium. Subsequently, a Pathway Impact Analysis of Metabolites was conducted. Ion images based on ultrahigh resolution mass spectrometry data were obtained, including for potentially bioactive compounds. Statistical analysis of the entire MS data set showed that the metabolites identified for *Bacillus licheniformis* samples were distinctly separated from the *Priestia megaterium* samples and could be helpful tools for screening biocontrol strains. The LARAPPI/CI MSI technique offers significant advantages over existing MSI methods. Further research using this technology could help validate the effectiveness of various biopreparations and contribute to enhancing the quality of biological plant protection products available on the market.

## 1 Introduction

The use of natural preparations to enhance agricultural productivity is a key component of the European Union’s strategic policy for promoting ecological farming.^1^ Environmental and health risks associated with the use of chemical pesticides and growing public concerns over their impact have prompted increasingly stringent legislation, which has reduced the availability of effective pesticides. At the same time, phytopathogens are gaining resistance to the active substances used to control them.^2,3^ One solution is to develop and implement new products based on biocontrol agents.^1,4−6^ Biopesticides exploit living or nonliving organisms (including microorganisms) from the natural environment to control plant diseases and pests.^7−9^ Biocontrol may be achieved through direct action—i.e. the antagonistic effects of the biocontrol organism on the pathogen (parasitism, antibiosis), as well as competition for nutrients/infection sites; it may also be achieved through indirect action, as the biocontrol factor induces plant-mediated responses that enable the plant to react more quickly and efficiently to pathogen attacks.^10,11^ In addition to their protective functions, metabolites produced by biocontrol agents can promote host plant growth, stimulate root development, and/or trigger the induction of systemic acquired resistance.^12^ Effective biocontrol agents include bacteria belonging to the genera *Bacillus*, *Paenibacillus*, *Burkholderia*, *Pseudomonas*, and *Streptomyces*, as well as fungi, viruses, yeasts, and protozoans.^13−16^

Currently, the development of new products based on biocontrol agents is a difficult and time-consuming task, mainly due to the complicated and laborious process of selecting strains with the desired phenotypic properties. The classical plate method, commonly used to evaluate the antagonistic effect between a candidate biocontrol agent and a plant pathogen, or occasionally to detect the production of a specific antimicrobial compound as a marker, has limitations.^7^ Depending on the biocontrol agent’s mechanism of action, various chemical compounds can serve as markers, including enzymes that degrade cell walls (e.g., chitinases and glucanases), enzymes that reduce pathogen virulence (e.g., pectinases and cutinases), antibiotics, siderophores, and other bioactive compounds.^17−20^ However, the classical plate method is restricted in its ability to assess the full profile of primary and secondary metabolites produced by potential biocontrol agents, as it focuses on selected single marker compounds. This highlights the need for more efficient and comprehensive methods to rapidly assess the application potential of environmental bacterial strains for use as biocontrol agents.^7^

The aim of this study was to evaluate the latest innovative mass spectrometry imaging methods for determining the metabolic potential of selected soil bacteria from the genera *Bacillus* and *Priestia* in the presence of the *Fusarium* phytophatogen. The LARAPPI/CI MSI method recently developed by Ruman et al.^21^ was applied for the first time to perform direct 2D and 3D mass spectrometry imaging (MSI) of agar medium containing cocultures of potential biocontrol agents and plant pathogens. This study also presents the first example of a simple and direct 3D MSI approach for studying microbial cultures.

## 2 Experimental Procedures

### 2.1 Biocontrol Agents and Plant Pathogens

Two strains of spore-forming soil bacteria were isolated from maize fields in central Poland after harvesting in 2023. These strains have molecularly confirmed phylogenetic affiliation and are described in the literature as potential biocontrol agents: *Bacillus licheniformis* (nucleotide sequence accession number in Gen Banck NCBI database: JBEVAF000000000) and *Priestia megaterium* (JBEVAE000000000). The strains have been deposited in the collection of pure cultures of the Department of Environmental Biotechnology, Lodz University of Technology (Poland). Additionally, *Fusarium avenaceum* IOR 1571, one of the most common fungal diseases of maize, was obtained from the Institute of Plant Protection—National Research Institute in Poznan (Poland).

### 2.2 Preparation of Cocultures

The tested microorganisms were cultured in pairs (plant pathogen–biocontrol factor), pointwise at a distance of 5 cm from each other, on TSA agar plates (Tryptic Soy Agar, Merck-Millipore, Poland). Then, the microorganisms were incubated at temp. Thirty ±2 °C for 3 days. The samples were prepared in two independent replicates of two cocultures of the same microorganisms set in a Petri dish. Areas of the agar medium containing microorganisms measuring 20–30 × 20–30 mm in size (Table 1) were cut out using stainless steel blades and transferred to stainless steel plates (45 × 35 × 0.8 mm). The plates were placed on an MSI sample table for further analysis.

**Table 1:**
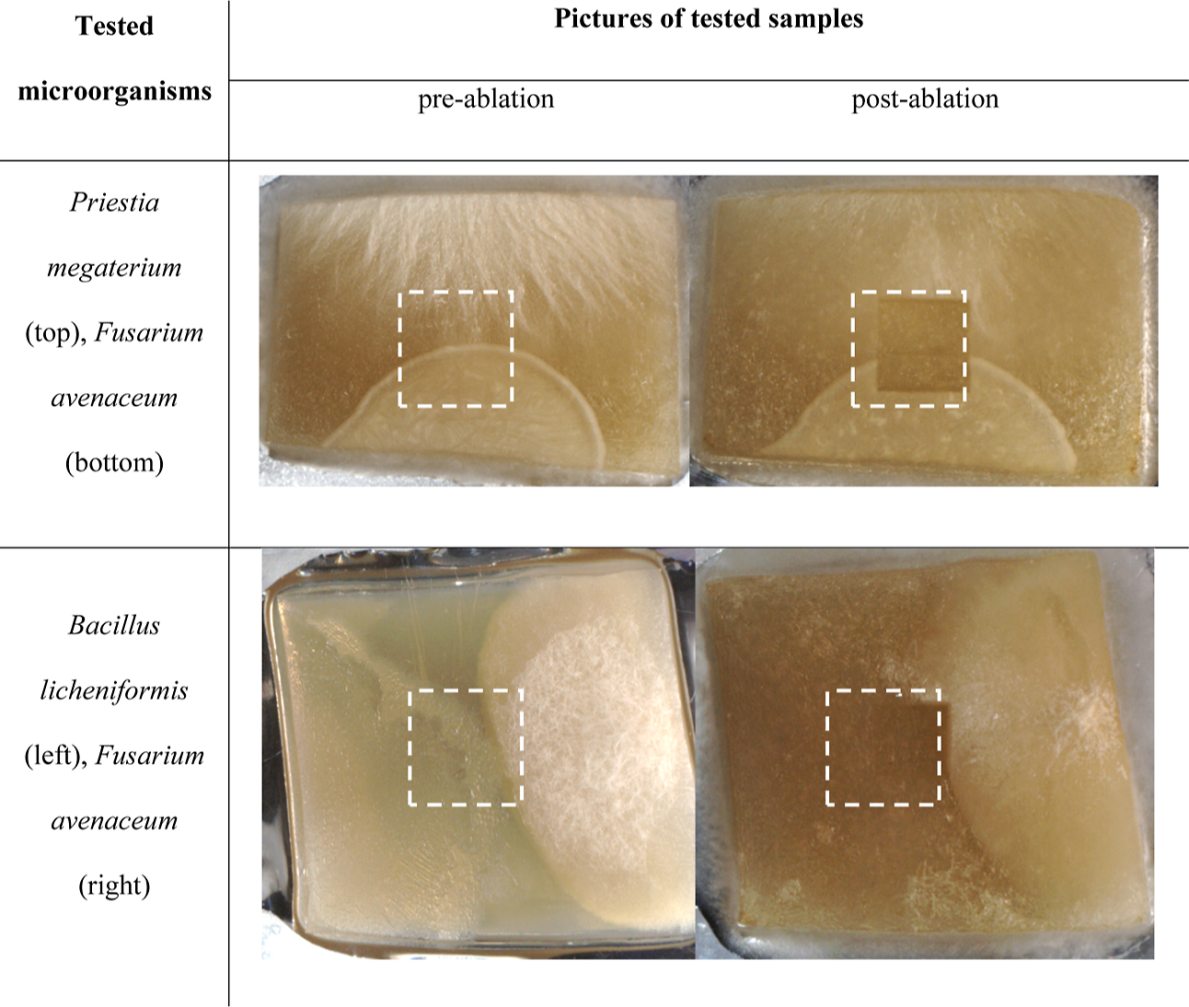
Agar Medium Samples Used in MSI Experiments

### 2.3 Mass Spectrometry Imagining (MSI) Experiments

#### 2.3.1 LARAPPI/CI MSI System

The laser ablation remote atmospheric pressure photoionization/chemical ionization (LARAPPI/CI) platform coupled to an ultrahigh resolution quadrupole-time-of-flight (QToF) mass spectrometer (LARAPPI/CI MSI system) was first described in a recent publication by Ruman et al.^21^ The setup is based on an airtight chamber pressurized with nitrogen gas to produce a nitrogen stream of 10 L/min. The sample is placed on a 50 × 50 mm sample stage, with a Peltier cooling plate that maintains the sample at −18 °C. The temperature-controlled sample stage is mounted on a motorized high-speed XY stage. The pulsed beam from the OPO laser (2.93 μm, 7 ns, 20 Hz, 3.5 mJ/pulse) enters the sample chamber through a sapphire window. The beam is expanded 3.75× and is redirected toward the sample stage by a gold mirror. The beam then passes through a diffractive optical element, forming a square-shaped top-hat beam. It is focused onto the sample surface by a 50 mm focal length aspherical ZnSe lens. The optical assembly as well as the camera with lens and distance sensor are mounted on aluminum rails and are in a fixed configuration. The only moving parts are the XYZ stages. During imaging, the laser focal point remains fixed in space, while the sample is moved. A specially designed gas funnel also serves as a focusing assembly and is connected to a 6/4 mm (O.D/I.D.) PTFE tube. The overpressure in the chamber drives a 10 L/min nitrogen gas flow through the tube. The laser ablation plumes are entrained into the gas and transported to the modified ion source (Bruker VIP HESI in the APCI configuration) of the Bruker Impact II mass spectrometer. The ion source also has a VUV source (Hamamatsu L12542) mounted axially to the MS sampling cone inside the ion source. An HPLC pump (Agilent G1312A) provides a steady flow of a solvent mixture (1% toluene in methanol; 200 μL/min) to the APCI needle.^21^ The settings of the ion source were as follows: APCI nebulizer, end plate offset 600 V, capillary 1000 V, corona 6000 nA, nebulizer 3.5 bar, dry gas 0.2 L/min, dry temperature 250 °C, probe gas temperature 350 °C, probe gas 4 L/min, exhaust turned on. MS^1^ experiments were performed in a scan range *m*/*z* of 47–1300.

#### 2.3.2 Direct Three-Dimensional Mass Spectrometry Imaging (MSI 3D)

The spatial resolution for the 3D MSI experiment was 140 μm, with applied oversampling. Each pixel/voxel in the 3D MSI experiments was exposed to the laser for 0.5 s, at a laser pulse repetition rate of 20 Hz. There were 1200 ms delays between pixels. Between pixels, the sample stage moved at a speed of 50 mm/s. The time delay between lines was 3 s. Each 3D experiment was carried out in the inverted pyramid ablation scheme.^21^

First 3D MSI experiment (Table 1) performed on sample containing *P. megaterium* and *F. avenaceum* had top ablation level of 43 × 43 (*X* × *Y*) voxel arrangement, second (lower) level −42 × 42 and third −41 × 41, with ablation levels at 4.40, 4.25, and 4.10 mm for top level and lower levels, respectively. The starting object was of ca. 4.4 mm thickness and 5.9 × 5.9 mm (*X* × *Y*) size. The objects were cut with a blade and placed on a stainless-steel plate, then on an ablation table inside the chamber, and frozen.

The second 3D MSI experiment (Table 1) performed on sample containing *B. licheniformis* and *F. avenaceum* had top ablation level of 35 × 35 (*X* × *Y*) voxel arrangement, second (lower) level −34 × 34, third −33 × 33 and fourth −32 × 32, with ablation levels at 4.10, 4.00, 3.90, 3.70 mm for top level and lower levels, respectively. The starting object was of ca. 4.1 mm thickness and 4.8 × 4.8 mm (*X* × *Y*) size. The objects were cut with a blade and placed on a stainless-steel plate, then transferred to an ablation table inside the chamber, and frozen.

### 2.4 Mass Spectrometry-Liquid Chromatography (LC–MS)

#### 2.4.1 LC–MS Sample Preparation

Metabolomic profiling was performed on both regions of each Petri dish with microorganism cocultures. Approximately 200 mg of the sample was weighed and cut into small pieces. To the sample pieces 150 μL of distilled water and 900 μL of acetone (Sigma, Aldrich, LC–MS grade) were added. At the same time, 3 stainless beads were added to the suspension, and the samples were homogenized 3 times for 1 min using a BeadBug 6 (Benchmark Scientific) at 4000 rpm. The samples were incubated at temp. 4 °C overnight. The next day, the samples were centrifuged (mySPIN 12 min Centrifuge, Thermo Fisher Scientific, temp. 4 °C, 14,000*g*, 5 min). The supernatant of each sample was collected, and the samples were left in a speed vac-type apparatus (2 × 10^–3^ mbar vacuum) overnight. The next day, the dried pellet was dissolved in methanol (800 μL). To facilitate dissolution, the samples were sonicated and then centrifuged for 5 min at 14,000*g*. The resulting supernatants were transferred to standard HPLC vials.

#### 2.4.2 LC–MS Metabolomic Analysis

Mass spectrometry-liquid chromatography analyses in positive and negative ion detection modes were performed on a Bruker Elute UHPLC system operated by Hystar 3.3 software and an ultrahigh-resolution (60,000+) mass spectrometer Bruker Impact II (Bruker Daltonik GmbH) ESI QTOF-MS equipped with Data Analysis 4.2 (Bruker Daltonik GmbH), TASQ (2022b) and Metaboscape (2022b). The ion source used was a Bruker VIP-HESI with optimized flows and temperatures. The column used for AutoMSMS measurements was a Waters Acquity UPLC BEH C18, with dimensions of 2.1 × 50 mm and 1.7 μm particle size. For AutoMSMS measurements, flows and percentages were: 0 and 0.56 min 99% A, 4.72 min–1% A, 5.56 min–1% A, 5.60, 6.34, and 9.45 min −99% A, all flows at 450 μLmin^–1^.^22−24^ The column was held at 40 °C.

The column exit was connected to the VIP HESI ion source. Internal calibration on 10 mM sodium formate (water: isopropanol 1:1 v/v) ions was performed automatically in Metaboscape with the use of a syringe pump at an infusion flow rate of 0.12 mL h^–1^, using a high precision calibration (HPC) mode. The autoMSMS method was used with *m*/*z* range 50–1500; CID (Collision-Induced Dissociation) was used with the following settings: absolute area threshold: 5000 counts; active exclusion 2 spectra; release after 0.3 min, isolation mass: for *m*/*z* = 100, width was 4, for 300 width was 5, for 500 was 6 and for 1000 was 8: 15, 10, 5 eV; collision energy value was 30 eV.

The untargeted annotations were performed in Metaboscape (ver. 2022b) with a criterion of mass deviation (Δ*m*/*z*) under 2 ppm and mSigma value under 30 as the maximum acceptable deviation of the mass of the compound and the isotopic pattern, respectively. All the molecular formulas were obtained using the Smart Formula tool and the C, H, N, O, P, S, Cl, Br, I, and F elements. MSMS spectra were automatically matched with MSMS libraries: Bruker HMDB 2.0 library, MassBank of North America (MoNA) library, and NIST ver. 2020 MSMS library. For compounds annotated in Metaboscape 2D and 3D ion images were generated (Tables 2 and 3). Compounds were identified by LARAPPI/CI-MSI based on the acquired LC–MS data and basic metabolites from the Ideom database and their representing ion images for microorganisms.

**Table 2:**
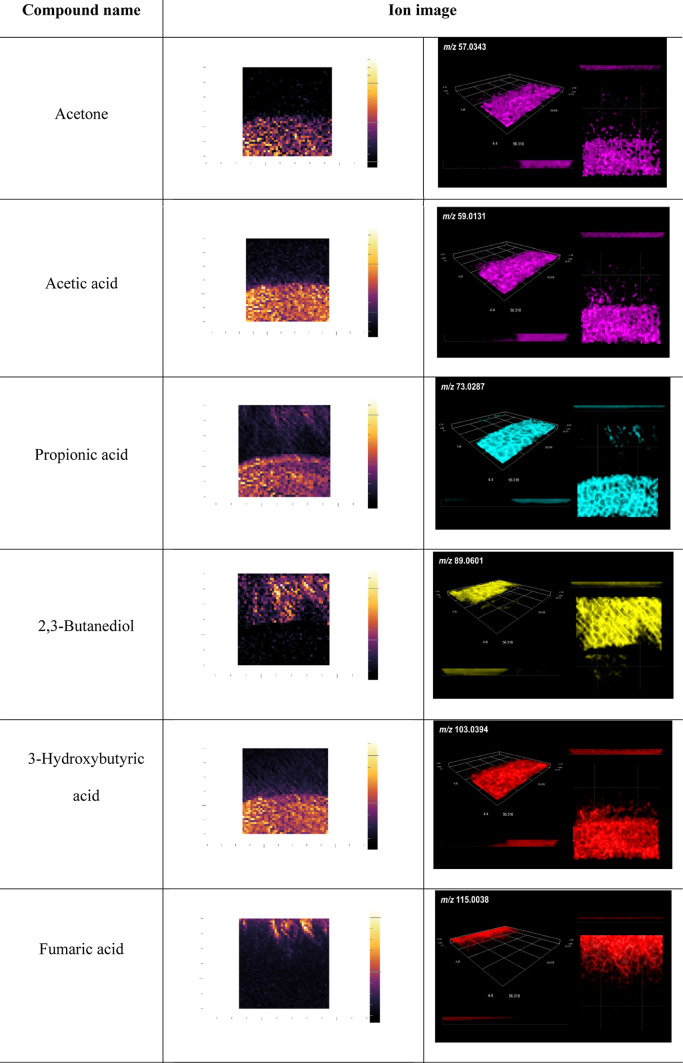

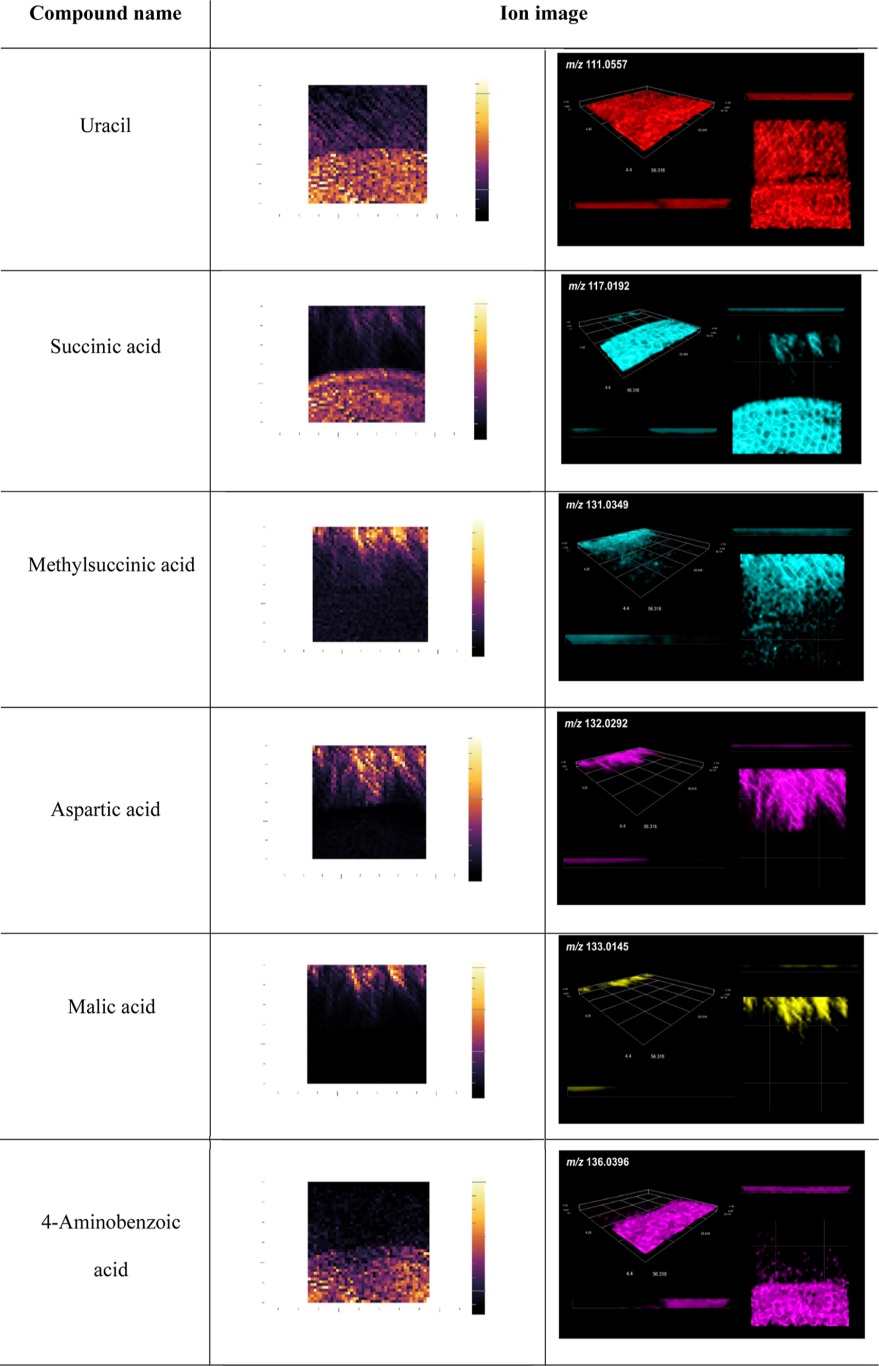

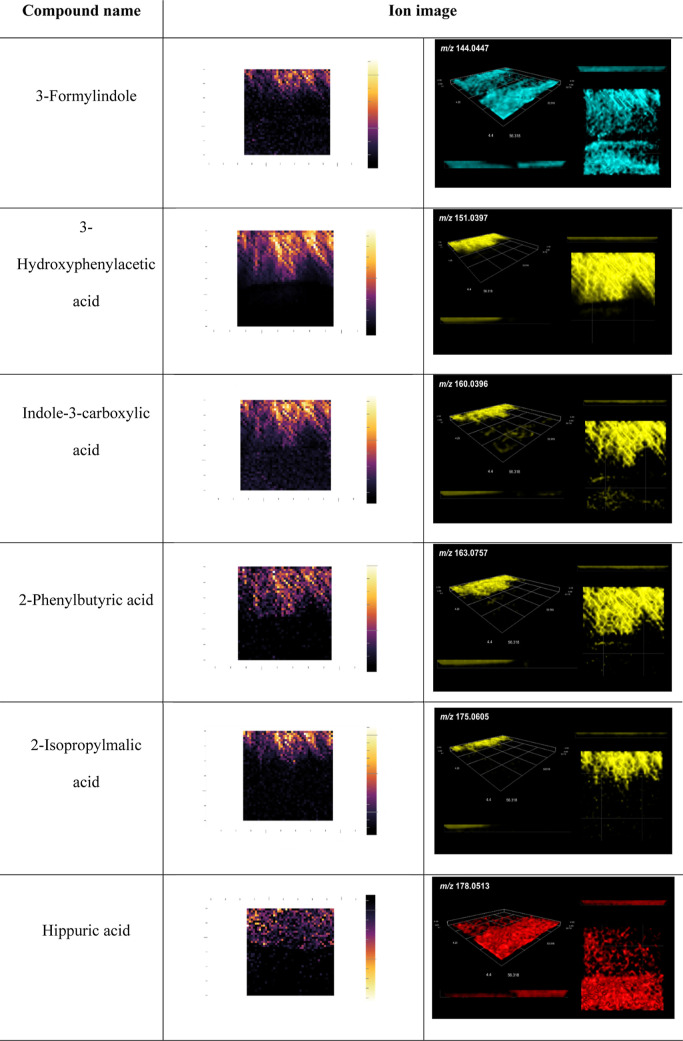

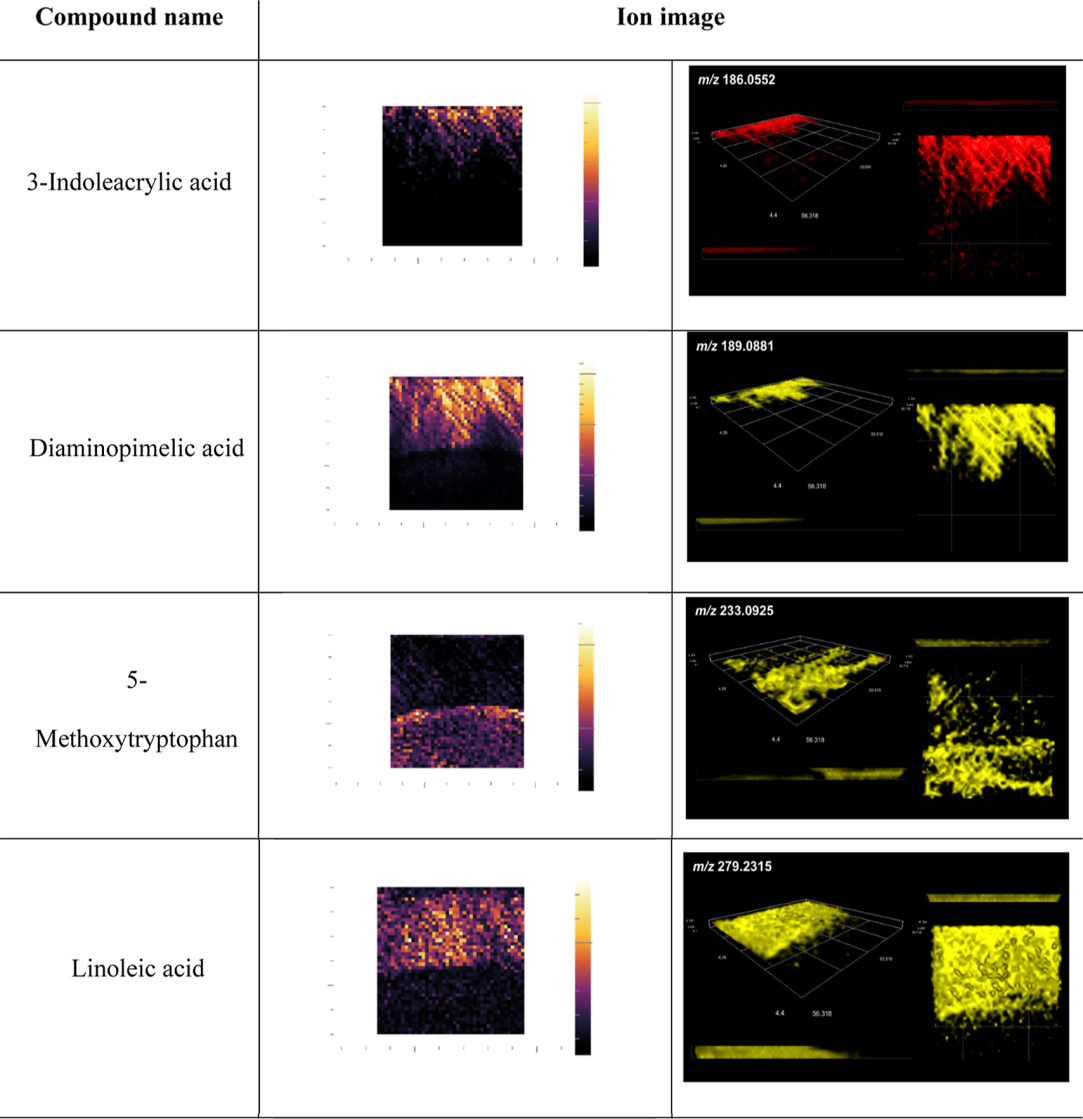
LARAPPI/CI-MSI 2D (Second Column) and 3D (Right Column) Ion Images of Metabolites of *P. megaterium* and *F. avenaceum*

**Table 3:**
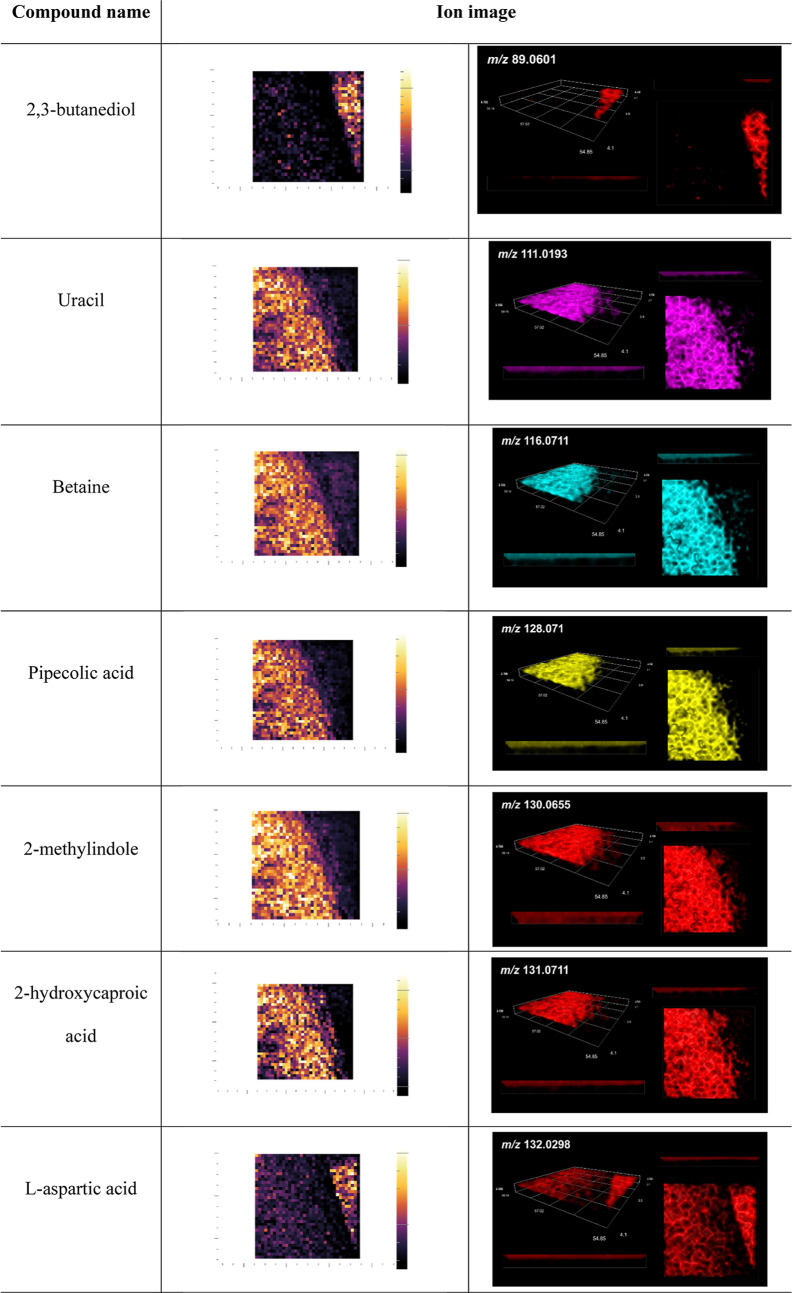

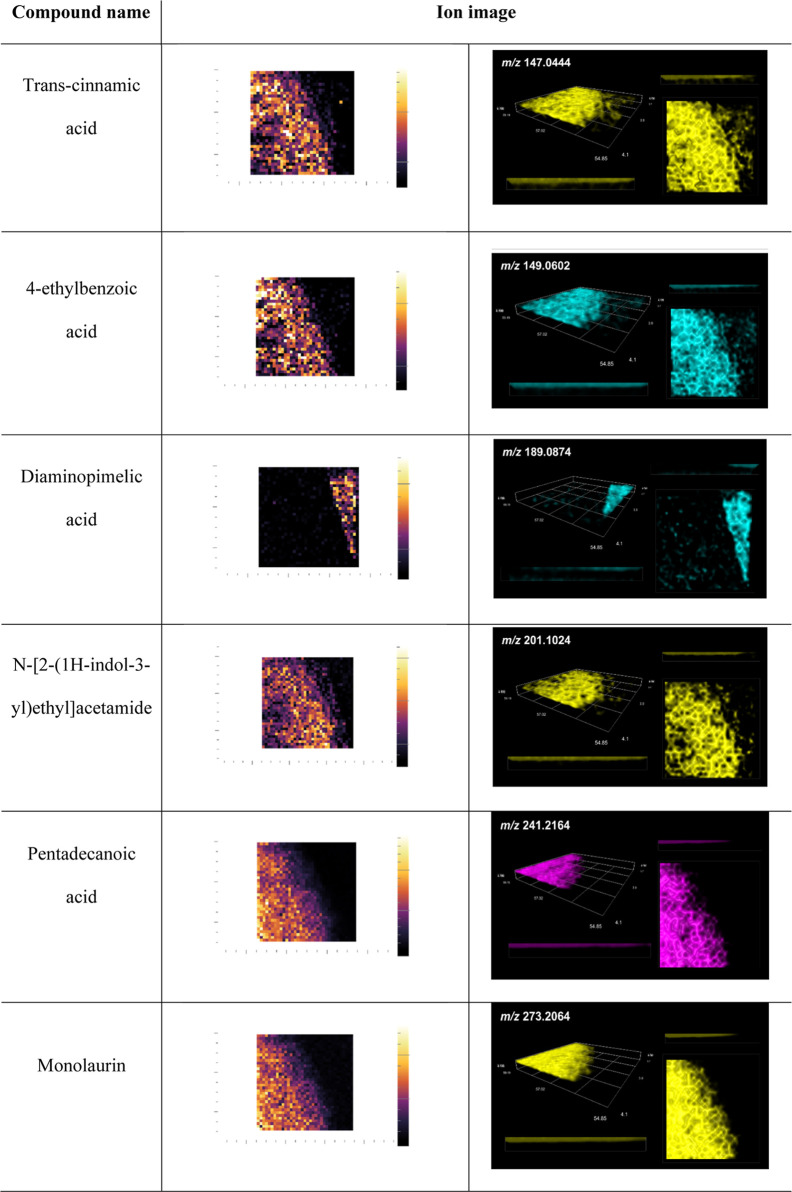

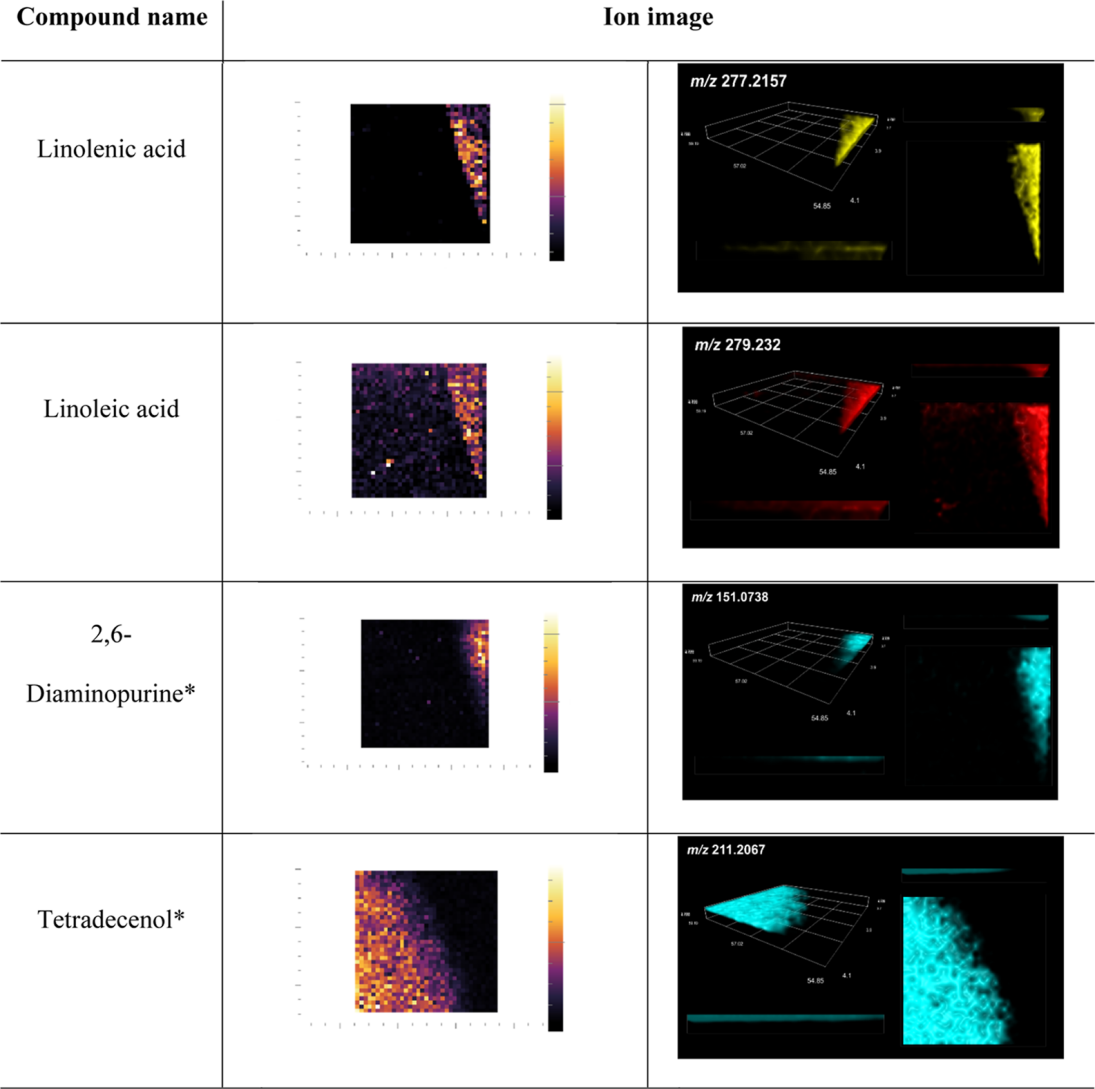
LARAPPI/CI-MSI 2D (Second Column) and 3D (Right Column) Ion Images of Metabolites of *B. licheniformis* and *F. avenaceum*^a^

a Compounds identified putatively based on precursor *m*/*z* matching.

#### 2.4.3 Statistical Analysis

All metabolite data sets exported from Metaboscape v.2022b (LC–MS data) were analyzed using MetaboAnalyst 6.0.^25^ The data were log-transformed and autoscaled before unsupervised Principal Component Analysis (PCA) and supervised Orthogonal Partial Least Squares Discriminant Analysis (OPLS-DA). Model validation was performed using permutation tests and 5-fold cross-validation. The VIP plots identified key metabolites responsible for group separation, with metabolites with VIP values above 1.0 considered the most significant. To identify affected metabolic pathways, pathway impact analysis was performed in MetaboAnalyst 6.0 using the KEGG pathway library for *Bacillus subtilis* subsp. *subtilis* and the Small Molecule Pathway Database (SMPD). Pathways were ranked based on statistical p-values, Holm-adjusted *p*-values, and FDR from pathway topology analysis.

## 3 Results and Discussion

This study presents the first examples of simple and direct 3D MSI of microbial cultures. LARAPPI/CI-3D-MSI system contains diffractive optical element that produces flat-bottom square ablation craters. As we have shown earlier,^21^ single point ablation of agar medium produced the desired square shape with rounded edges of 170 × 170 μm size. For the need of 3D MSI, ablation of the region of interest area must produce relatively flat area that will be suitable for controlled ablation of lower layers. Optimization of oversampling suggested that 140 μm voxel-center-to-voxel-center produced optimal results with a relatively flat bottom of 330 μm depth by using 20 laser shots per voxel. For the need of 2D MSI, the voxel stacking is not needed, therefore resolutions may range from 140 μm to few millimeters depending on experiment time frame and object size.

### 3.1 Identification of Microbial Metabolites Using LARAPPI/CI-3D-MSI

Ion images of metabolites from microorganisms that are key to interspecies interactions, localized in three dimensions using LARAPPI/CI-3D-MSI and supported by acquired LC–MS data (Table S8), are presented in Tables 2 and 3. All identified compounds from the uppermost layer and 3D visualizations of their locations are showed in Tables S1 and S2 (Supporting Information).

The use of LARAPPI/CI-3D-MSI allowed us to identify the compounds produced by the tested microorganisms and to determine their locations in the tested area, as well as whether they have the ability to diffuse into the agar medium. The following chemical compounds were identified using this method from *P. megaterium*: acetic acid, acetone, 4-aminobenzoic acid, 3-hydroxybutyric acid, 5-methoxytryptophan, propionic acid, succinic acid, uracil (Table 2), and others (Table S1).

For *B. licheniformis*, the most important identified metabolites were betaine, 2-hydroxycaproic acid, 2-methylindole, 4-ethylbenzoic acid, monolaurin, *N*-[2-(1*H*-indol-3-yl)ethyl]acetamide, pipecolic acid, pentadecanoic acid, tetradecenol, trans-cinnamic acid (Table 3). Others are listed in Table S2.

Based on the obtained ion images for *F. avenaceum*, the following compounds were identified: aspartic acid, 2,3-butanediol, diaminopimelic acid 2,6-diaminopurine, 2-isopropylmalic acid, fumaric acid, hippuric acid, 3-hydroxyphenylacetic acid, malic acid, linoleic acid, linolenic acid, 2-phenylbutyric acid (Tables 2 and 3) and others (Tables S1 and S2).

Various techniques have been developed for imaging microbial colonies (mainly optical imaging, ratio metric imaging, Raman spectrometry). However, these methods provide only limited molecular and spatial information and carry the risk of metabolite delocalization during the imaging process. In turn, MSI methods most often assume, for simplicity, that colonies are flat, inadvertently omitting valuable details.^26^ This has led to the emergence of new solutions for 3D analysis of biological objects. Shen et al.^26^ proposed moisture-assisted cryo-section (MACS) combined with MALDI-MSI. The key to this method is the sectioning of colonies parallel to the growth plane, under controlled humidity conditions, without an embedding procedure. With this technology, the authors visualized 3D spatial details of the distribution of molecules within the colony and in the bacterial biofilm structure. The innovative LARAPPI/CI MSI method used in the present study was developed recently by Ruman et al.^21^ and employed for the first direct three-dimensional (3D) mass spectrometry imaging (MSI) of metabolites in human and plant tissues. The greatest advantage of this method is that it does not require any sample modification or thin-slicing. Instead, individual layers of the material are removed in a strictly controlled manner (inverted pyramid ablation) and immediately transferred to the mass spectrometer for analysis. This represents significant progress compared to currently used cutting-edge methods of 3D MSI.

### 3.2 Biological Activity of Visualized Compounds

Acetic and propionic acids are weak organic acids that inhibit the growth of microorganisms, including the bacteria *Arcobacter*, *Listeria*, the fungi *Saccharomyces*, *Zygosaccharomyces*, and *Candida*, and phytopathogens such as *Colletotrichum* sp. and *Aspergillus flavus*. Acetic and propionic acids are commonly used as preservatives in various food products.^27−29^ The ion images indicate diffusion of these acids into the agar medium (Table 1). Similarly, 3-hydroxybutyric acid has been shown to exhibit some antimicrobial, insecticidal, and antiviral activities.^30−32^ The poly(3-hydroxybutyric acid) oligomer with a few degrees of polymerization also possesses effective properties against *Staphylococcus aureus*, *Klebsiella pneumoniae,* and *Candida albicans*.^33^

Acetone is an organic solvent that can be produced by bacterial cells. Park^34^ showed that the presence of 5% acetone in a culture medium causes slight inhibition of mycelium growth and germination of fungal spores, while 10% acetone has a significant inhibitory effect.

4-Aminobenzoic acid, (*p*-aminobenzoic acid) plays a crucial role in the metabolic pathways of microorganisms. Additionally, it has been shown to have direct antibacterial activity against *Listeria monocytogenes*, *Salmonella enterica*, and *Escherichia coli*, with low pH values enhancing its effectiveness. It also exhibits synergistic antibacterial potency when combined with several antibiotics against various bacterial strains, including *S. aureus* and *Pseudomonas aeruginosa*.^35,36^ Previous studies have shown that 5-methoxytryptophan has weak antifungal activity against *Physalospora piricola*.^37,38^

The antimicrobial activity of succinic acid has been applied for the preservation of poultry and beef meat.^39,40^ Interestingly, the ion images obtained in our study indicate that succinic acid occurs mainly in *Priestia* bacteria, and is present at much lower levels in *Fusarium* fungi. Its methylated derivative (methylsuccinic acid) occurs in colonies of *F. avenaceum* fungi (Table 2).

Uracil was detected in both the *B. licheniformis* vs *F. avenaceum* and *P. megaterium* vs *F. avenaceum* pairs. Uracil and its analogues have important applications in clinical medicine as chemotherapeutic agents. Its analogues (e.g., bromacil, lenacil, and terbacil) are commercial herbicides. Various derivatives have antimicrobial activity.^41^

Betaine is an osmotic agent for many bacteria, and has antimicrobial properties. It is found in animals, plants, and other eukaryotes.^42,43^ It is also a biostimulant that effectively supports plants in stressful situations and has applications in agriculture. Similarly, pipecolic acid has potential applications in a wide range of agricultural plants, due to the increased resistance of various plant species to stressful environmental conditions.^44^

Previous studies have shown 2-hydroxyisocaproic acid to have a broad spectrum of antimicrobial activity against bacteria and fungi, as well as anti-inflammatory properties. This compound belongs to the oxylipins, which have a significant impact on the reproduction and development of fungi, as well as on their adaptative responses and synthesis of secondary metabolites. Oxylipins have confirmed antifungal activity and also operate via mechanisms that resemble quorum sensing. Because of this, they play an important role in microbial interaction.^45^ The European Food Safety Authority (EFSA) has approved 2-hydroxyisocaproic acid as a flavoring agent in nutritional products.^46^

According to the literature, 4-ethoxybenzoic acid exhibits antipathogenic antibiofilm activity and decreases the hydrophobicity of *S. aureus*.^47^ Pentadecanoic acid has similar antibiofilm properties, including against *K. pneumoniae* bacteria and *C. albicans* fungi.^48^ Monolaurin is known to have antimicrobial activity (antibacterial, antiviral and antifungal), including effect against *C. albicans*, *C. tropicalis*, *Aspergillus niger* and *Fusarium* sp.^49^ Helal et al.^50^ suggest that tetradecanol has activity against plant pathogenic bacteria and fungi and report a positive effect on plant growth of tomato seedlings. *Trans*-cinnamic acid has been reported to exert antimicrobial effects on many microorganisms, and is widely used as a raw material for pesticides, as growth promoters, and in long-acting fungicides and preservatives for fruits and vegetables. Li et al.^51^ and Yilmaz et al.^52^ suggest that it can be used as an environmentally friendly alternative additive to prevent and control fish pathogens. The diffusion of this compound is visible in the ion images, so it is of key importance as a biocontrol agent for the tested *B. licheniformis* strain.

3D imaging also allowed the detection of indole derivatives, including *N*-[2-(1H-Indol-3-yl)ethyl]acetamide in the *B. licheniformis* colony and indole-3-acrylic acid, indole-3-carboxylic acid, 3-formylindole, and 2-methylindole in the *F. avenaceum* colony. According to the literature, indole derivatives may exhibit bioactivity.^53^ Some of the indole derivatives identified by 3D imaging (e.g., indole-3-acetic acid) are metabolites produced by plant pathogens from the *Fusarium* strain isolated from rhizospheric soil samples. These compounds belong to the class of phytohormones, which play an important role in plants, especially in root initiation and elongation, tissue differentiation and proliferation, and mediation of fungal–plant interaction.^54^

Among the fungal metabolites, it is worth paying attention to aspartic acid, which has potential antibacterial and antifungal effects.^55^ Its ionic image reflects the macroscopic image of mycelial hyphae on the surface of agar medium. Similarly to linoleic acid, this compound occurs commonly in *Fusarium* sp. and has been considered as a biomarker of this type of fungi.^56^ Both linoleic and linolenic acids identified using MSI have broad antibacterial potential against both Gram-positive and Gram-negative bacteria.^57^

An interesting case is 2,3-butanediol, which belongs to short chain alcohols and volatile compounds. 2,3-butanediol is also an important inducer of plant defenses and mediates plant-beneficial effects, such as growth promotion.^58^ This compound shows activity against plant pathogens such as *Botritis cinerea*, *Monilinia fruticola*, *M. laxa*, *Penicillium digitatum*, *P. expansum*, *Trichoderma* sp. and *Ralstonia solanacearum*.^58,59^ The ion images (Tables 2 and 3) indicate higher concentrations of this metabolite in the mold *F. avenaceum* than in the tested bacteria. However, according to the literature it is most often produced by rhizosphere bacteria, mainly from the genera *Aerobacter*, *Serratia*, *Enterobacter*, and *Klebsiella*.^58^ Shandeep et al.^60^ confirmed the production of 2,3-butanediol by *Bacillus megaterium* (now *P. megaterium*). However, it has been suggested that fungi as well as bacteria can produce three stereoisomers (2R,3R-, 2S,3S- and 2R,3S-) of 2,3-butanediol which can be significant for microbial interactions.^61^ This most likely indicates the low specificity of this metabolite. Good diffusion of this compound was observed in the agar medium (Tables 2 and 3). It should therefore be considered to have high application potential.

Diaminopimelic acid is a constituent of the bacterial and fungal cell wall structure and is found in higher plants.^62^ This compound dominated in the ion images obtained for *Fusarium* (Tables 2 and 3). In the mycelium of *F. avenaceum*, 2,6-diaminopurine was one of the first purine analogues found to inhibit bacterial growth as well as virus reproduction (polio).^63^

2-isopropylmalic acid is an intermediate for the biosynthesis of leucine in fungi. Therefore, its presence in the ion images for *Fusarium* is not surprising. This compound can be biologically active against several food-borne pathogens.^64^ Malic acid has antibacterial activity that has been previously proven against *L. monocytogenes*, *S. enterica*, and *E. coli* O157:H7.^65^ Like some other organic acids, malic acid stimulates the production of *Fusarium* antagonists in the rhizosphere.^66^ Unver^67^ showed that fumaric acid has a potent inhibitory effect against pathogenic and opportunistic microorganisms, including bacteria belonging to genera *Escherichia*, *Enterobacter*, *Klebsiella*, *Pseudomonas*, and *Candida yeast*. It also has potential analgesic, anti-inflammatory, antioxidant, antipsoriatic, chemopreventive, immunomodulatory, and neuroprotective properties.^67^

Among the metabolites detected for *Fusarium*, we also found 3-hydroxyphenylacetic acid (belonging to phenolic acids). This compound has shown a dose-dependent antimicrobial effect, including against *P. aeruginosa* and *Staphylococcus epidermidis*.^68^ Hussein Al Ali et al. observed that *P. aeruginosa* and methicillin-resistant *S. aureus* were more sensitive to hippuric acid compared to *B. subtilis* and *S. enterica*.^69^ 2-Phenylbutyric acid shows inhibitory activity against bacteria, including the plant pathogens *Xanthomonas vesicatoria*, *Pectobacterium carotovorum*, *R. solanacearum*, and *Xanthomonas oryzae*.^70^

### 3.3 Characteristic of the Metabolome of the Tested Microorganisms

A total of 29,017 *m*/*z* values were obtained in positive ion mode and 3040 were obtained in negative ion mode. Across both modes, 1771 chemical compounds were identified. The data from both ion modes were combined and analyzed using the MetaboAnalyst 6.0 platform. PCA and heat map analyses were performed on the entire data set.

**Figure 1:**
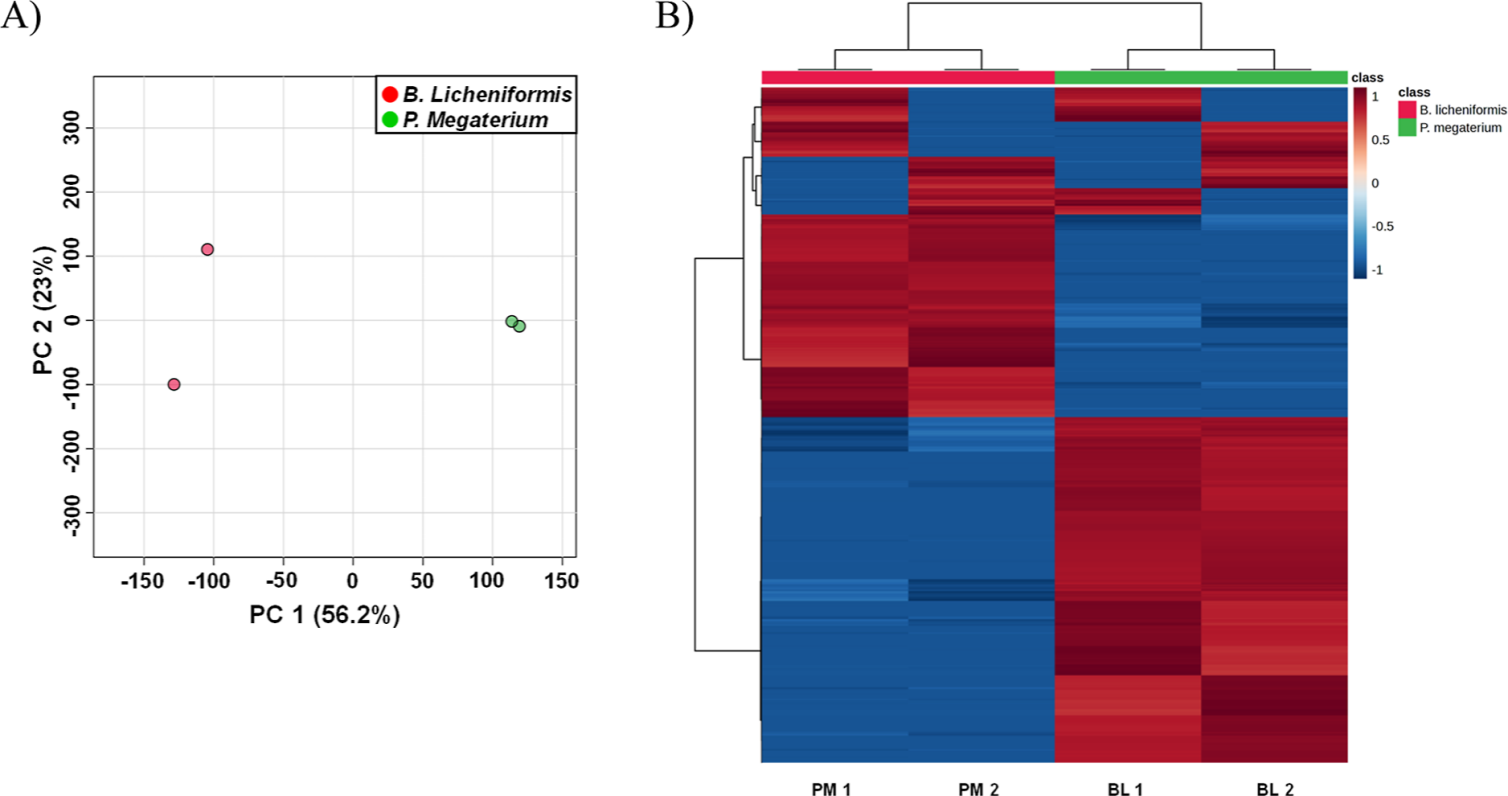
Statistical analysis of the entire MS data set. (A) Principal Component Analysis (PCA) score plot of metabolites from *B. licheniformis* (red dots) and *P. megaterium* (green dots) cocultures with *F. avenaceum*; (B) Heatmap of metabolite abundance distinguishing *B. licheniformis* and *P. megaterium* cocultures with *F. avenaceum*. Red indicates higher relative abundance, blue indicates lower relative abundance. The color bar on the right represents the class labels for *B. licheniformis* (red) and *P. megaterium* (green).

The Principal Component Analysis (PCA) score plot of metabolites from cocultures of *B. licheniformis* and *P. megaterium* with *F. avenaceum* shows the separation between the two bacterial species based on the first two principal components (PC1 and PC2), which together explain 79.2% of the total variance (Figure 1A). *B. licheniformis* samples are distinctly separated from *P. megaterium* samples, indicating clear metabolic differences between the two species. The heatmap illustrates the hierarchical clustering of samples from *B. licheniformis* (BL1, BL2) and *P. megaterium* (PM1, PM2) based on the normalized abundance of metabolites (Figure 1B). The clustering dendrogram at the top shows clear separation between the two species, with distinct metabolic profiles. Principal Component Analysis (PCA) shows the separation between the two bacterial species based on the first two principal components (PC1 and PC2), which together explain 79.2% of the total variance (Figure 1A). *B. licheniformis* samples are distinctly separated from *P. megaterium* samples, indicating clear metabolic differences between the two species. The heatmap illustrates the hierarchical clustering of samples from *B. licheniformis* (BL1, BL2) and *P. megaterium* (PM1, PM2) based on the normalized abundance of metabolites (Figure 1B). The clustering dendrogram at the top shows the clear separation between the two species, with distinct metabolic profiles.

To identify the compounds that most significantly differentiated the two groups, criteria of VIP > 1, *p*-value and FDR < 0.05, and fold-change (FC) > 2 or <0.5 were applied. A total of 351 compounds met these criteria, of which 151 were significantly more abundant in *B. licheniformis* and 200 were more abundant in *P. megaterium* cocultures with *F. avenaceum*. For the identified compounds, OPLS-DA, *t* tests, fold-change analysis, and volcano plots were generated.

**Figure 2:**
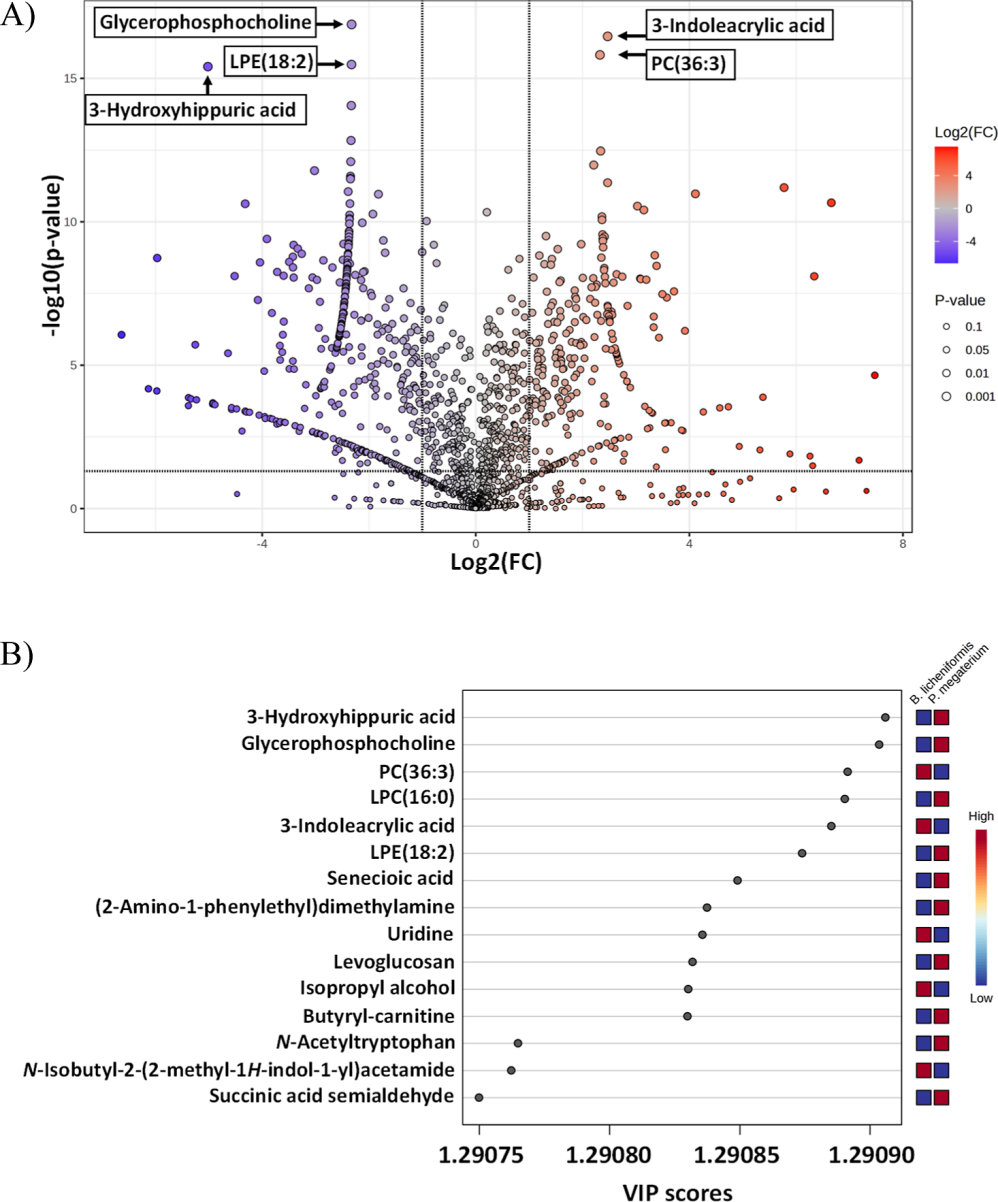
Statistical analysis of the identified compounds. (A) Volcano plot, the *X*-axis (log2FC) represents the log-transformed fold change in expression, the *Y*-axis (−log10(*p*)) indicates the statistical significance (*p*-value) of each compound. Red points represent compounds that are significantly more abundant in *B. licheniformis*, blue points represent compounds that are significantly more abundant in *P. megaterium*, gray points represent compounds under the significance threshold. (B) VIP score plot showing OPLS-DA analysis. The metabolites on the *Y*-axis are ranked based on their Variable Importance in Projection (VIP) scores, as shown on the *X*-axis. Higher VIP scores indicate a greater contribution to the separation between the two bacterial species. The heatmap on the right indicates the relative abundance of each metabolite in samples, with red representing high abundance and blue representing low abundance.

A volcano plot depicting the differential expression of biological compounds between *B. licheniformis* and *P. megaterium* cocultures with *F. avenaceum* is presented in Figure 2A. The volcano plot indicates mainly glycerophosphocholine, LPE(18:2) and 3-hydroksyhippuric acid compounds as compounds significantly more abundant in *P. megaterium*. In the case of *B. licheniformis*, the statistical analysis shows the most differentiating compounds are 3-indoleacrylic acid and PC(36:3) (Figure 2A). A VIP score plot showing the most discriminating metabolites between *B. licheniformis* and *P. megaterium* bacteria cocultures with *F. avenaceum* is shown in Figure 2B. The results of the VIP score plot and volcano plot overlap partially. In addition to the compounds differentiating both study groups described above, LPC (16:0) was found to differentiate the bacteria cocultures (Figure 2B).

The characteristics of key metabolites discriminating between *B. licheniformis* and *P. megaterium* samples are shown in Table S3 (Supporting Information). The table lists the metabolites with the highest scores identified through VIP (Variable Importance in Projection), along with their molecular formulas, experimental mass-to-charge ratios (*m*/*z* exp.), retention times (RT), ionization modes (positive or negative), VIP scores, p-values, and fold changes between the *B. licheniformis* and *P. megaterium* samples.

The presented statistical analysis of the entire MS data set will be useful for the rapid comparison of metabolic profiles of potential biocontrol agents from different species and strains belonging to the same species but, for example, isolated from different environments that may differ in terms of their metabolic properties.^71^

After LC–MS analysis in positive and negative mode, 334 chemical compounds were identified for the *B. licheniformis*–*F. avenaceum* research system, of which 236 metabolites were recognized in the HMDB database in MetaboAnalyst and included in the further analysis using the *B. subtilis* subsp. *subtilis* 168 library (KEGG). For the *P. megaterium*–*F. avenaceum* system, 374 chemical compounds were identified, of which 261 metabolites were recognized in the HMDB database in MetaboAnalyst and further analyzed.

**Figure 3:**
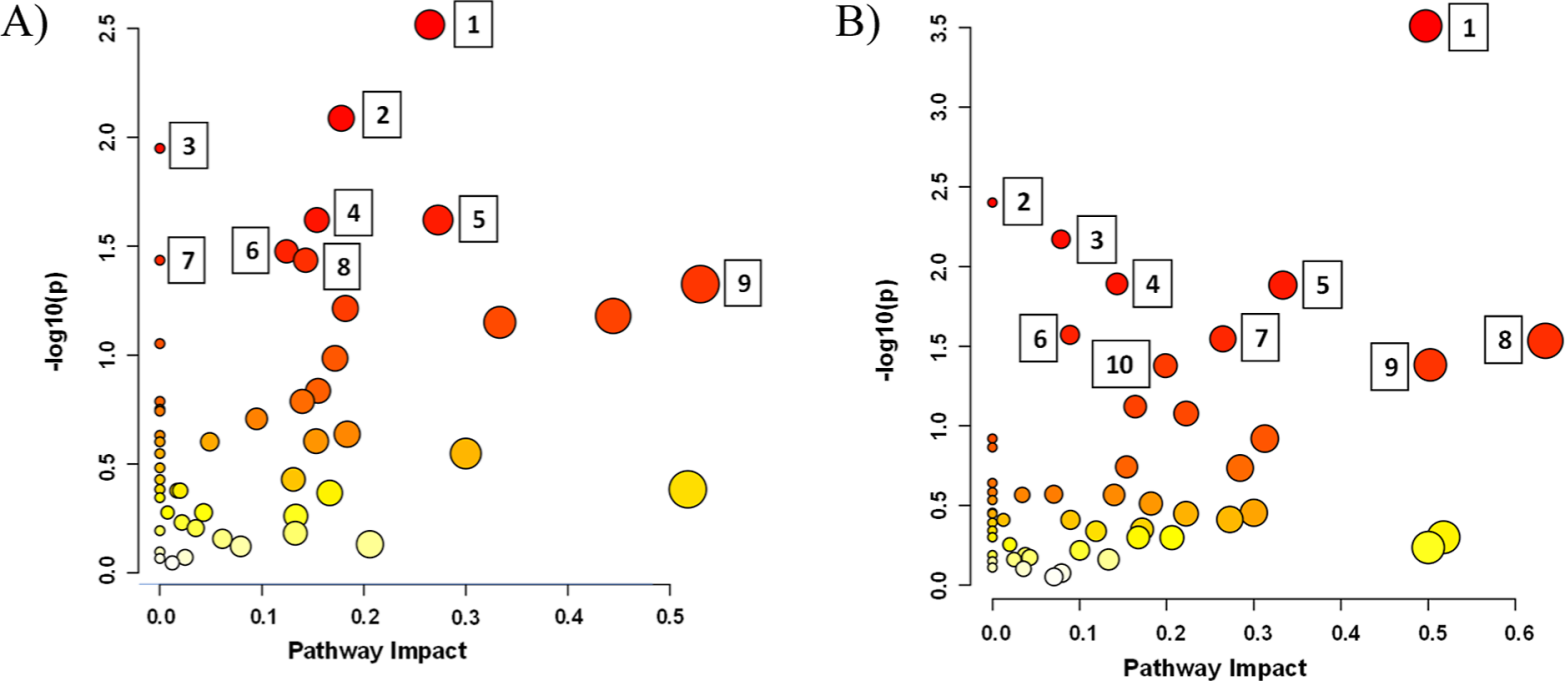
Pathway Impact Analysis of Metabolites. (A) In *B. licheniformis* cocultures with *F. avenaceum*: (1) glycine, serine, and threonine metabolism; (2) d-amino acid metabolism; (3) cyanoamino acid metabolism; (4) histidine metabolism; (5) arginine biosynthesis; (6) methane metabolism; (7) vitamin B6 metabolism; (8) Taurine and hypotaurine metabolism; (9) alanine, aspartate, and glutamate metabolism; (B) in *P. megaterium* cocultures with *F. avenaceum*: (1) arginine biosynthesis; (2) tyrosine metabolism; (3) valine, leucine and isoleucine biosynthesis; (4) taurine and hypotaurine metabolism; (5) pyruvate metabolism; (6) d-amino acid metabolism; (7) purine metabolism; (8) alanine, aspartate, and glutamate metabolism; (9) arginine and proline metabolism; (10) glycine, serine, and threonine metabolism. The bubble plot shows impacted metabolic pathways, based on pathway impact scores (*X*-axis) and −log10(*p*) values (*Y*-axis). Larger and more intense red bubbles represent pathways with higher impact and statistical significance. Pathways numbered in the plot correspond to those with the greatest influence and significance in distinguishing between the groups.

Pathway Impact Analysis of Metabolites of the studied systems is presented in Figure 3. In both study cocultures pairs, the most statistically significant pathways were arginine biosynthesis, taurine and hypotaurine metabolism, and d-amino acid metabolism, as well as alanine, aspartate, and glutamate metabolism. For *B. licheniformis*–*F. avenaceum*, the most important pathways were as follows: glycine, serine, and threonine metabolism; d-amino acid metabolism; cyanoamino acid metabolism; histidine metabolism; arginine biosynthesis; methane metabolism; vitamin B6 metabolism; taurine and hypotaurine metabolism; alanine, aspartate, and glutamate metabolism pathways (Figure 3A).

For *P. megaterium*, the most important pathways were as follows: tyrosine metabolism; valine, leucine, and isoleucine biosynthesis; pyruvate metabolism; purine metabolism; arginine and proline metabolism; glycine, serine, and threonine metabolism (Figure 3).

**Figure 4:**
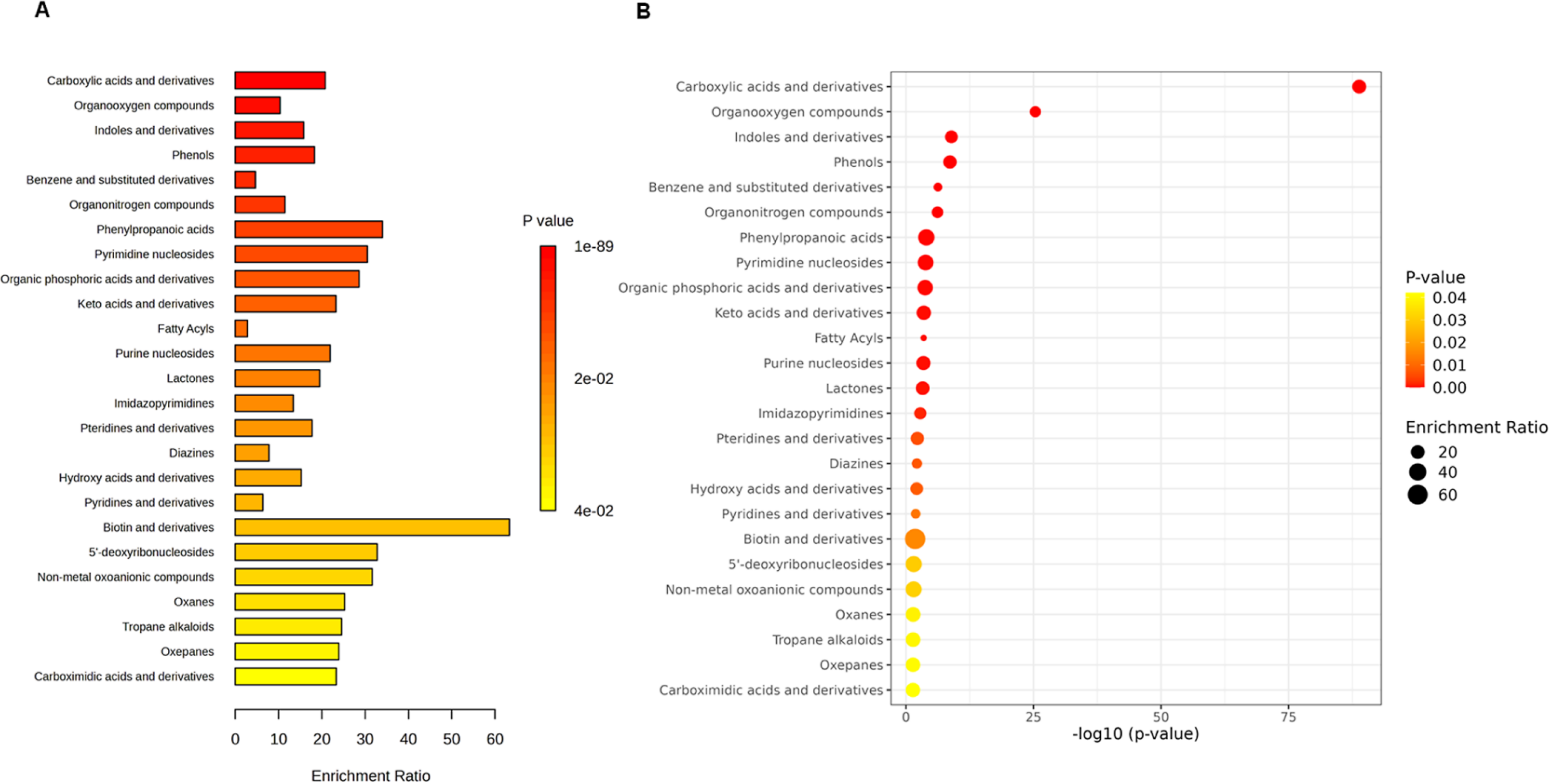
Pathway enrichment analysis results for *B. licheniformis* cocultures with *F. avenaceum*, based on chemical compound classes. (A) Bar plot of enriched chemical classes showing the enrichment ratio for each class. (B) Bubble plot illustrating the pathway impact with the corresponding −log10(*p*-value). Larger bubbles represent higher enrichment ratios. The color scale indicates statistical significance (*p*-value), with red indicating higher significance.

**Figure 5:**
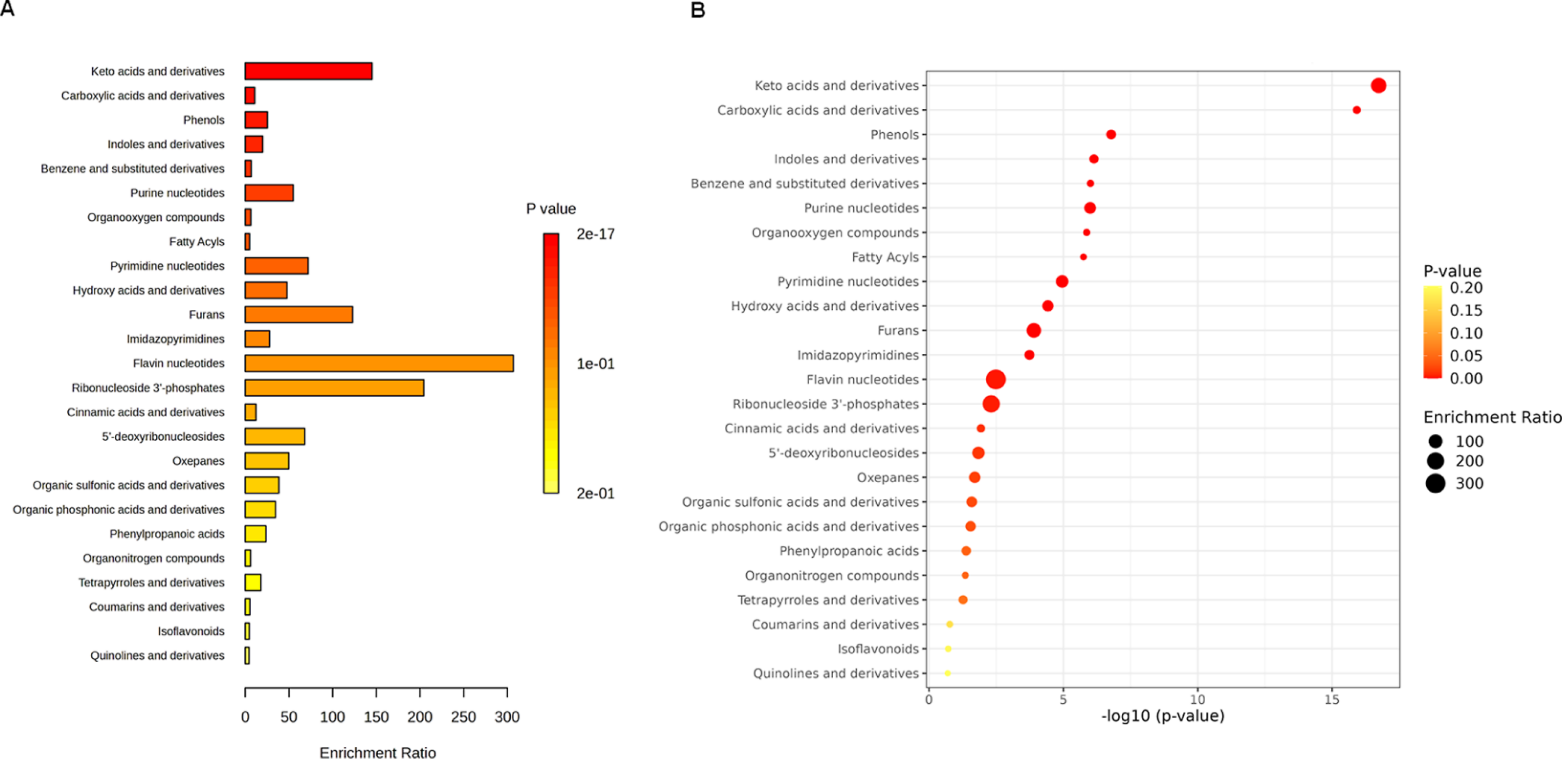
Pathway enrichment analysis results for *P. megaterium* cocultures with *F. avenaceum*, based on chemical compound classes. (A) Bar plot of enriched chemical classes showing the enrichment ratio for each class. (B) Bubble plot illustrating the pathway impact with the corresponding −log10(*p*-value). Larger bubbles represent higher enrichment ratios. The color scale indicates statistical significance (*p*-value), with red indicating higher significance.

All identified metabolic pathways highlighting the matched pathways, key metabolites, and statistical significance for each metabolic pathway are provided in Tables S4 and S5 (Supporting Information). Figures 4 and 5 show pathway enrichment analysis results for the tested microorganisms.

In the case of *B. licheniformis* cocultures with *F. avenaceum*, the identified metabolites belonged to 33 metabolite sets (Table S6, Supporting Information). For the *P. megaterium*–*F. avenaceum* pair, there were 29 metabolite sets (Table S7, Supporting Information), In both systems, most of the identified compounds can be classified as carboxylic acids and derivatives (88 and 22 hits, accordingly), organooxygen compounds (11–37 depending on the system), benzene and substituted derivatives (11–16), or fatty acyls (13–15). An analysis of metabolic pathways of the compounds that most differentiated both bacteria (based on the statistical analysis described below) is presented in Appendix A.

It is worth noting that the majority of compounds in both sets of cocultures were carboxylic acids and derivatives. According to the literature, natural carboxylic acids show good biological activity.^72^ Sodium and potassium salts of aliphatic and aromatic carboxylic acids have been shown to have antimicrobial effects on the growth of common food spoilage and pathogenic microorganisms from genera of *Leuconostoc*, *Lactobacillus*, *Enterococcus*, *Candida*, *Pseudomonas*, *Salmonella*, and *Listeria*.^73^ The antimicrobial properties of carboxylic acids depend on many physicochemical factors, including polarizability, molecule shape and size, the presence of rotating bonds, hydrophilicity and lipophilicity, dipole moment, and solubility. These factors determine the type and strength of interactions between carboxylic acids and various biological molecules (including the cell membrane, cell wall, proteins, and DNA). The biological effect carboxylic acids is very complex and may vary depending on the physiological conditions and bacterial strain.^72^

Elbalola & Abbas^74^ report that the organooxygen compound content of plant extracts has a positive correlation with antibacterial activity against *S. aureus* and *E. coli*. Benzene and substituted derivatives and fatty acyls also belong to groups of chemical compounds rich in metabolites of microbial origin which exhibit antibacterial and antifungal activity.^75,76^

It should be remembered that the antimicrobial activity of microorganisms is determined not by a single metabolite, but by the total pool of all bioactive compounds, which often demonstrate synergistic effects.^77^

### 3.4 Advantages of LARAPPI/CI-3D-MSI: Comparison with 2D MSI and Alternative Ionization Strategies

LARAPPI/CI-3D-MSI provides a unique combination of ambient-pressure operation, minimal sample preparation, dual ionization source and volumetric imaging capabilities, setting it apart from conventional 2D MSI methods such as MALDI, DESI, or SALDI. Compared to MALDI and SALDI, which often require vacuum conditions, matrix application, or particulate coatings, LARAPPI/CI simplifies the workflow by eliminating the need for extensive sample preparation (e.g., matrix coating, ultrathin slicing, or dehydration). Its mid-infrared laser ablates hydrated samples at or near ambient conditions, preserving morphological and chemical features. Unlike DESI, MALDI and SALDI, LARAPPI/CI offers much deeper medium sampling (up to 0.3 mm per layer) through relatively high energy infrared laser pulses, making it especially suitable for tissues and agar-based media with high moisture content.

In terms of ionization coverage, LARAPPI/CI uses atmospheric-pressure chemical ionization (APCI) and/or photoionization, which allows detection of broad range of small molecules. While ESI/DESI and MALDI excels at ionization of polar and charged species, these methods can struggle with medium-low-polarity compounds often found in microbial secondary metabolism. MALDI may also introduce strong interference in the low-mass range (below *m*/*z* 800) due to matrix signals, a limitation especially relevant for small-molecule metabolites in agar-based cultures. This limitation is particularly relevant for the studied system, where microbial interactions rely on the diffusion and bioactivity of small secondary metabolites. LARAPPI/CI bypasses these issues by ionizing gas-phase molecules ablated directly from the hydrated substrate, reducing the risk of ion suppression, adduct formation, or loss of volatile compounds.

A key advantage of LARAPPI/CI-3D-MSI lies in its capacity to generate three-dimensional metabolite maps without subjecting the sample to vacuum or prolonged drying steps. While 2D MSI reveals surface-level distributions, it cannot capture vertical diffusion or deeper-layer processes—crucial for understanding how bioactive metabolites penetrate agar or tissue matrices. By ablating successive layers, LARAPPI/CI-3D-MSI uncovers hidden gradients of antimicrobial and signaling compounds that may influence microbial colony development, pathogen suppression, or other interspecies interactions. For biocontrol applications, this depth-resolved view is essential because many pathogens and their antagonists interact through metabolite gradients extending well below the surface. It is known that microorganisms can form multilayered colonies, biofilms, or hyphal networks with structural complexity that extends beyond a plane. 3D MSI makes it possible to visualize these spatial details by progressively ablating layer after layer (or slicing, in older approaches) to reveal how metabolites are distributed at each depth. Such data can clarify, for instance, where in the agar a specific metabolite is most concentrated or how deeply a fungus has colonized the medium compared to a bacterial antagonist. Traditional 2D methods are prone to missing these vertical dynamics, especially when colonies form biofilms or grow into the medium over time.

## Data Availability

Data is provided within the manuscript or Supporting Information files. The data sets generated during and/or analyzed during the current study are available from the corresponding author upon request and in the RepOD open data repository (DOI: 10.18150/CIGKAJ).

## Appendix A

Pathway analysis for the most differentiating compounds for *Bacillus licheniformis* and *P. megaterium* cocultures with *F. avenaceum*.

## Supporting Information Available

Table S1: compounds identified by LARAPPI/CI-MSI based on acquired LC–MS data and basic metabolites from Ideom database and their representing ion images for microorganisms *P. megaterium* and *F. avenaceum*; Table S2: compounds identified by LARAPPI/CI-MSI based on acquired LC–MS data and basic metabolites from Ideom database and their representing ion images for microorganisms *B. licheniformis* and *F. avenaceum*; Table S3: key metabolites discriminating between *B. licheniformis* and *P. megaterium* cocultures with *F. avenaceum*. A fold change greater than 2 indicates a significantly higher abundance of the metabolite in *B. licheniformis*, a fold change below 0.5 indicates a significantly higher abundance in *P. megaterium*; Table S4: pathway enrichment analysis of metabolites in *B. licheniformis*, highlighting the matched pathways, key metabolites, and statistical significance for each metabolic pathway; Table S5: pathway enrichment analysis of metabolites in *P. megaterium*, highlighting the matched pathways, key metabolites, and statistical significance for each metabolic pathway; Table S6: enrichment analysis of main-class chemical structures in *B. licheniformis* cocultures with *F. avenaceum*; Table S7: enrichment analysis of main-class chemical structures in *P. megaterium* cocultures with *F. avenaceum.* Table S8: LC–MS data concerning LARAPPI/CI-MSI 2D and LARAPPI/CI-MSI 3D ion images (PDF)
