# Supplementary material for: 3D Mass Spectrometry Imaging as a Novel Screening Method for Evaluating Biocontrol Agents
Source: J Agric Food Chem. 2025 Mar 31;73(14):8225–42. doi: 10.1021/acs.jafc.5c00349 (PMC11987030; doi:10.1021/acs.jafc.5c00349)
Supplement: Supplementary file 1 — jf5c00349_si_001.pdf [file jf5c00349_si_001.pdf]

SUPPORTING INFORMATION

3D Mass Spectrometry Imaging as a Novel Screening Method for Evaluating Biocontrol Agents

Justyna Szulc[1], Tomasz Grzyb[1*], Beata Gutarowska[1], Joanna Nizioł[2], Sumi Krupa[2], Tomasz Ruman[2]

1) Department of Environmental Biotechnology, Faculty of Biotechnology and Food Sciences, Lodz University of Technology, Łódź, Poland
2) Department of Inorganic and Analytical Chemistry, Faculty of Chemistry, Rzeszów University of Technology, Rzeszów, Poland

*Corresponding author: tomasz.grzyb@dokt.p.lodz.pl (T. Grzyb)

Table S1. Compounds identified by LARAPPI/CI-MSI based on acquired LC-MS data and basic metabolites from Ideom database and their representing ion images for microorganisms *Priestia megaterium* and *Fusarium avenaceum*.

**Compound name**	**Ion image**	
Acetone	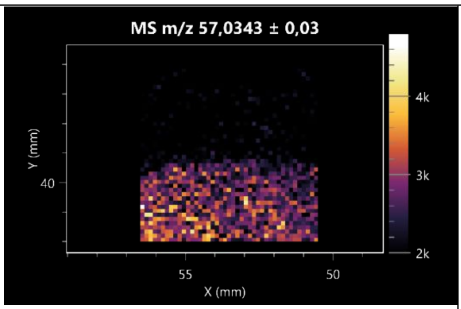	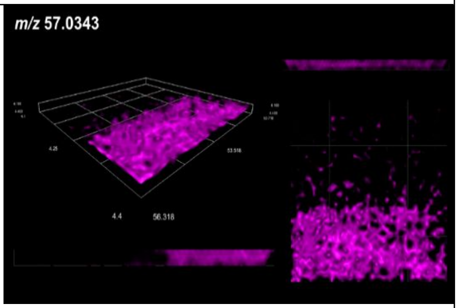
Acetic acid	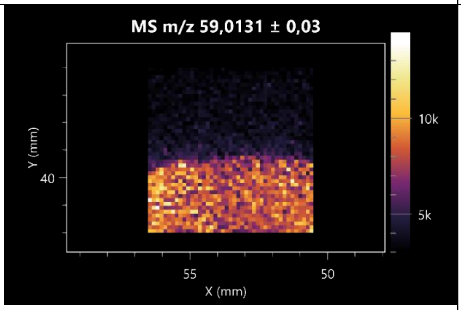	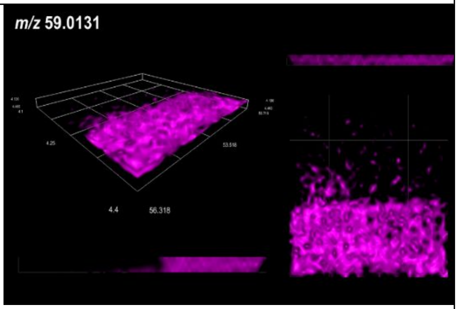
Propionic acid	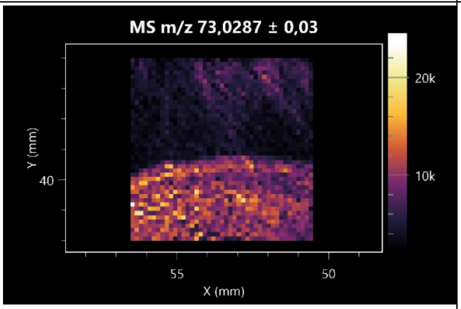	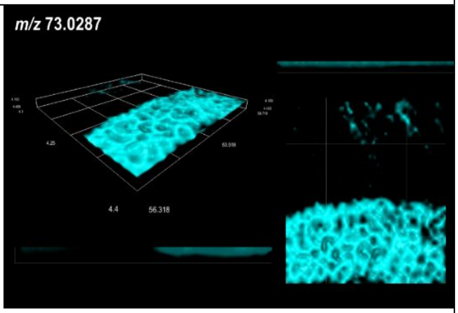
2,3-Butanediol	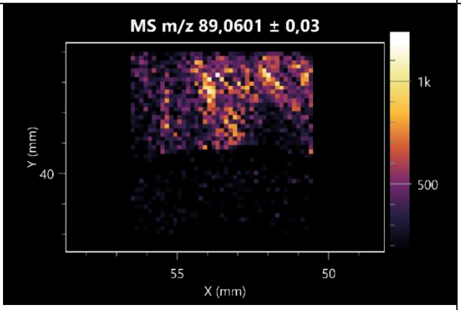	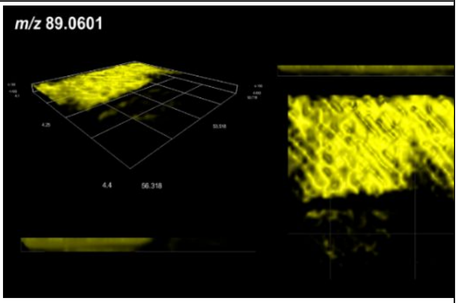

3-Hydroxybutyric acid	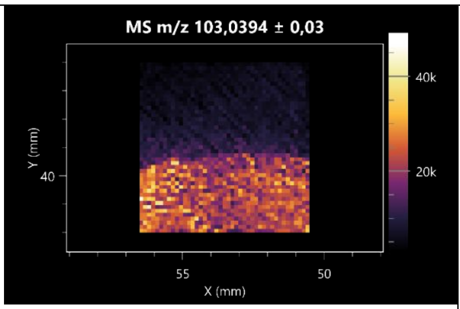	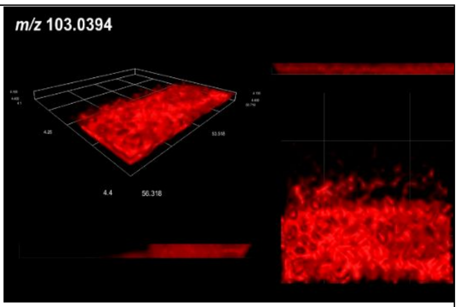
Serine	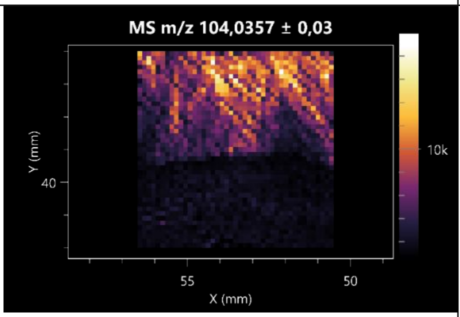	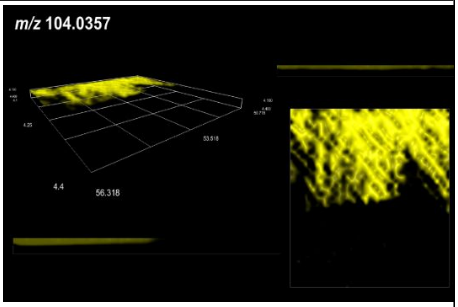
Proline	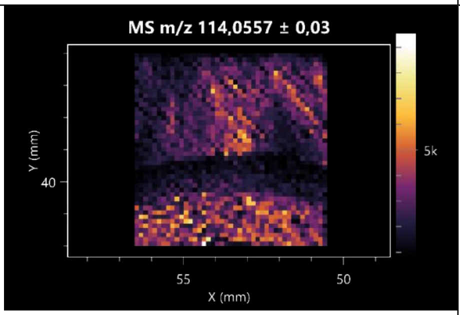	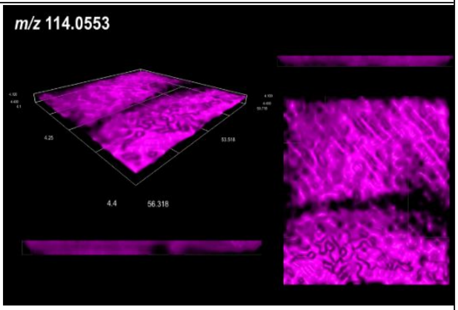
Fumaric acid	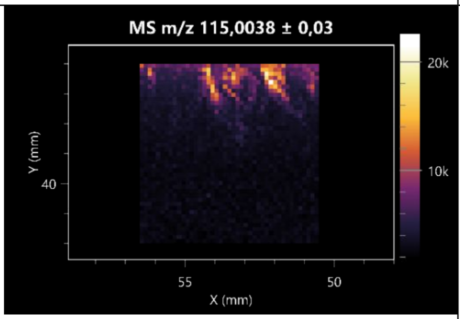	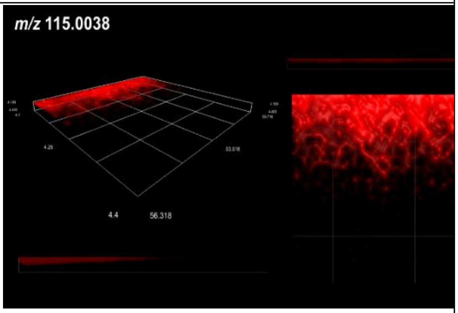
Succinic acid	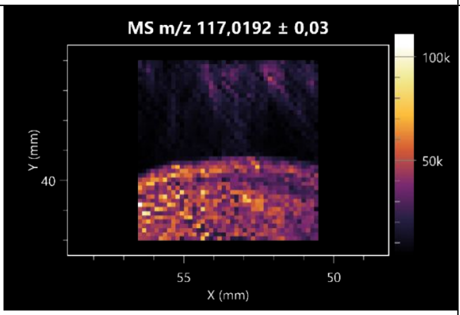	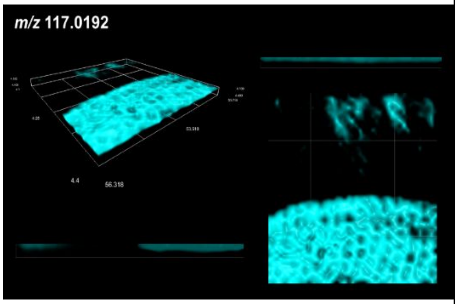

Threonine	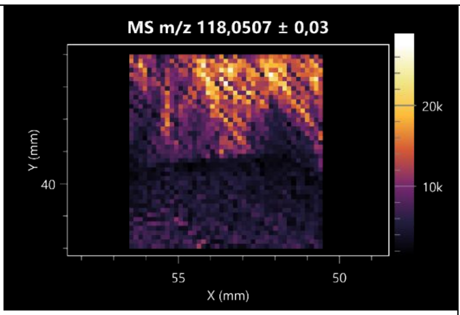	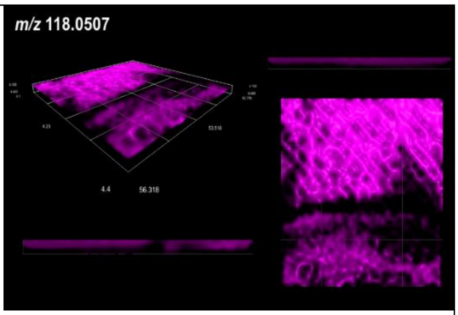
Erythrose	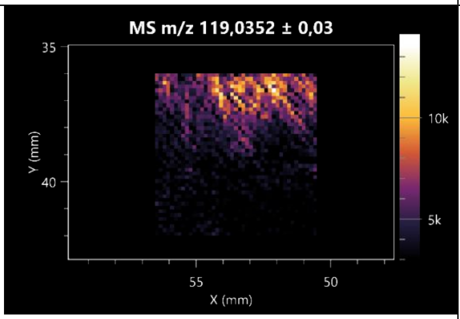	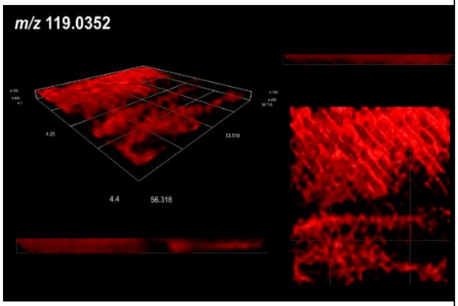
Pyrrolidonecarboxylic acid	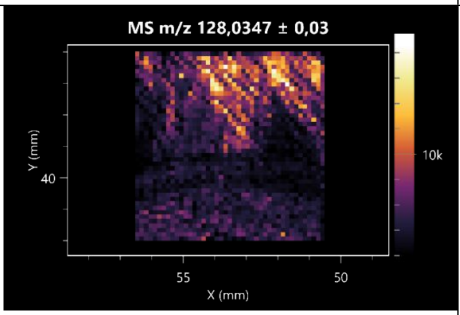	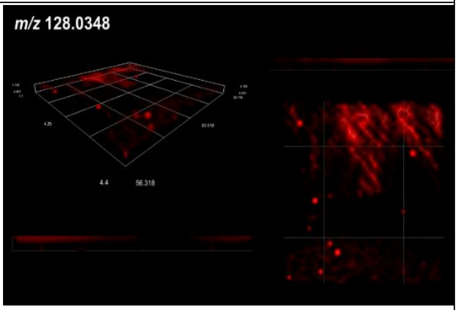
2-Methyl-3-ketovaleric acid	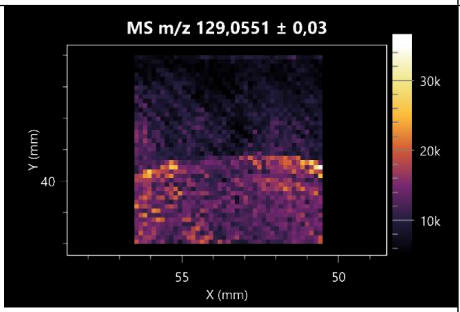	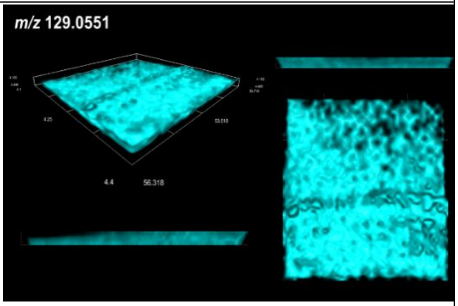
Leucine	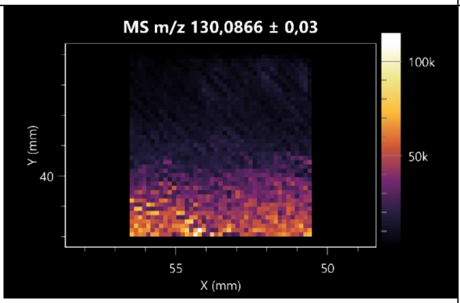	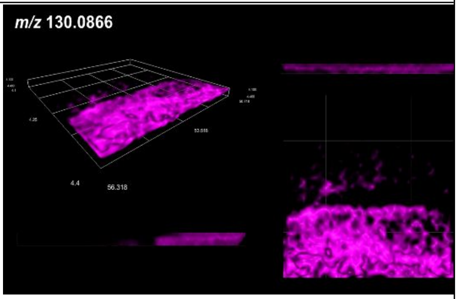

<table>
<tr><td>Methylsuccinic acid</td><td>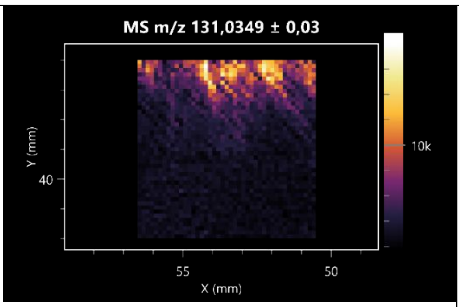
</td><td>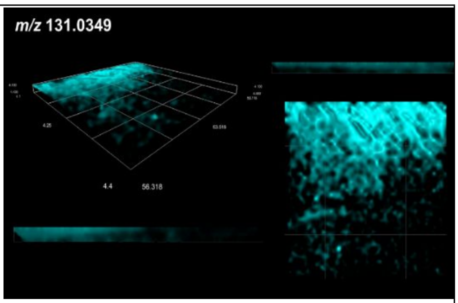
</td></tr>
<tr><td>Aspartic acid</td><td>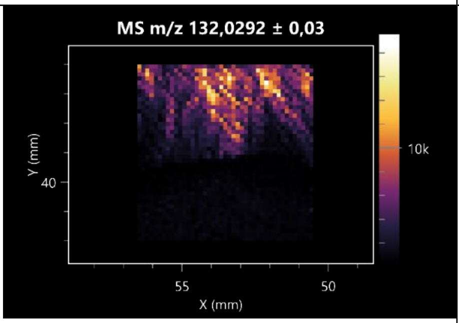
</td><td>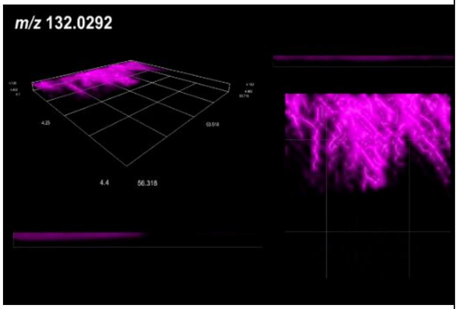
</td></tr>
<tr><td>Malic acid</td><td>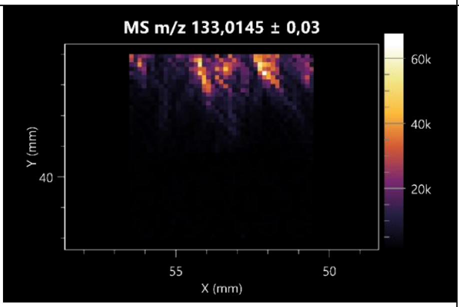
</td><td>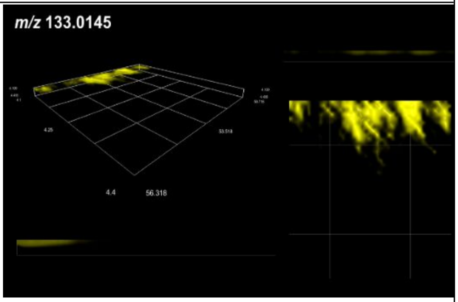
</td></tr>
<tr><td>• 4-Aminobenzoic acid</td><td>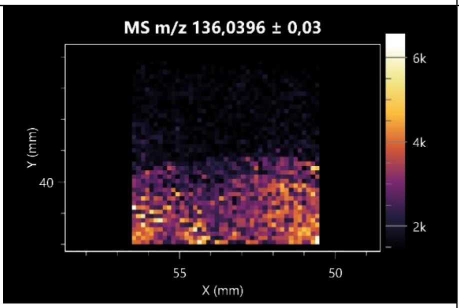
</td><td>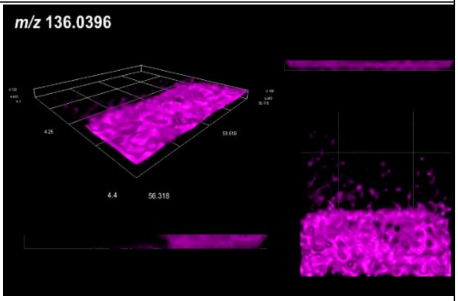
</td></tr>
<tr><td>3-Formylindole</td><td>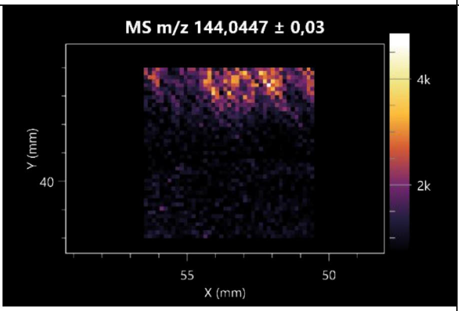
</td><td>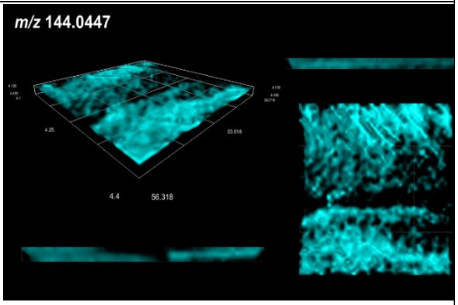
</td></tr>
</table>

Lysine	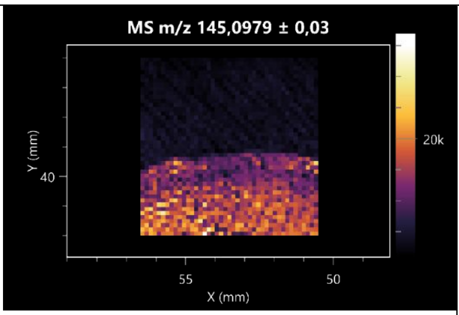	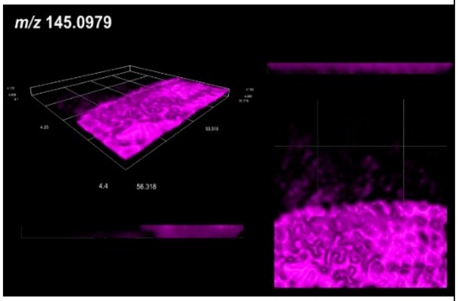
Glutamic acid	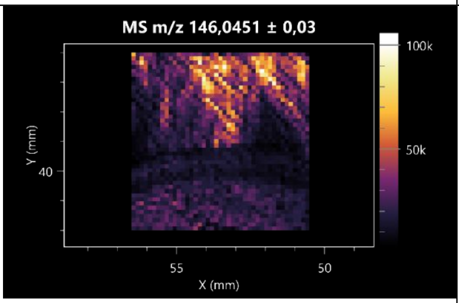	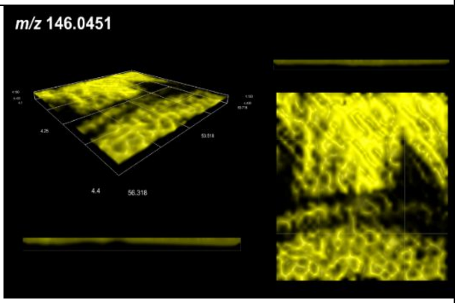
6-(Methylamino) purine	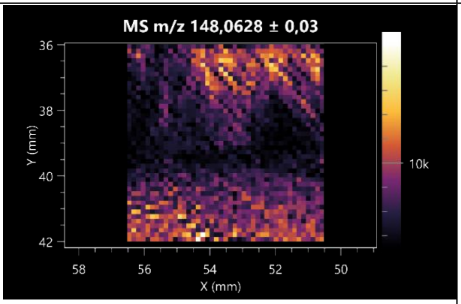	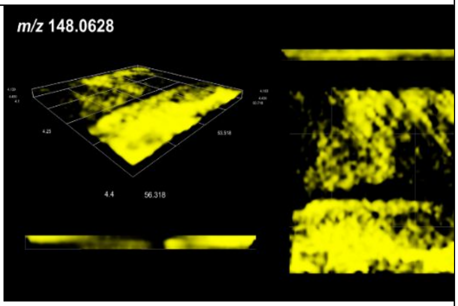
Isoguanine	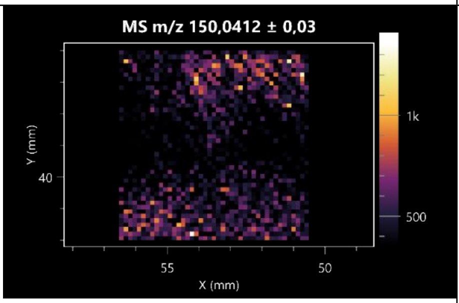	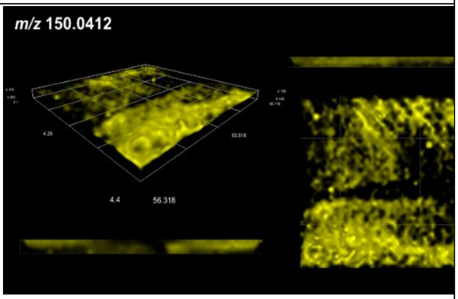
3-Hydroxyphenylacetic acid	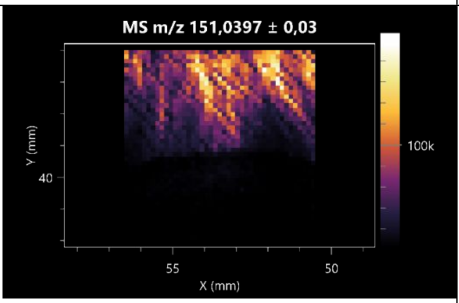	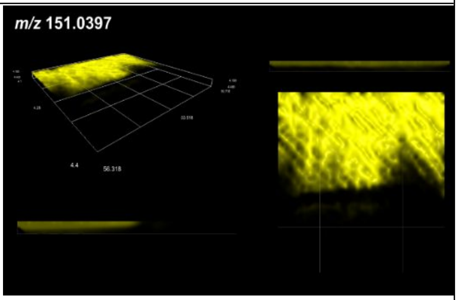

Histidine	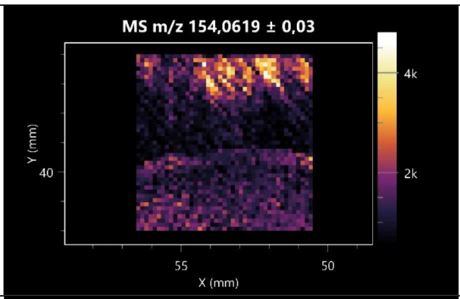	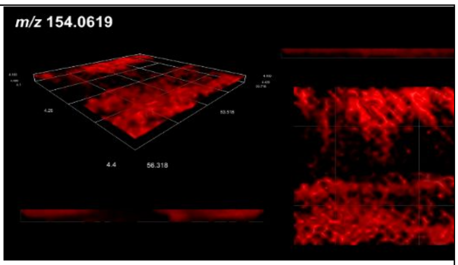
Indole-3-carboxylic acid	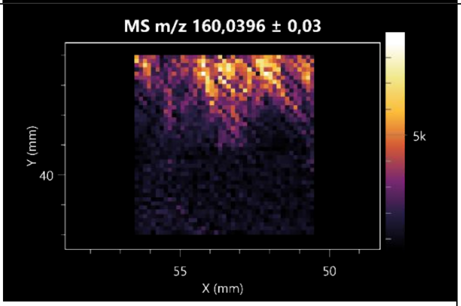	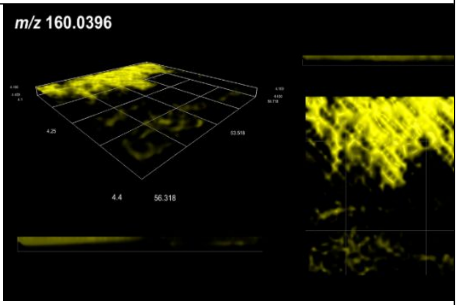
6-Dimethylaminopurine	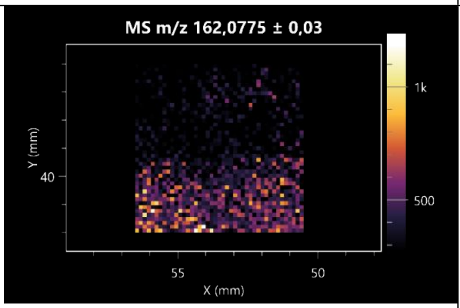	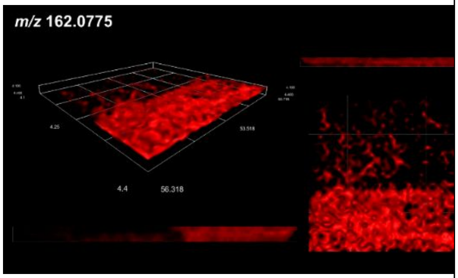
2-Phenylbutyric acid	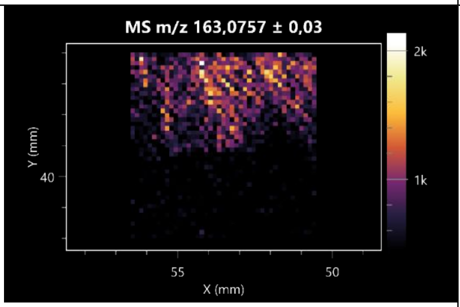	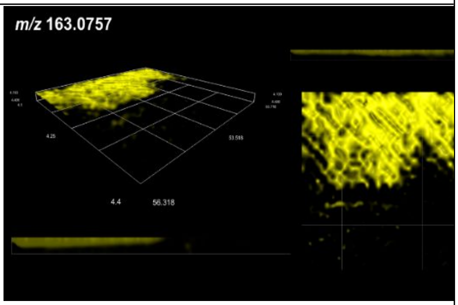
Phenylalanine	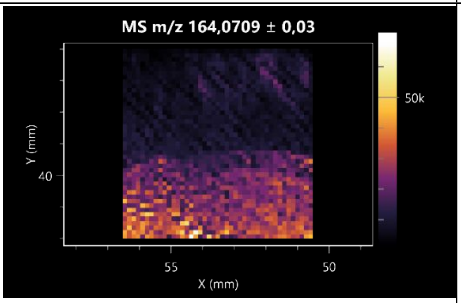	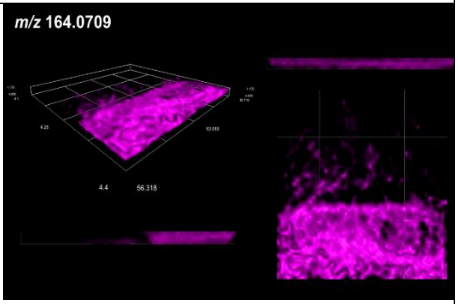

N-α-Acetyl-Ornithine	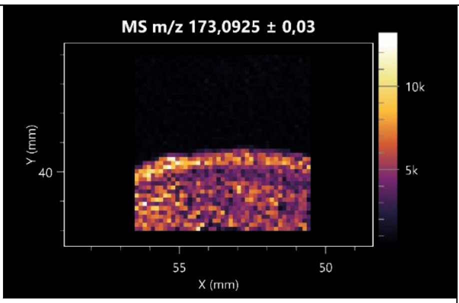	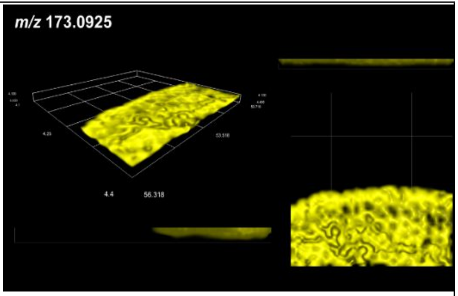
2-Isopropylmalic acid	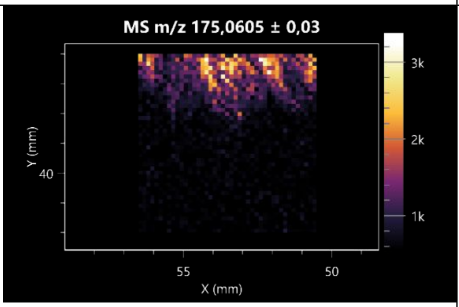	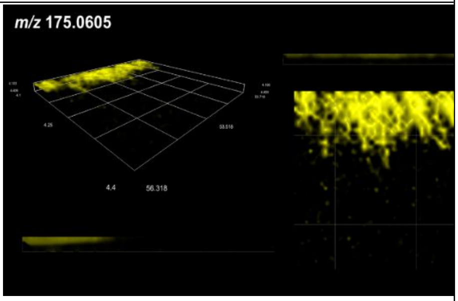
Hippuric acid	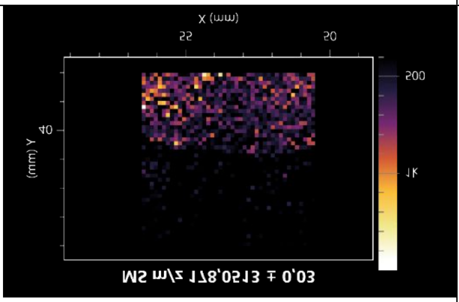	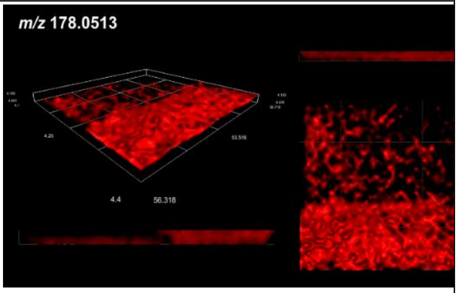
o-Tyrosine	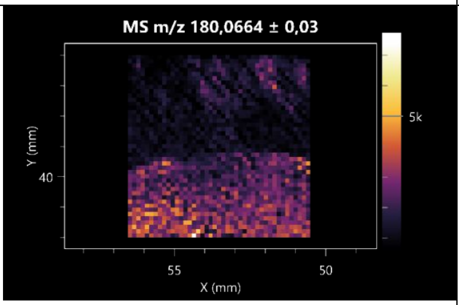	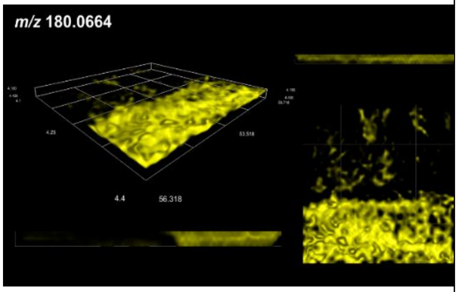
2-Methylnaphtho[2,1-D][1,3]oxazole	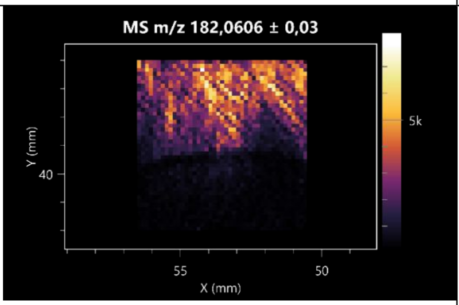	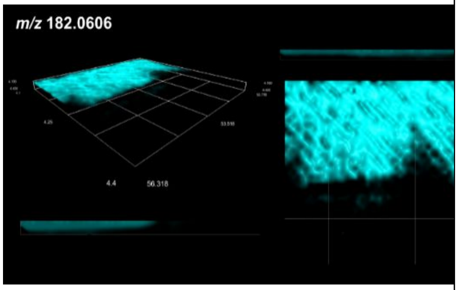

3-Indoleacrylic acid	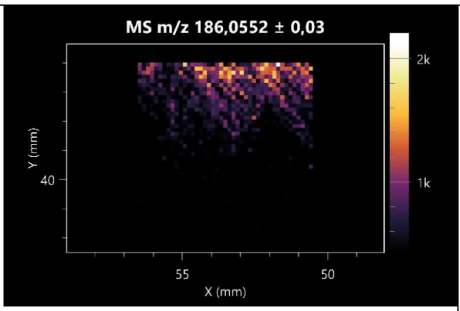	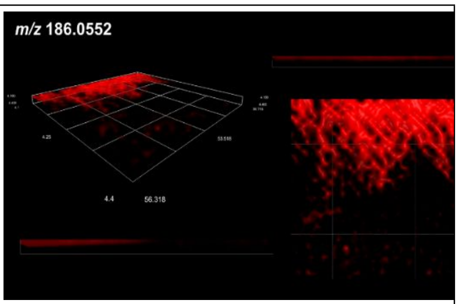
N-α-Acetyllysine	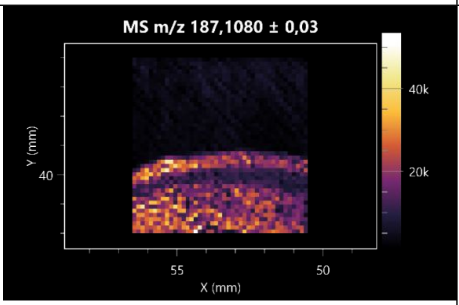	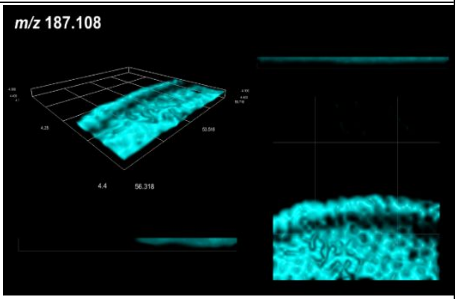
N-Acetylglutamic acid	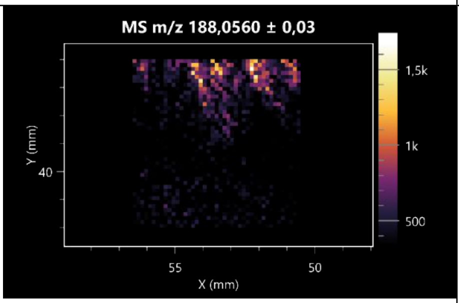	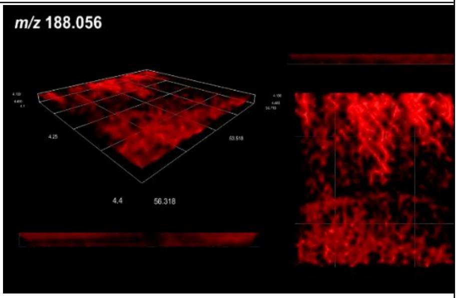
Diaminopimelic acid	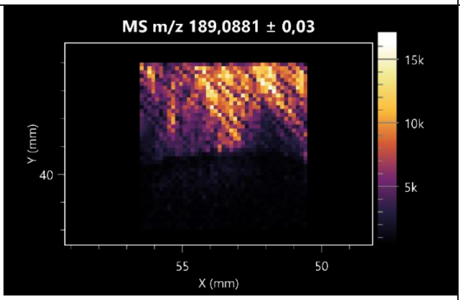	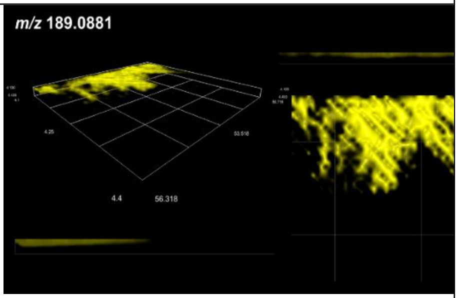
Vanillylmandelic acid	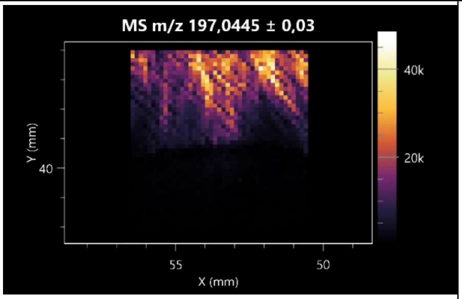	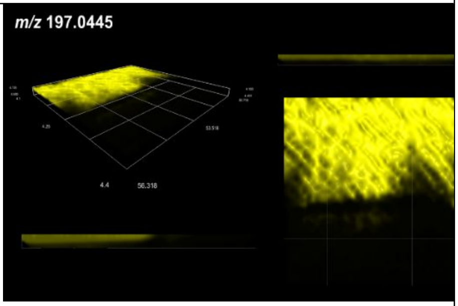

Thr-Pro	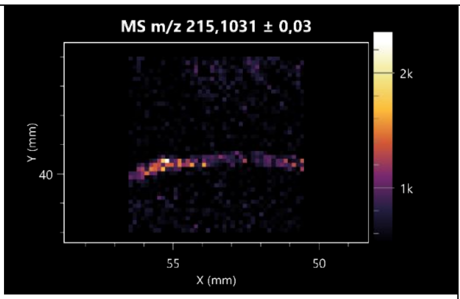	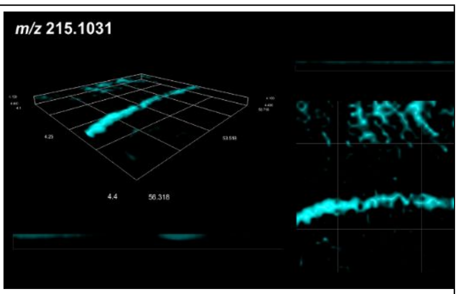
Ser-Ile	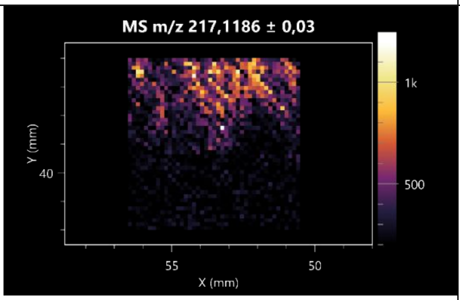	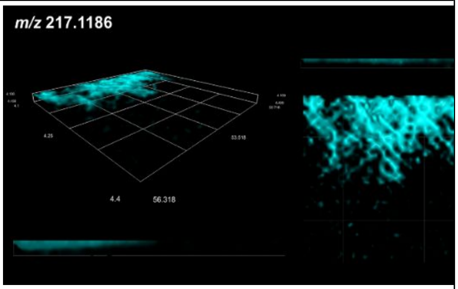
Pantothenic acid	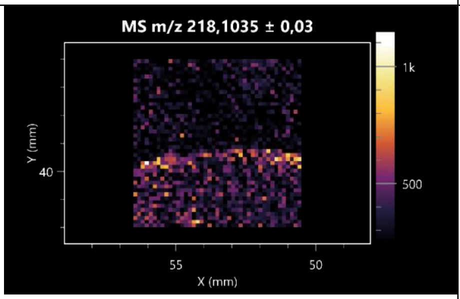	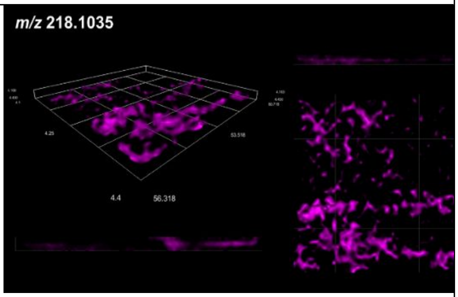
PyroGlu-Val	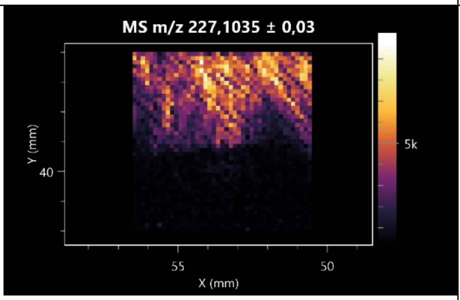	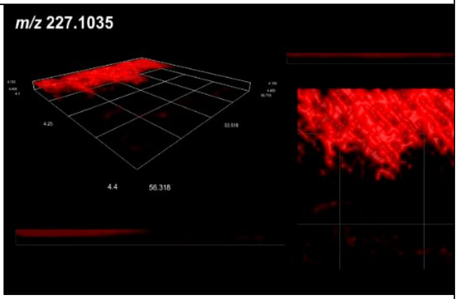
5-Methoxytryptophan	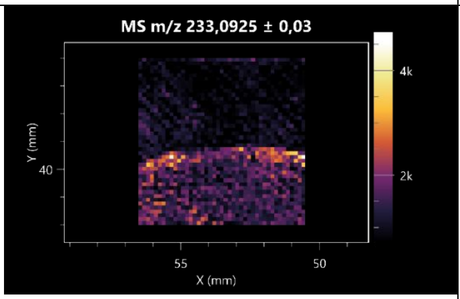	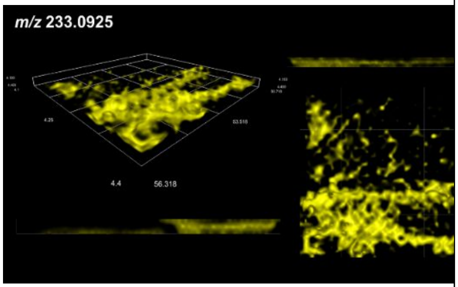

<table>
<tr><td>Val-Lys</td><td>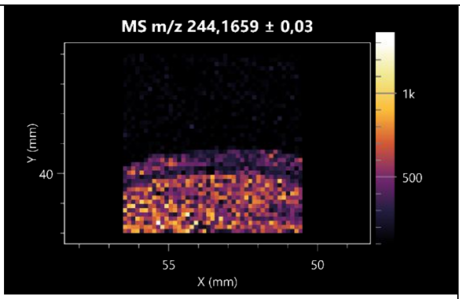
</td><td>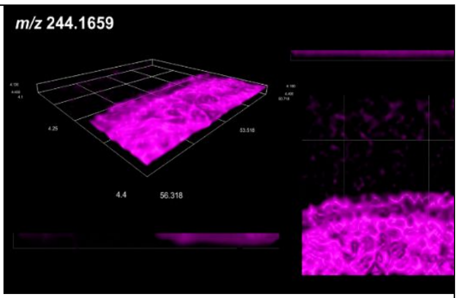
</td></tr>
<tr><td>Pro-Phe</td><td>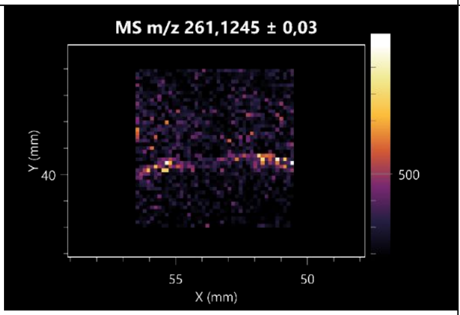
</td><td>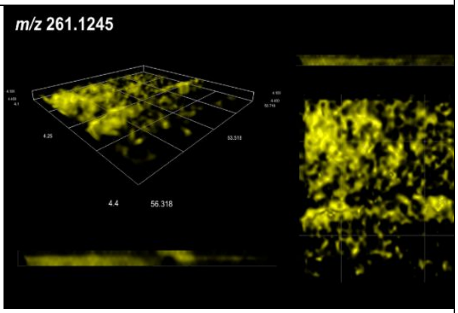
</td></tr>
<tr><td>Linoleic acid</td><td>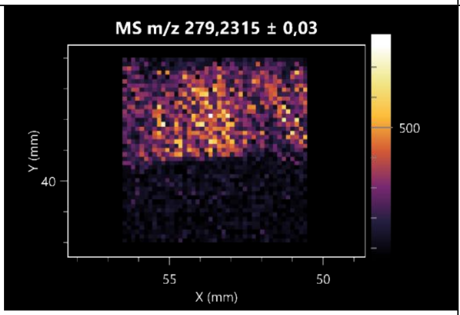
</td><td>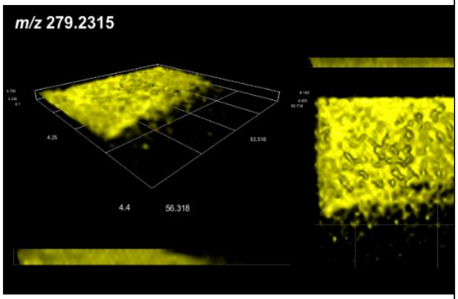
</td></tr>
<tr><td>2-Aceto-2-hydroxybutanoic acid*</td><td>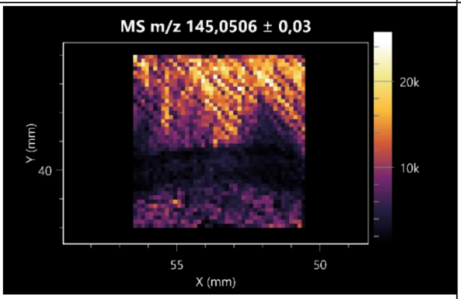
</td><td>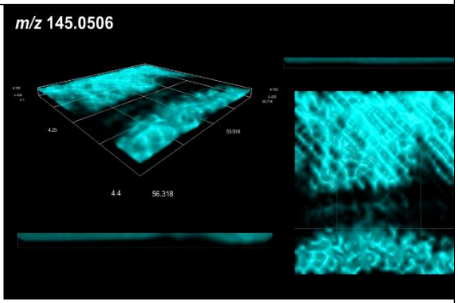
</td></tr>
<tr><td>Dihydroxyfumaric acid*</td><td>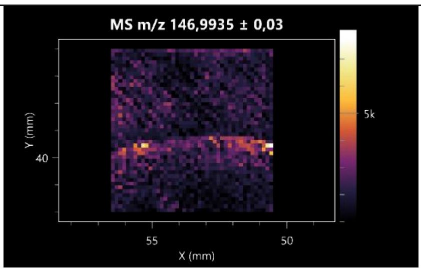
</td><td>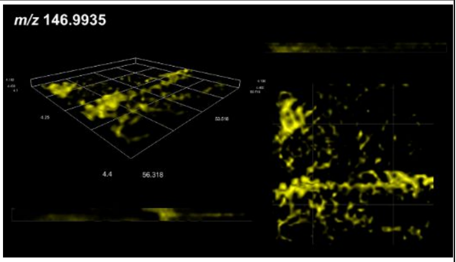
</td></tr>
</table>

* Compounds identified based on Ideom database, not previously identified using LC-MS data

Table S2. Compounds identified by LARAPPI/CI-MSI based on acquired LC-MS data and basic metabolites from Ideom database and their representing ion images for microorganisms *Bacillus licheniformis* and *Fusarium avenaceum*

**Compound name**	**Ion image**	
2,3-Butanediol	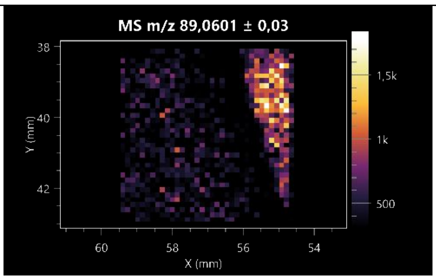	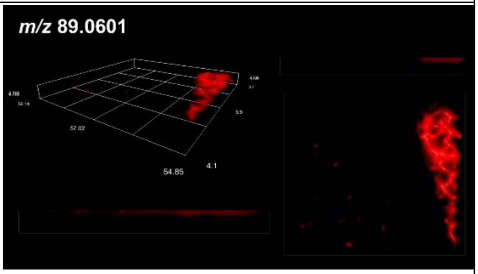
D-Alpha-aminobutyric acid	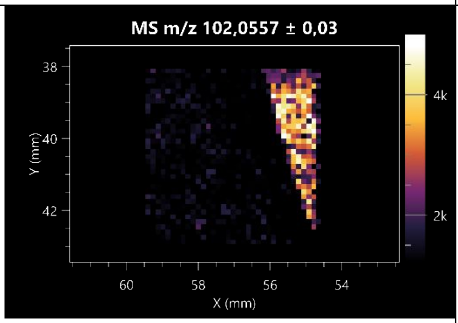	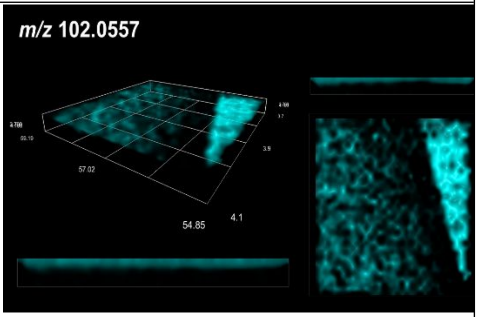
L-Serine	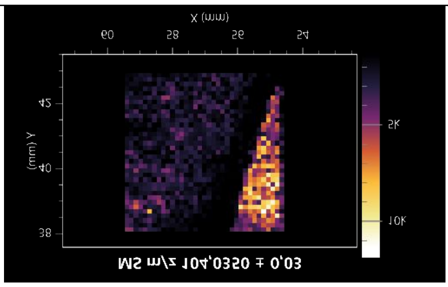	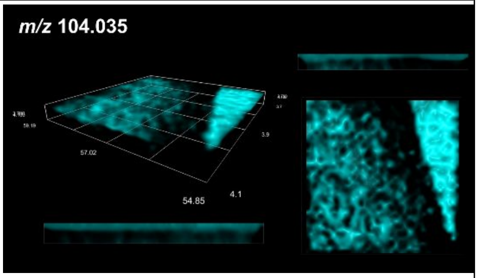
Uracil	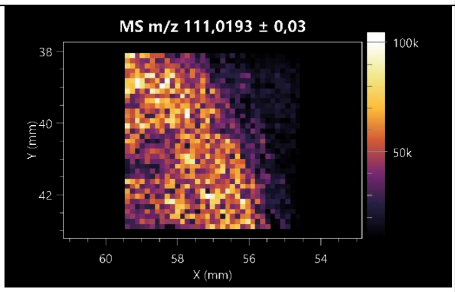	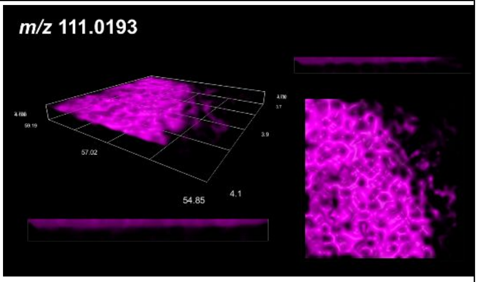

Betaine	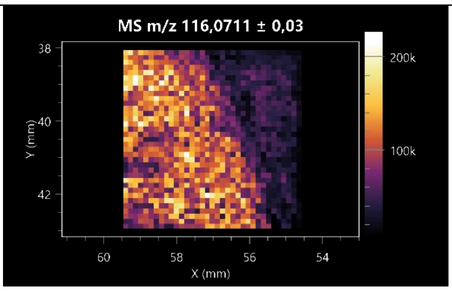	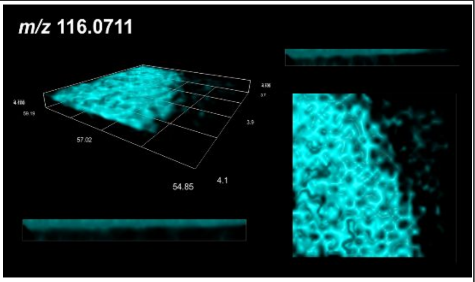
Erythrose	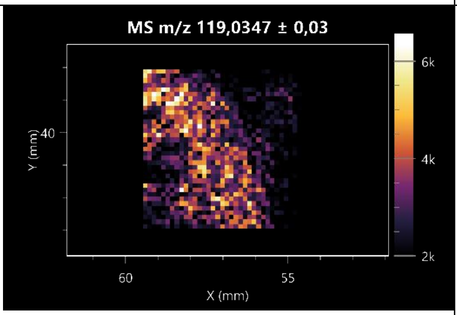	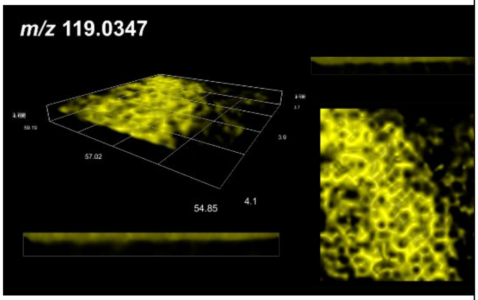
Pipecolic acid	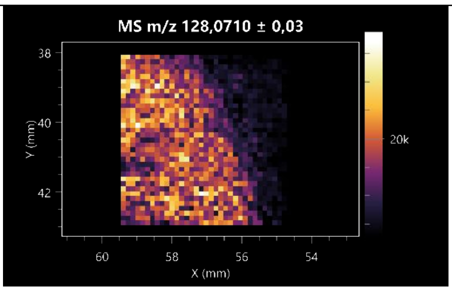	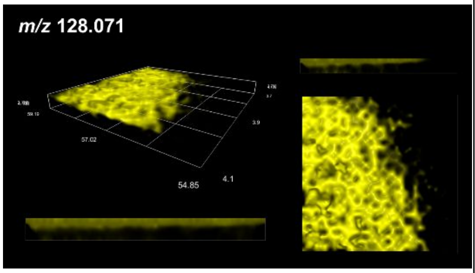
2-Methylindole	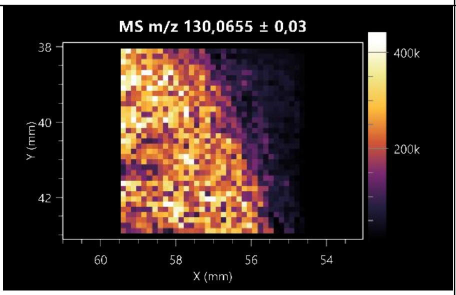	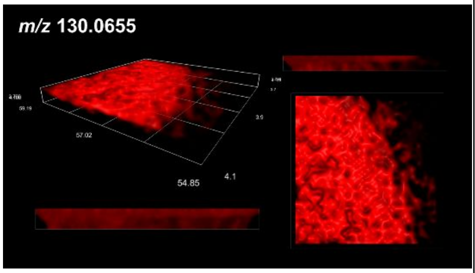
2-Hydroxycaproic acid	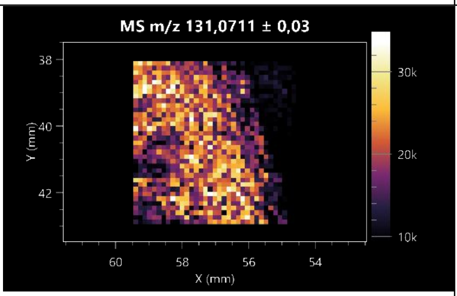	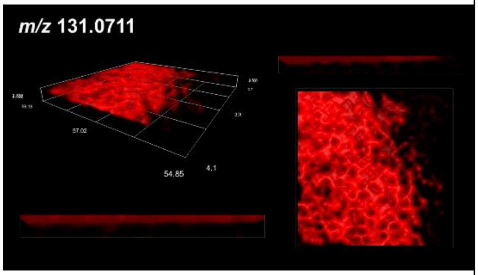

L-Aspartic acid	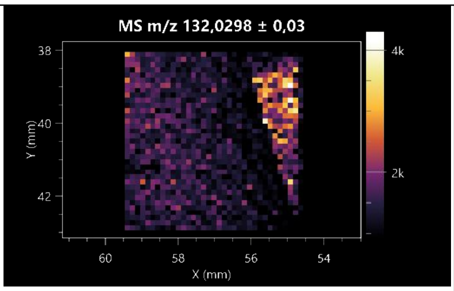	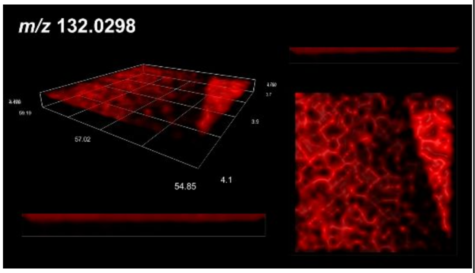
4-Acetamidobutanoate	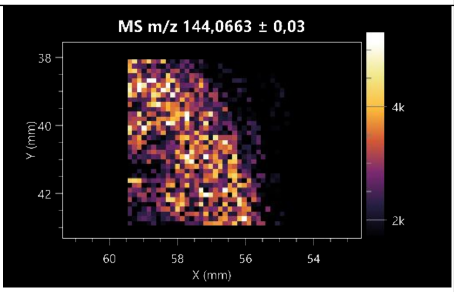	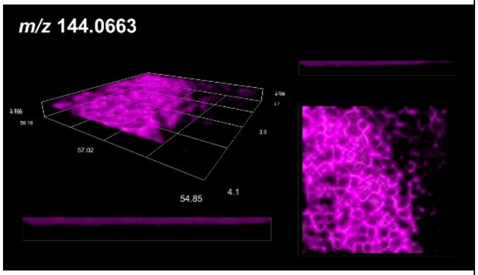
Lysine	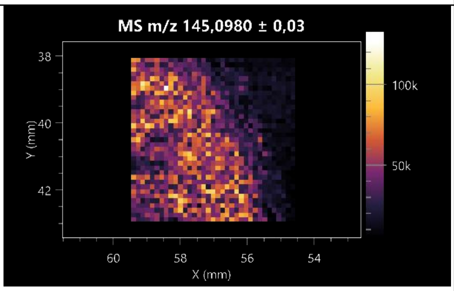	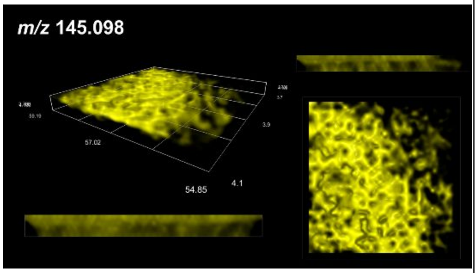
Glutamic acid	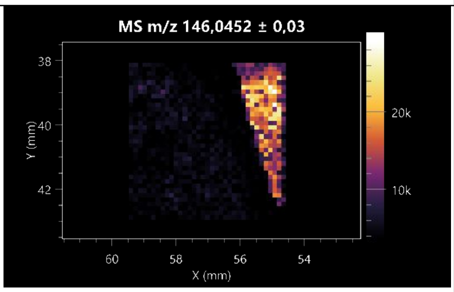	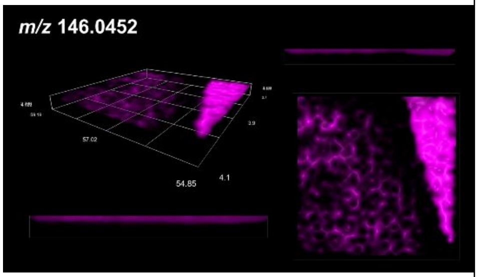
Trans-Cinnamic acid	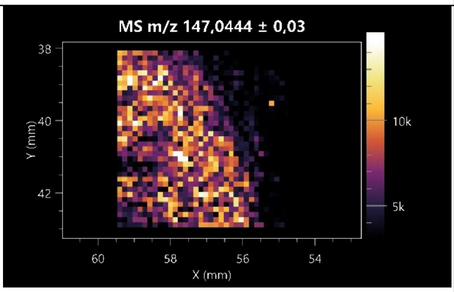	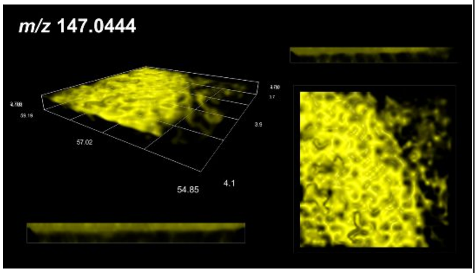

Methionine	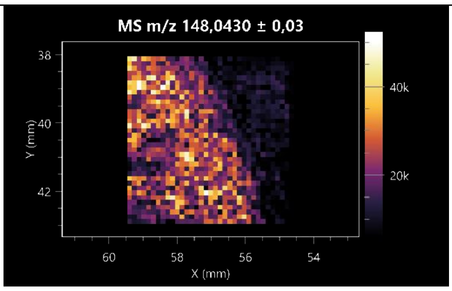	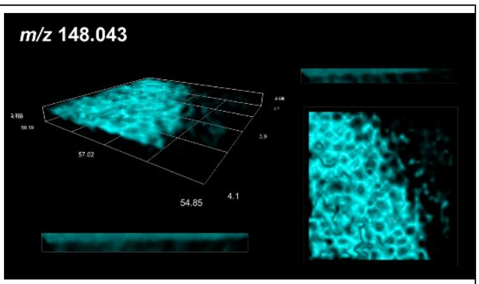
4-Ethylbenzoic acid	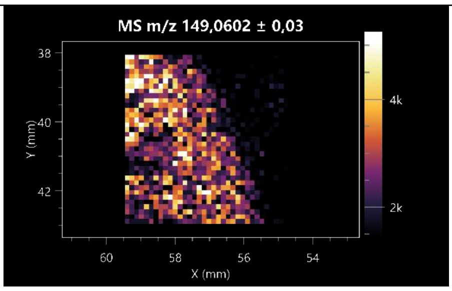	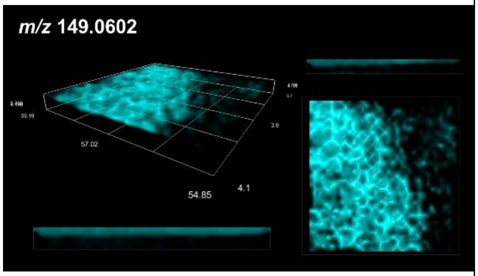
Acetaminophen	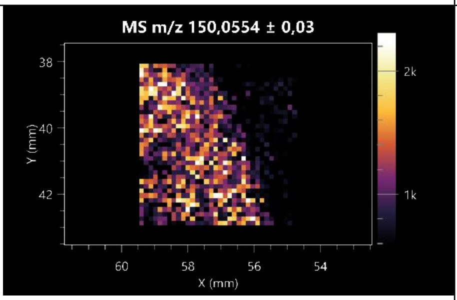	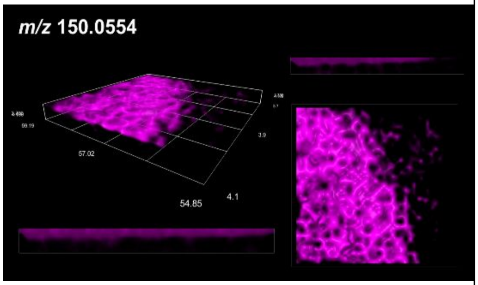
Histidine	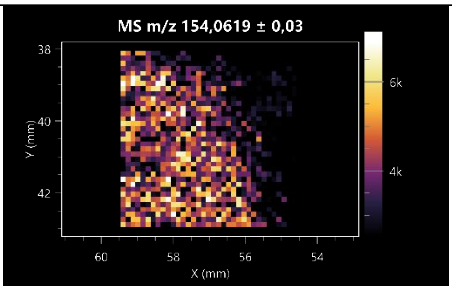	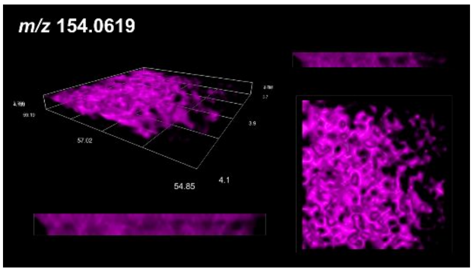
Isovalerylglycine	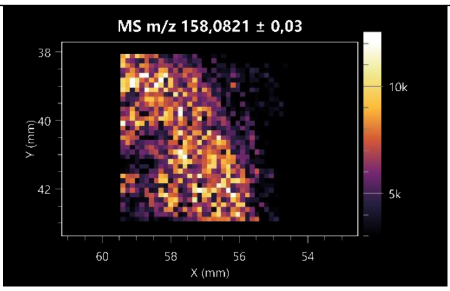	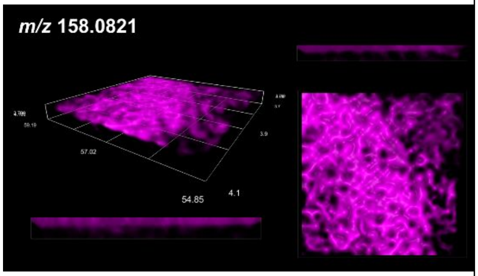

6-Dimethylaminopurine	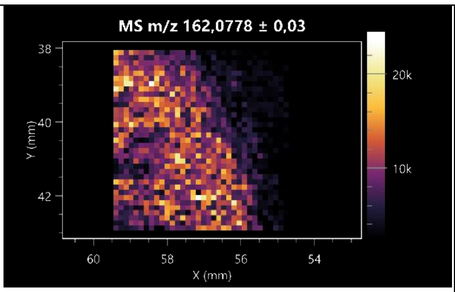	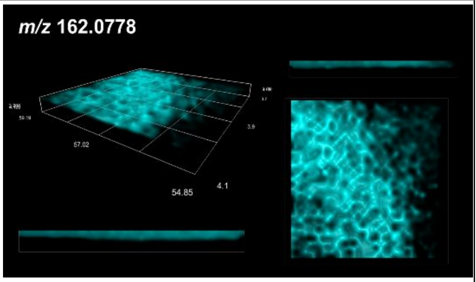
Phenylalanine	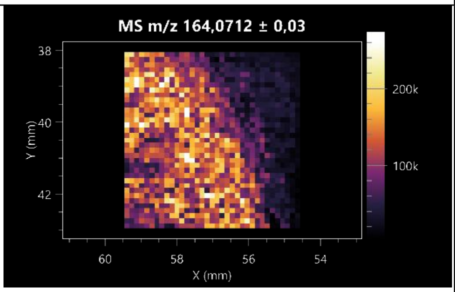	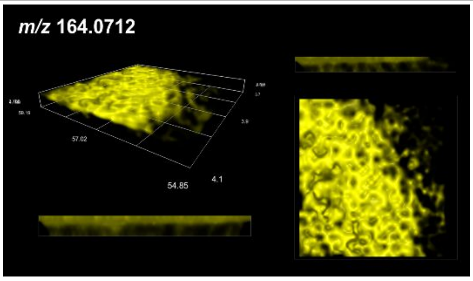
Tyrosine	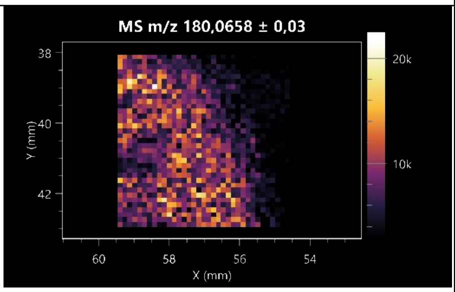	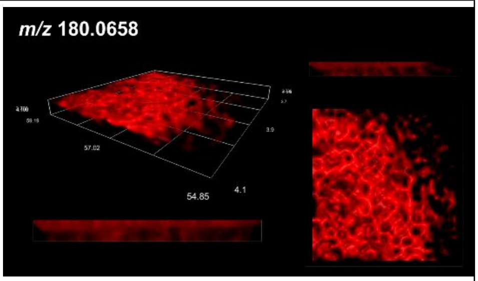
Sorbitol	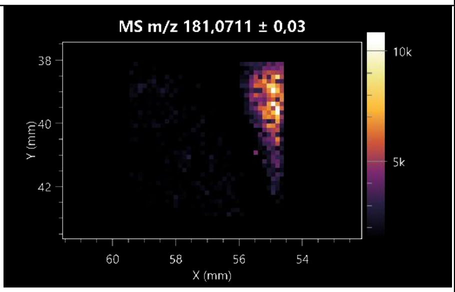	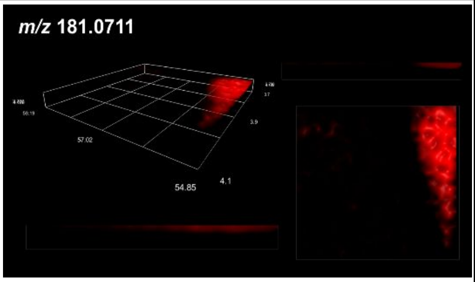
Diaminopimelic acid	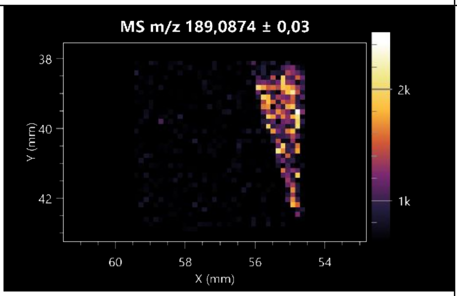	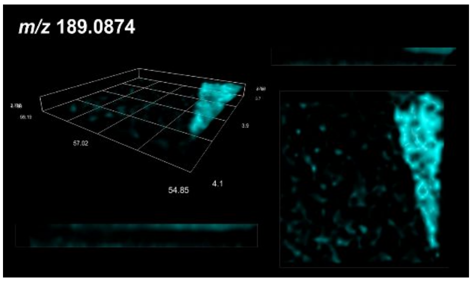

N-[2-(1H-Indol-3-yl)ethyl]acetamide	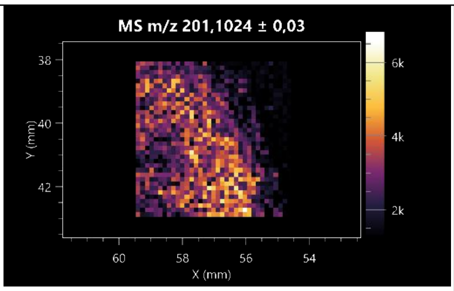	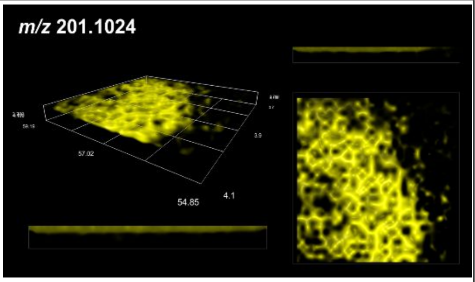
N-Acetylmannosamine	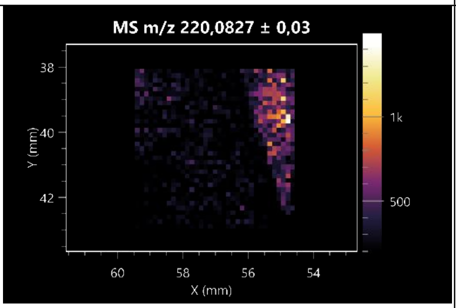	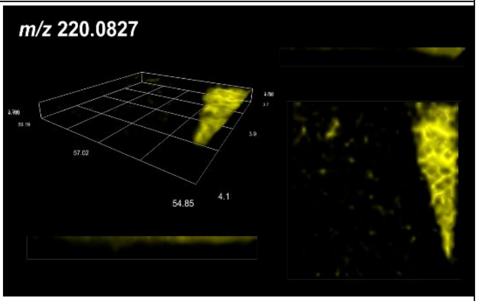
Leu-Pro	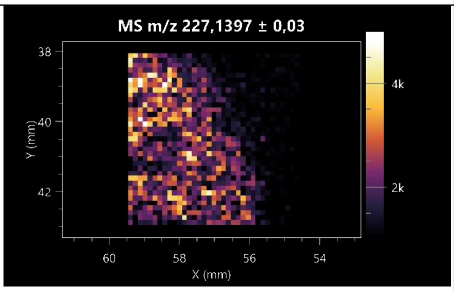	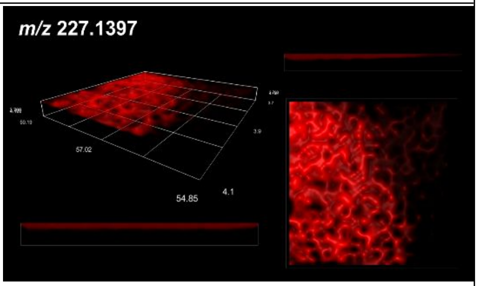
Ser-Glu	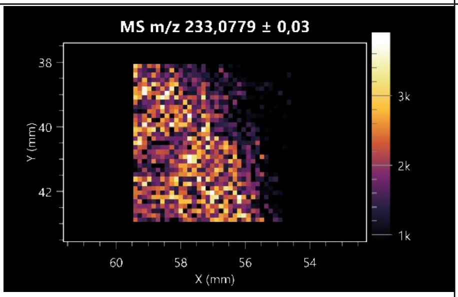	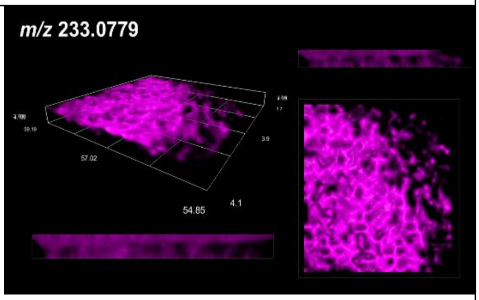
Methyl myristoleate	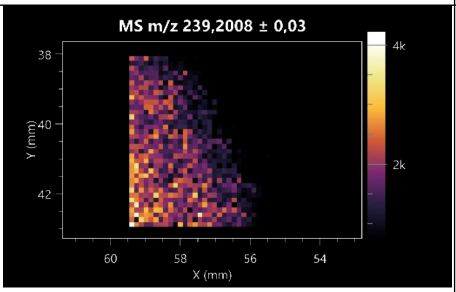	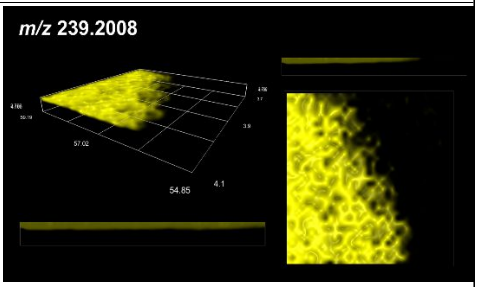

Pentadecanoic acid	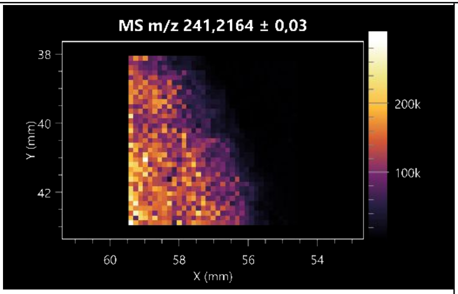	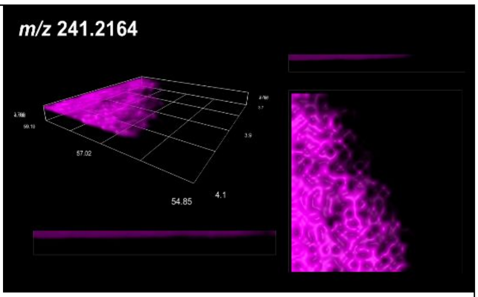
γ-Glutamylleucine	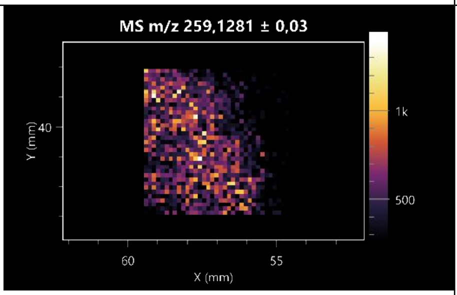	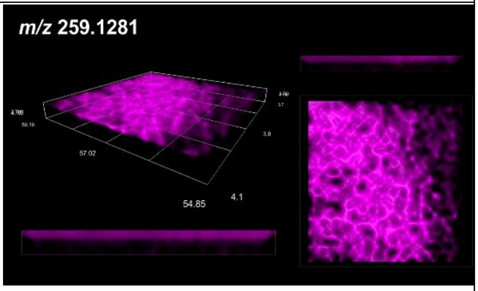
Monolaurin	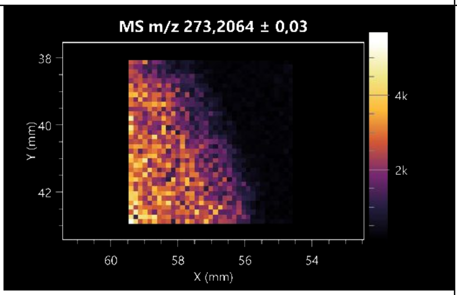	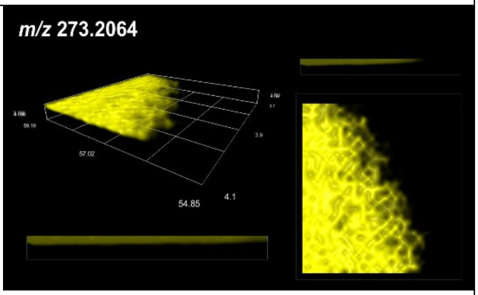
Linolenic acid	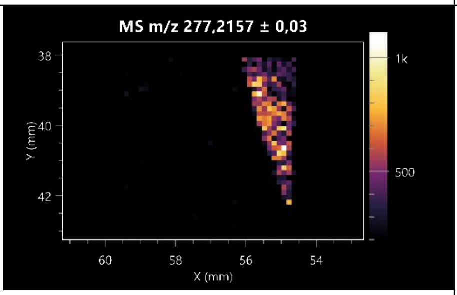	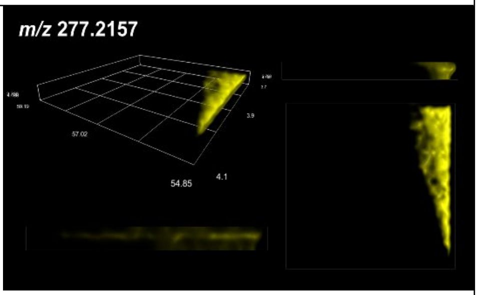
Linoleic acid	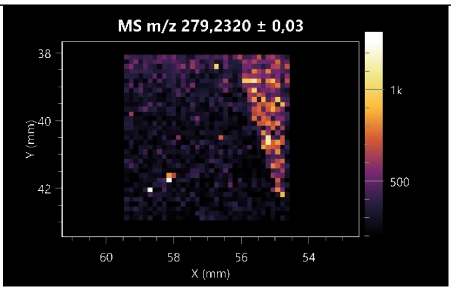	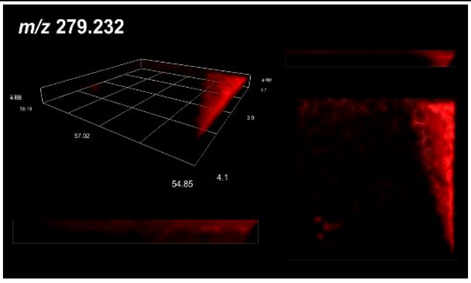

Nonadecanoic acid	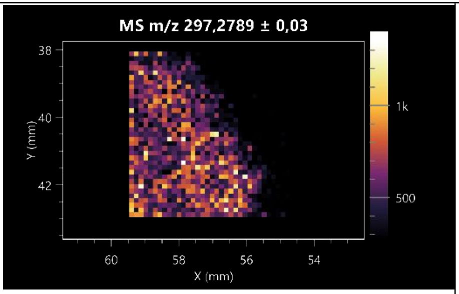	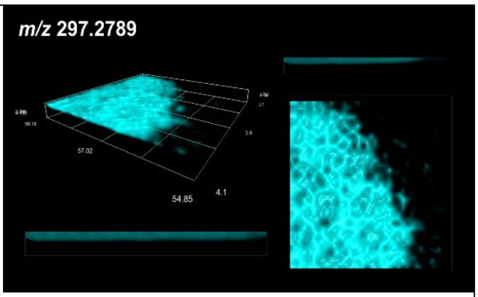
Glycerol-myristate	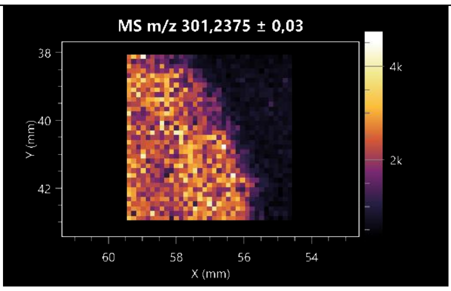	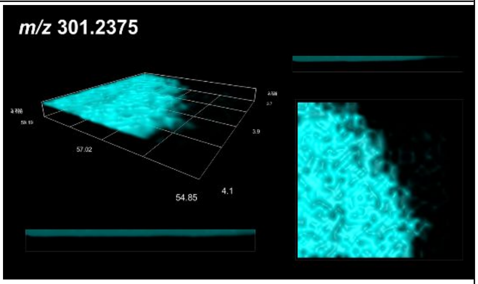
Alanine*	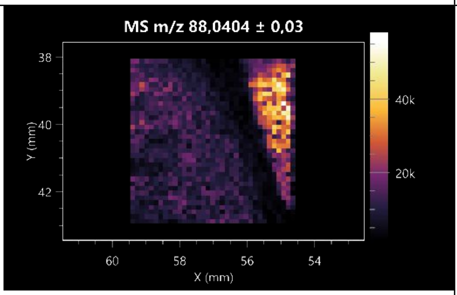	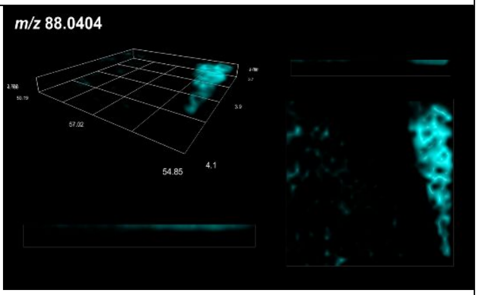
Choline*	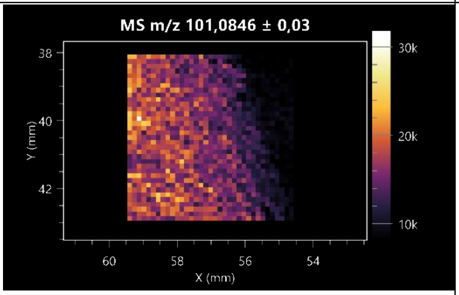	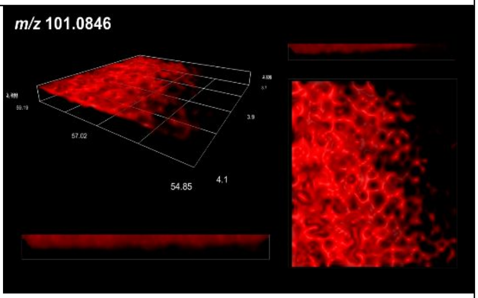
5-Hydroxypentanoic acid*	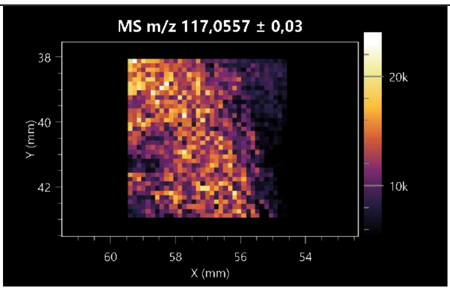	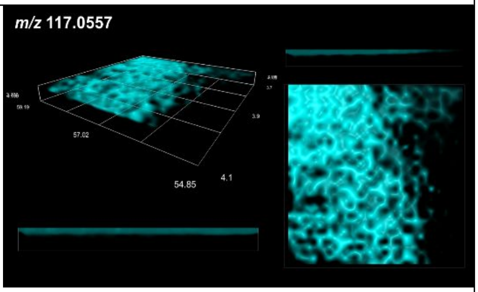

Deoxyribose*	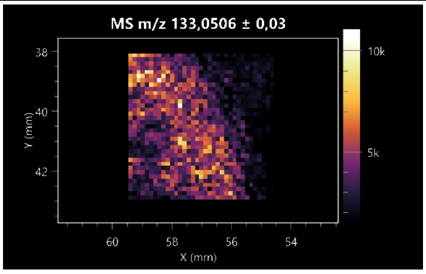	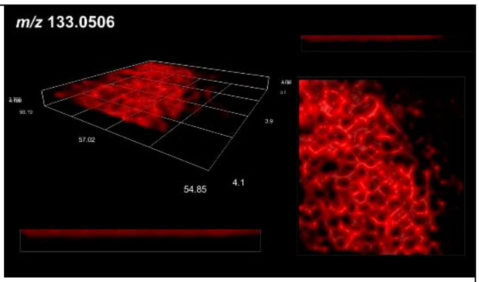
2,6-Diaminopurine*	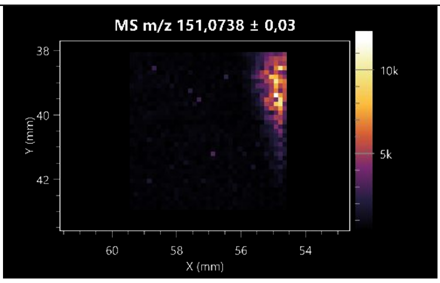	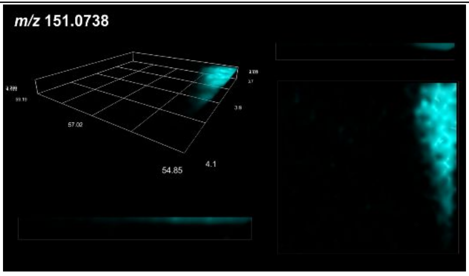
3-Deoxy-D-galactose*	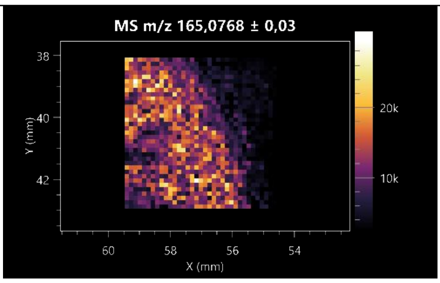	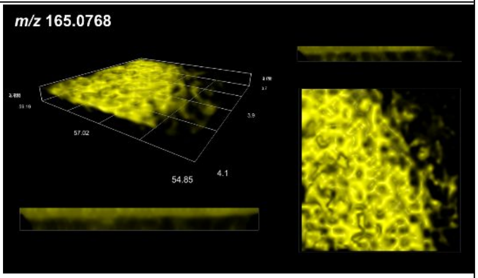
Tetradecenol*	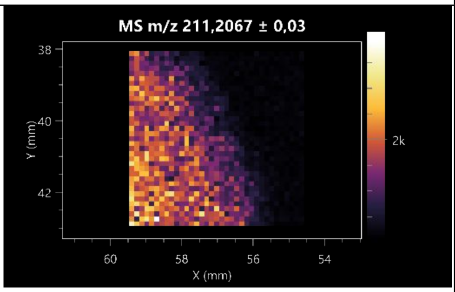	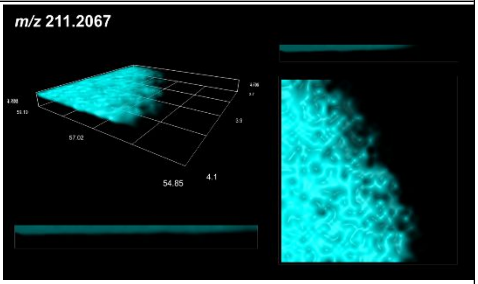

* Compounds identified based on Ideom database, not previously identified using LC-MS data

Table S3. Key metabolites discriminating between *Bacillus licheniformis* and *Priestia megaterium* cocultures with *Fusarium avenaceum*. A fold change greater than 2 indicates a significantly higher abundance of the metabolite in *B. licheniformis*, a fold change below 0.5 indicates a significantly higher abundance in *P. megaterium*

No	Name	Formula	$m/z_{exp.}$	RT [s.]	Ion mode	VIP	P-value	Fold Change
1	3-Hydroxyhippuric acid	$C_9H_9NO_4$	195.0535	389.1	NEG	1.291	3.89E-16	0.031
2	Glycerophosphocholine	$C_8H_{20}NO_6P$	295.0584	62.3	POS	1.291	1.31E-17	0.200
3	PC(36:3)	$C_{44}H_{82}NO_8P$	783.5758	1004.5	POS	1.291	1.52E-16	5.016
4	LPC(16:0)	$C_{24}H_{50}NO_7P$	541.3381	920.5	NEG	1.291	1.47E-13	0.198
5	3-Indoleacrylic acid	$C_{11}H_9NO_2$	187.0630	361.9	POS	1.291	3.41E-17	5.542
6	LPE 18:2	$C_{23}H_{44}NO_7P$	477.2857	835.6	NEG	1.291	3.28E-16	0.199

7	Senecioic acid	$C_5H_8O_2$	141.0790	488.7	POS	1.291	8.86E-15	0.199
8	(2-Amino-1-phenylethyl)dimethylamine	$C_{10}H_{16}N_2$	164.1311	341.3	POS	1.291	2.55E-12	0.197
9	Uridine	$C_9H_{12}N_2O_6$	244.0695	364.8	POS	1.291	1.04E-12	4.627
10	Levoglucosan	$C_6H_{10}O_5$	203.0790	134.4	POS	1.291	3.02E-12	0.197
11	Isopropyl alcohol	C_3H_8O	101.0839	1233.3	POS	1.291	3.40E-13	5.059
12	Butyryl-carnitine	$C_{11}H_{21}NO_4$	231.1469	338.3	POS	1.291	3.20E-12	0.197
13	*N*-acetyltryptophan	$C_{13}H_{14}N_2O_3$	246.1009	484.2	NEG	1.291	1.09E-11	0.283
14	*N*-Isobutyl-2-(2-methyl-1*H*-indol-1-yl)acetamide	$C_{15}H_{20}N_2O$	244.1574	655.9	POS	1.291	6.39E-12	54.731
15	Succinic acid semialdehyde	$C_4H_6O_3$	119.0580	396.5	POS	1.291	7.95E-13	0.197
16	Ile-Pro-Ile	$C_{17}H_{31}N_3O_4$	341.2318	449.0	POS	1.291	1.64E-12	0.123
17	Levulinic acid	$C_5H_8O_3$	138.0296	255.1	POS	1.291	2.32E-11	0.195
18	Glycyl-L-leucine	$C_8H_{16}N_2O_3$	188.1159	324.7	POS	1.291	2.83E-11	8.152
19	Pantothenic acid	$C_9H_{17}NO_5$	219.1116	369.8	POS	1.291	3.87E-11	8.865
20	Indoleacetic acid	$C_{10}H_9NO_2$	175.0630	529.4	POS	1.291	4.33E-12	5.540
21	PC(O-16:1)	$C_{24}H_{48}NO_7P$	493.3162	814.3	POS	1.291	5.77E-11	0.194
22	2-Isopropylmalic acid	$C_7H_{12}O_5$	217.0947	376.6	POS	1.291	8.82E-12	0.191
23	L-3-Phenyllactic acid	$C_9H_{10}O_3$	166.0633	470.8	NEG	1.291	5.37E-11	0.263
24	4,12,12-trimethyl-9-methylene-5-oxatricyclo[8.2.0.0{4,6}]dodecane	$C_{15}H_{24}O$	220.1825	622.4	POS	1.291	7.65E-11	0.194
25	*N*,*N*-Diethylcathinone	$C_{13}H_{19}NO$	205.1461	664.1	POS	1.291	1.06E-11	17.312
26	*N*-[2-(1*H*-Indol-3-yl)ethyl]acetamide	$C_{12}H_{14}N_2O$	202.1104	526.9	POS	1.291	2.17E-11	101.100
27	Glu-Trp	$C_{16}H_{19}N_3O_5$	333.1319	412.1	POS	1.291	6.60E-11	5.145
28	2-Hydroxycaproic acid	$C_6H_{12}O_3$	132.0788	435.6	NEG	1.290	8.56E-11	5.152
29	LPE(18:1)	$C_{23}H_{46}NO_7P$	479.3016	902.2	NEG	1.290	2.14E-10	0.193
30	LPE(18:3)	$C_{23}H_{42}NO_7P$	475.2692	800.2	POS	1.290	2.22E-10	0.193
31	2-Methylindole	C_9H_9N	131.0733	362.0	POS	1.290	3.30E-10	5.324
32	Hexa-2,4-dienoic acid	$C_6H_8O_2$	112.0522	252.1	POS	1.290	4.37E-10	0.192
33	Indole-3-carboxylic acid	$C_9H_7NO_2$	161.0479	500.5	NEG	1.290	1.08E-10	0.229
34	Dimethylprotoporphyrin IX dimethyl ester	$C_{36}H_{38}N_4O_4$	631.3158	357.8	POS	1.290	1.17E-10	0.194
35	1-palmitoyl-2-hydroxy-sn-glycero-3-phosphoethanolamine	$C_{21}H_{44}NO_7P$	453.2851	864.6	POS	1.290	2.77E-10	5.187
36	Methionine	$C_5H_{11}NO_2S$	149.0508	359.0	POS	1.290	6.03E-10	0.192
37	Pyruvaldehyde	$C_3H_4O_2$	113.0476	43.9	POS	1.290	6.25E-10	0.192
38	(5E)-Heptadeca-5,16-diene-1,2,4-triol	$C_{17}H_{32}O_3$	284.2349	916.6	POS	1.290	5.92E-10	0.180
39	2-Hydroxy-5 (6)Epoxy-Tetrahydrocaryophyllene	$C_{15}H_{26}O_2$	238.1930	657.0	POS	1.290	6.43E-10	0.094
40	4-Deoxytetronic acid	$C_4H_6O_2$	86.0367	148.9	POS	1.290	7.54E-10	0.191
41	4-Methyl-.alpha.-ethylaminopentiophenone	$C_{14}H_{21}NO$	219.1621	727.9	POS	1.290	3.84E-10	5.198

42	Diprotin B	$C_{16}H_{29}N_3O_4$	327.2159	405.1	POS	1.290	8.66E-10	0.099
43	Phytanic acid	$C_{20}H_{40}O_2$	375.3107	1003.4	POS	1.290	3.15E-10	2.489
44	PE(30:0)	$C_{35}H_{70}NO_8P$	663.4828	988.2	POS	1.290	4.69E-10	5.205
45	Cytidine monophosphate	$C_9H_{14}N_3O_8P$	361.0072	69.4	POS	1.290	2.62E-10	0.193
46	LPG(15:0)	$C_{21}H_{43}O_9P$	470.2647	879.9	NEG	1.290	3.97E-10	0.066
47	Trp-Ile	$C_{17}H_{23}N_3O_3$	317.1735	456.1	POS	1.290	6.05E-10	3.930
48	Phosphonoacetate	$C_2H_5O_5P$	139.9875	76.8	POS	1.290	1.14E-09	0.499
49	Sulfolithocholic acid	$C_{24}H_{40}O_6S$	494.2107	350.4	POS	1.290	3.37E-10	0.193
50	LPG(14:0)	$C_{20}H_{41}O_9P$	456.2492	816.4	NEG	1.290	3.37E-10	0.193
51	Kynurenic acid	$C_{10}H_7NO_3$	189.0424	453.6	POS	1.290	4.47E-10	0.301
52	(S)-3-Hydroxybutyric acid	$C_4H_8O_3$	104.0471	141.8	POS	1.290	1.42E-09	0.190
53	N-[2-(1H-Indol-3-yl)ethyl]propanamide	$C_{13}H_{16}N_2O$	216.1260	568.9	POS	1.290	7.78E-10	5.225
54	PE O-8:0_6:0	$C_{19}H_{40}NO_7P$	425.2539	783.7	POS	1.290	1.66E-09	0.093
55	Tetrahydrofolic acid	$C_{19}H_{23}N_7O_6$	462.1965	460.7	POS	1.290	5.51E-10	0.192
56	3-Formylindole	C_9H_7NO	145.0525	510.8	POS	1.290	1.31E-09	0.104
57	L-Proline	$C_5H_9NO_2$	115.0631	68.1	POS	1.290	1.78E-09	5.108
58	3-Hydroxybutyric acid	$C_4H_8O_3$	104.0476	187.5	NEG	1.290	2.62E-09	0.061
59	2-(Formylamino)benzoic acid	$C_8H_7NO_3$	165.0424	479.0	POS	1.290	2.51E-09	0.089
60	1,11-Undecanedicarboxylic acid	$C_{13}H_{24}O_4$	244.1679	695.3	NEG	1.290	1.20E-09	0.308
61	N-acetyl-2-phenylethylamine	$C_{10}H_{13}NO$	163.0995	514.2	POS	1.290	1.47E-09	10.214
62	N-Leucyl-leucine	$C_{12}H_{24}N_2O_3$	244.1783	388.9	POS	1.290	1.99E-09	2.687
63	(2R)-3-Hydroxyisovaleroylcarnitine	$C_{12}H_{23}NO_5$	261.1570	282.2	POS	1.290	1.37E-09	0.191
64	Lactobionic Acid	$C_{12}H_{22}O_{12}$	358.1117	73.5	NEG	1.290	2.22E-09	0.138
65	N-Acetylleucine	$C_8H_{15}NO_3$	173.1051	434.2	POS	1.290	3.18E-09	2.654
66	3'-AMP	$C_{10}H_{14}N_5O_7P$	347.0637	266.7	NEG	1.290	4.27E-09	0.188
67	p-Octopamine	$C_8H_{11}NO_2$	170.1052	449.6	POS	1.290	4.60E-09	0.188
68	Umbelliferone	$C_9H_6O_3$	179.0581	495.4	POS	1.290	2.92E-09	0.195
69	N-Alpha-acetyllysine	$C_8H_{16}N_2O_3$	188.1164	69.9	NEG	1.290	2.12E-09	0.178
70	Phosphorylcholine	$C_5H_{15}NO_4P$	201.1000	348.5	POS	1.290	3.00E-09	2.440
71	1*H*-Inden-2-amine, 2,3-dihydro-5-methoxy-6-methyl-	$C_{11}H_{15}NO$	177.1152	562.7	POS	1.289	3.45E-09	10.457
72	Val-Lys	$C_{11}H_{23}N_3O_3$	245.1736	112.2	POS	1.289	4.11E-09	5.304
73	Cyclo(Leu-Pro)	$C_{11}H_{18}N_2O_2$	210.1366	391.3	POS	1.289	5.63E-09	0.076
74	PyroGlu-Ile-Arg	$C_{17}H_{30}N_6O_5$	398.2272	341.8	POS	1.289	2.69E-09	0.189
75	p-Acetaminobenzoic acid	$C_9H_9NO_3$	179.0586	496.4	NEG	1.289	7.15E-09	0.209
76	Oxypurinol	$C_5H_4N_4O_2$	152.0337	145.5	NEG	1.289	7.88E-09	0.187
77	D-Cysteine	$C_3H_7NO_2S$	167.0247	70.4	NEG	1.289	3.16E-09	0.189
78	3-Methoxytyramine	$C_9H_{13}NO_2$	167.0945	288.0	POS	1.289	3.23E-09	0.189
79	*N*-Acetyl-tyrosine	$C_{11}H_{13}NO_4$	223.0848	422.3	NEG	1.289	3.91E-09	0.093

80	p-Aminobenzoic acid	$C_7H_7NO_2$	137.0475	459.3	POS	1.289	5.75E-09	0.232
81	(2E,4Z)-9-Oxooctadeca-2,4-dienoic acid	$C_{18}H_{30}O_3$	294.2197	820.4	NEG	1.289	8.76E-09	0.187
82	PC(18:1(9Z)/18:1(9Z))	$C_{44}H_{84}NO_8P$	807.5752	1003.3	POS	1.289	1.02E-08	0.187
83	Xanthosine	$C_{10}H_{12}N_4O_6$	284.0761	292.4	NEG	1.289	9.59E-09	8.455
84	Valylvaline	$C_{10}H_{20}N_2O_3$	216.1476	294.9	POS	1.289	6.78E-09	6.226
85	Ala-Glu	$C_8H_{14}N_2O_5$	218.0901	121.7	POS	1.289	1.00E-08	8.526
86	Glu-Phe	$C_{14}H_{18}N_2O_5$	294.1220	386.2	NEG	1.289	1.05E-08	9.154
87	Pectin	$C_6H_{10}O_7$	230.0195	75.8	NEG	1.289	9.07E-09	4.015
88	2-(3-Carboxypropionylamino)-4-methylsulfanylbutyric acid	$C_9H_{15}NO_5S$	249.0674	379.3	NEG	1.289	7.64E-09	0.083
89	Methyl myristoleate	$C_{15}H_{28}O_2$	240.2087	838.9	POS	1.289	8.22E-09	6.698
90	Cyclohexanone	$C_6H_{10}O$	98.0730	1244.7	POS	1.289	1.47E-08	0.186
91	Trehalose	$C_{12}H_{22}O_{11}$	364.0978	80.7	POS	1.289	6.43E-09	0.188
92	LPE(15:0)	$C_{20}H_{42}NO_7P$	439.2703	829.2	NEG	1.289	1.09E-08	0.226
93	Acetone	C_3H_6O	99.0683	316.2	POS	1.289	7.86E-09	0.044
94	Tryptophanol	$C_{10}H_{11}NO$	161.0838	526.8	POS	1.289	7.99E-09	80.993
95	*N*-Acetylhistidine	$C_8H_{11}N_3O_3$	197.0797	70.0	NEG	1.289	8.82E-09	2.116
96	gamma-Glutamylleucine	$C_{11}H_{20}N_2O_5$	260.1370	318.3	POS	1.289	8.78E-09	0.120
97	Ribose	$C_5H_{10}O_5$	150.0532	1778.0	POS	1.289	8.88E-09	0.187
98	3,4-Dihydroxymandelic acid	$C_8H_8O_5$	201.0636	378.6	POS	1.289	1.06E-08	0.127
99	Val-Pro	$C_{10}H_{18}N_2O_3$	214.1315	312.3	POS	1.289	1.40E-08	5.381
100	Lauroyl Lysine	$C_{18}H_{36}N_2O_3$	328.2721	670.3	POS	1.289	2.13E-08	0.185

Table S4. Pathway enrichment analysis of metabolites in *Bacillus licheniformis*, highlighting the matched pathways, key metabolites, and statistical significance for each metabolic pathway.

No.	Pathway Name	Match Status	P-value	-log(p)	Holm p	FDR	Impact	Metabolites
1	Glycine, serine and threonine metabolism	7/32	0.0030	2.5177	0.2399	0.2399	0.2647	L-Aspartate; L-Threonine; L-Serine; Tetrahydrofolate; Choline; Betaine; L-Tryptophan
2	D-Amino acid metabolism	6/29	0.0082	2.0867	0.6388	0.2962	0.1778	L-Glutamate; L-Serine; L-Lysine; D-Lysine; D-Arginine; N-Acetyl-L-glutamate
3	Cyanoamino acid metabolism	3/8	0.0112	1.9490	0.8660	0.2962	0.0000	L-Asparagine; L-Aspartate; L-Serine
4	Histidine metabolism	4/18	0.0240	1.6199	1.0000	0.3625	0.1539	Urocanate; L-Histidine; L-Glutamate; Imidazole-4-acetate
5	Arginine biosynthesis	4/18	0.0240	1.6199	1.0000	0.3625	0.2728	L-Glutamate; N-Acetyl-L-glutamate; L-Aspartate; L-Citrulline

6	Methane metabolism	5/29	0.0334	1.4760	1.0000	0.3625	0.1241	Acetate; L-Serine; (S)-Malate; Acetyl phosphate; Tetrahydrofolate
7	Vitamin B6 metabolism	3/12	0.0367	1.4353	1.0000	0.3625	0.0000	D-Ribose 5-phosphate; Pyridoxine; Pyridoxamine
8	Taurine and hypotaurine metabolism	3/12	0.0367	1.4353	1.0000	0.3625	0.1429	Acetate; L-Glutamate; Acetyl phosphate
9	Alanine, aspartate and glutamate metabolism	4/22	0.0473	1.3254	1.0000	0.4149	0.5299	L-Aspartate; L-Asparagine; Succinate semialdehyde; L-Glutamate
10	Glyoxylate and dicarboxylate metabolism	5/34	0.0611	1.2140	1.0000	0.4659	0.1818	Citrate; (S)-Malate; L-Glutamate; Acetate; L-Serine
11	Lysine degradation	3/15	0.0662	1.1795	1.0000	0.4659	0.4444	L-Lysine; D-Lysine; 5-Aminopentanoate
12	Pyruvate metabolism	4/25	0.0708	1.1502	1.0000	0.4659	0.3333	Methylglyoxal; (S)-Malate; Acetate; Acetyl phosphate
13	beta-Alanine metabolism	2/8	0.0886	1.0524	1.0000	0.5386	0.0000	L-Aspartate; Pantothenate
14	Glutathione metabolism	3/18	0.1035	0.9850	1.0000	0.5841	0.1716	Pidolic acid; L-Glutamate; Spermidine
15	Cysteine and methionine metabolism	5/44	0.1460	0.8357	1.0000	0.7522	0.1550	5'-Methylthioadenosine; L-Methionine S-oxide; L-Methionine; L-Serine; L-Aspartate
16	Valine, leucine and isoleucine biosynthesis	3/22	0.1632	0.7873	1.0000	0.7522	0.0000	L-Threonine; 4-Methyl-2-oxopentanoate; L-Valine
17	Pantothenate and CoA biosynthesis	3/22	0.1632	0.7873	1.0000	0.7522	0.1395	Pantothenate; L-Valine; L-Aspartate
18	Tryptophan metabolism	2/12	0.1774	0.7511	1.0000	0.7522	0.0000	Indole-3-acetate; 5-Hydroxyindoleacetate
19	Carbapenem biosynthesis	1/3	0.1809	0.7426	1.0000	0.7522	0.0000	L-Glutamate
20	Arginine and proline metabolism	3/24	0.1962	0.7072	1.0000	0.7603	0.0948	L-Glutamate; L-Proline; Spermidine
21	Pentose phosphate pathway	3/26	0.2308	0.6368	1.0000	0.7603	0.1836	D-Ribose 5-phosphate; D-Gluconic acid; D-Glucono-1,5-lactone
22	Biosynthesis of various plant secondary metabolites	1/4	0.2337	0.6313	1.0000	0.7603	0.0000	L-Methionine
23	Starch and sucrose metabolism	3/27	0.2485	0.6047	1.0000	0.7603	0.1531	D-Fructose; Maltose; Cellobiose
24	Lysine biosynthesis	2/15	0.2502	0.6017	1.0000	0.7603	0.0000	L-Aspartate; L-Lysine
25	Nicotinate and nicotinamide metabolism	2/15	0.2502	0.6017	1.0000	0.7603	0.0000	L-Aspartate; Succinate semialdehyde
26	Sulfur metabolism	2/15	0.2502	0.6017	1.0000	0.7603	0.0489	L-Serine; Acetate
27	Phenylalanine metabolism	1/5	0.2832	0.5479	1.0000	0.7990	0.0000	L-Phenylalanine
28	Biosynthesis of siderophore group nonribosomal peptides	1/5	0.2832	0.5479	1.0000	0.7990	0.3000	2,3-Dihydroxybenzoate
29	Xylene degradation	1/6	0.3295	0.4822	1.0000	0.8976	0.0000	4-Methylcatechol
30	Citrate cycle (TCA cycle)	2/20	0.3723	0.4292	1.0000	0.9172	0.1308	(S)-Malate; Citrate
31	Nitrogen metabolism	1/7	0.3729	0.4285	1.0000	0.9172	0.0000	L-Glutamate
32	Monobactam biosynthesis	1/8	0.4135	0.3836	1.0000	0.9172	0.0000	L-Aspartate
33	Aminobenzoate degradation	1/8	0.4135	0.3836	1.0000	0.9172	0.0000	Benzoate
34	One carbon pool by folate	1/8	0.4135	0.3836	1.0000	0.9172	0.5175	Tetrahydrofolate
35	Butanoate metabolism	2/22	0.4192	0.3776	1.0000	0.9172	0.0171	(R)-3-Hydroxybutanoate; Succinate semialdehyde

36	Phenylalanine, tyrosine and tryptophan biosynthesis	2/22	0.4192	0.3776	1.0000	0.9172	0.0199	L-Tryptophan; L-Phenylalanine
37	Pyrimidine metabolism	3/37	0.4296	0.3670	1.0000	0.9172	0.1664	Uracil; Uridine; Thymidine;
38	Tyrosine metabolism	1/9	0.4515	0.3454	1.0000	0.9386	0.0000	Succinate semialdehyde
39	Propanoate metabolism	2/27	0.5282	0.2772	1.0000	1.0000	0.0075	Propanoate; Methylglyoxal
40	Galactose metabolism	2/27	0.5282	0.2772	1.0000	1.0000	0.0428	Raffinose; D-Fructose
41	Porphyrin metabolism	2/28	0.5484	0.2609	1.0000	1.0000	0.1332	Porphobilinogen; 5-Aminolevulinate
42	Glycolysis / Gluconeogenesis	2/30	0.5869	0.2315	1.0000	1.0000	0.0216	Salicin; Acetate
43	Valine, leucine and isoleucine degradation	2/32	0.6230	0.2056	1.0000	1.0000	0.0352	L-Valine; 4-Methyl-2-oxopentanoate
44	Amino sugar and nucleotide sugar metabolism	2/33	0.6401	0.1938	1.0000	1.0000	0.0000	D-Glucosamine; D-Fructose
45	Riboflavin metabolism	1/16	0.6576	0.1821	1.0000	1.0000	0.1327	Riboflavin
46	Purine metabolism	4/72	0.6976	0.1564	1.0000	1.0000	0.0613	Allantoate; D-Ribose 5-phosphate; Xanthosine; Hypoxanthine
47	Fructose and mannose metabolism	1/20	0.7388	0.1315	1.0000	1.0000	0.2060	D-Fructose
48	Biotin metabolism	1/21	0.7559	0.1215	1.0000	1.0000	0.0792	Biotin
49	Glycerophospholipid metabolism	1/24	0.8010	0.0964	1.0000	1.0000	0.0000	Choline
50	Lipoic acid metabolism	1/28	0.8486	0.0713	1.0000	1.0000	0.0247	Tetrahydrofolate
51	Pentose and glucuronate interconversions	1/29	0.8586	0.0662	1.0000	1.0000	0.0000	D-Ribulose
52	Folate biosynthesis	1/34	0.8997	0.0459	1.0000	1.0000	0.0122	Tetrahydrofolate

Table S5. Pathway enrichment analysis of metabolites in *Priestia megaterium,* highlighting the matched pathways, key metabolites, and statistical significance for each metabolic pathway.

No.	Pathway Name	Match Status	P-value	-log(p)	Holm p	FDR	Impact	Metabolites
1	Arginine biosynthesis	7/18	0.0003	3.5113	0.0243	0.0243	0.4971	L-Arginine; L-Glutamate; N-Acetyl-L-glutamate; L-Aspartate; L-Citrulline; L-Ornithine; 2-Oxoglutarate
2	Tyrosine metabolism	4/9	0.0040	2.4016	0.3094	0.1567	0.0000	3,4-Dihydroxyphenylethyleneglycol; 3-Hydroxyphenylacetate; Succinate semialdehyde; Succinate
3	Valine, leucine and isoleucine biosynthesis	6/22	0.0067	2.1711	0.5192	0.1776	0.0787	L-Threonine; (S)-3-Methyl-2-oxopentanoic acid; L-Leucine; alpha-Isopropylmalate; 4-Methyl-2-oxopentanoate; L-Valine
4	Taurine and hypotaurine metabolism	4/12	0.0128	1.8912	0.9765	0.2066	0.1429	L-Threonine; (S)-3-Methyl-2-oxopentanoic acid; L-Leucine; alpha-Isopropylmalate; 4-Methyl-2-oxopentanoate; L-Valine
5	Pyruvate metabolism	6/25	0.0131	1.8835	0.9809	0.2066	0.3333	Acetate; L-Glutamate; Acetyl phosphate; 2-Oxoglutarate

6	D-Amino acid metabolism	6/29	0.0268	1.5714	1.0000	0.2888	0.0889	L-Glutamate; L-Lysine; L-Arginine; L-Ornithine; N-Acetyl-L-glutamate; 5-Guanidino-2-oxopentanoate
7	Purine metabolism	11/72	0.0284	1.5462	1.0000	0.2888	0.2645	AMP; IMP, Xanthosine; Adenosine; Adenine; GMP; Hypoxanthine; Inosine; Urate; dGMP; 3'-AMP
8	Alanine, aspartate and glutamate metabolism	5/22	0.0292	1.5339	1.0000	0.2888	0.6343	L-Aspartate; Succinate semialdehyde; 2-Oxoglutarate; L-Glutamate; Succinate
9	Arginine and proline metabolism	5/24	0.0414	1.3828	1.0000	0.3309	0.5024	L-Arginine; Agmatine; L-Glutamate; L-Proline; L-Ornithine
10	Glycine, serine and threonine metabolism	6/32	0.0419	1.3779	1.0000	0.3309	0.1985	L-Aspartate; O-Phospho-L-serine; L-Threonine; Tetrahydrofolate; Choline; Betaine
11	Citrate cycle (TCA cycle)	4/20	0.0757	1.1209	1.0000	0.5437	0.1637	2-Oxoglutarate, Succinate; Citrate; Phosphoenolpyruvate
12	Methane metabolism	5/29	0.0836	1.0777	1.0000	0.5505	0.2224	O-Phospho-L-serine; Acetate; Phosphoenolpyruvate; Acetyl phosphate; Tetrahydrofolate
13	Nicotinate and nicotinamide metabolism	3/15	0.1202	0.9202	1.0000	0.6781	0.0000	L-Aspartate; Succinate semialdehyde; Succinate
14	Sulfur metabolism	3/15	0.1202	0.9202	1.0000	0.6781	0.3126	Sulfite; Acetate, Succinate
15	beta-Alanine metabolism	2/8	0.1364	0.8651	1.0000	0.7185	0.0000	L-Aspartate; Pantothenate
16	Histidine metabolism	3/18	0.1808	0.7428	1.0000	0.8547	0.1539	Urocanate; L-Histidine; L-Glutamate
17	Pyrimidine metabolism	5/37	0.1839	0.7354	1.0000	0.8547	0.2843	UMP; Uracil; Uridine; CMP; Thymidine
18	Carbapenem biosynthesis	1/3	0.2282	0.6416	1.0000	0.9736	0.0000	L-Glutamate
19	Vitamin B6 metabolism	2/12	0.2607	0.5839	1.0000	0.9736	0.0000	Pyridoxine; Pyridoxamine
20	Valine, leucine and isoleucine degradation	4/32	0.2685	0.5710	1.0000	0.9736	0.0703	(S)-3-Methyl-2-oxopentanoic acid; L-Leucine; L-Valine; 4-Methyl-2-oxopentanoate
21	Butanoate metabolism	3/22	0.2711	0.5668	1.0000	0.9736	0.0342	(R)-3-Hydroxybutanoate; Succinate semialdehyde; Succinate
22	Pantothenate and CoA biosynthesis	3/22	0.2711	0.5668	1.0000	0.9736	0.1395	Pantothenate; L-Valine; L-Aspartate
23	Biosynthesis of various plant secondary metabolites	1/4	0.2922	0.5343	1.0000	0.9911	0.0000	L-Methionine
24	Glyoxylate and dicarboxylate metabolism	4/34	0.3072	0.5126	1.0000	0.9911	0.1818	Oxalate; Citrate; L-Glutamate; Acetate
25	Phenylalanine metabolism	1/5	0.3510	0.4547	1.0000	0.9911	0.0000	L-Phenylalanine
26	Biosynthesis of siderophore group nonribosomal peptides	1/5	0.3510	0.4547	1.0000	0.9911	0.3000	2,3-Dihydroxybenzoate
27	Lysine biosynthesis	2/15	0.3558	0.4488	1.0000	0.9911	0.0000	L-Aspartate; L-Lysine
28	Lysine degradation	2/15	0.3558	0.4488	1.0000	0.9911	0.2222	L-Lysine; Glutarate
29	Riboflavin metabolism	2/16	0.3867	0.4127	1.0000	0.9911	0.2725	Riboflavin; FMN
30	Propanoate metabolism	3/27	0.3889	0.4102	1.0000	0.9911	0.0126	Propanoate; Succinate; Methylglyoxal

31	Starch and sucrose metabolism	3/27	0.3889	0.4102	1.0000	0.9911	0.0895	D-Fructose; Maltose; Isomaltose
32	Xylene degradation	1/6	0.4049	0.3927	1.0000	0.9932	0.0000	4-Methylcatechol
33	Glutathione metabolism	2/18	0.4464	0.3502	1.0000	0.9932	0.1716	Pidolic acid; L-Glutamate
34	Nitrogen metabolism	1/7	0.4544	0.3426	1.0000	0.9932	0.0000	L-Glutamate
35	Glycolysis / Gluconeogenesis	3/30	0.4579	0.3392	1.0000	0.9932	0.1188	Acetaldehyde; Phosphoenolpyruvate; Acetate
36	Monobactam biosynthesis	1/8	0.4998	0.3012	1.0000	0.9932	0.0000	L-Aspartate
37	Cyanoamino acid metabolism	1/8	0.4998	0.3012	1.0000	0.9932	0.0000	L-Aspartate
38	One carbon pool by folate	1/8	0.4998	0.3012	1.0000	0.9932	0.5175	Tetrahydrofolate
39	Cysteine and methionine metabolism	4/44	0.5012	0.3000	1.0000	0.9932	0.1673	5'-Methylthioadenosine; L-Methionine; L-Aspartate; O-Phospho-L-serine
40	Fructose and mannose metabolism	2/20	0.5029	0.2985	1.0000	0.9932	0.2060	D-Fructose; Mannitol;
41	Phenylalanine, tyrosine and tryptophan biosynthesis	2/22	0.5556	0.2552	1.0000	1.0000	0.0199	Phosphoenolpyruvate; L-Phenylalanine
42	Ascorbate and aldarate metabolism	1/10	0.5797	0.2368	1.0000	1.0000	0.5000	D-Glucarate
43	Glycerophospholipid metabolism	2/24	0.6043	0.2187	1.0000	1.0000	0.1002	sn-Glycero-3-phosphocholine; Choline
44	Tryptophan metabolism	1/12	0.6470	0.1891	1.0000	1.0000	0.0000	5-Hydroxyindoleacetate
45	Pentose phosphate pathway	2/26	0.6490	0.1878	1.0000	1.0000	0.0376	D-Gluconic acid, D-Glucono-1,5-lactone
46	Galactose metabolism	2/27	0.6698	0.1741	1.0000	1.0000	0.0428	Raffinose; D-Fructose
47	Lipoic acid metabolism	2/28	0.6896	0.1614	1.0000	1.0000	0.0247	2-Oxoglutarate, Tetrahydrofolate
48	Porphyrin metabolism	2/28	0.6896	0.1614	1.0000	1.0000	0.1332	Porphobilinogen; 5-Aminolevulinate
49	Pentose and glucuronate interconversions	2/29	0.7084	0.1497	1.0000	1.0000	0.0000	L-Arabinose; 2-Oxoglutarate
50	Amino sugar and nucleotide sugar metabolism	2/33	0.7746	0.1109	1.0000	1.0000	0.0000	D-Fructose; L-Arabinose
51	Folate biosynthesis	2/34	0.7890	0.1030	1.0000	1.0000	0.0356	Tetrahydrofolate; 4-Aminobenzoate
52	Biotin metabolism	1/21	0.8398	0.0758	1.0000	1.0000	0.0792	Biotin
53	Thiamine metabolism	1/25	0.8875	0.0518	1.0000	1.0000	0.0706	Thiamine

Table S6. Enrichment analysis of main-class chemical structures in *Bacillus licheniformis* cocultures with *Fusarium avenaceum*

No	Metabolite Set	Total	Hits	Expect	P-value	Holm P	FDR
1	Carboxylic acids and derivatives	3740	88	4.2300	1.41E-89	6.71E-87	6.71E-87

2	Organooxygen compounds	3160	37	3.5700	4.33E-26	2.06E-23	1.03E-23
3	Indoles and derivatives	559	10	0.6320	1.24E-09	5.86E-07	1.96E-07
4	Phenols	434	9	0.4910	2.36E-09	1.11E-06	2.80E-07
5	Benzene and substituted derivatives	3050	16	3.4500	5.38E-07	2.54E-04	5.12E-05
6	Organonitrogen compounds	618	8	0.6980	6.59E-07	3.11E-04	5.23E-05
7	Phenylpropanoic acids	78	3	0.0882	1.02E-04	4.79E-02	6.93E-03
8	Pyrimidine nucleosides	87	3	0.0983	1.41E-04	6.61E-02	8.38E-03
9	Organic phosphoric acids and derivatives	93	3	0.1050	1.72E-04	8.03E-02	9.07E-03
10	Keto acids and derivatives	114	3	0.1290	0.0003	0.1460	0.0144
11	Fatty Acyls	4680	15	5.3000	0.0003	0.1550	0.0144
12	Purine nucleosides	121	3	0.1370	0.0004	0.1730	0.0148
13	Lactones	136	3	0.1540	0.0005	0.2430	0.0192
14	Imidazopyrimidines	198	3	0.2240	0.0015	0.7150	0.0525
15	Pteridines and derivatives	100	2	0.1130	0.0059	1.0000	0.1860
16	Diazines	342	3	0.3870	0.0071	1.0000	0.2120
17	Hydroxy acids and derivatives	116	2	0.1310	0.0078	1.0000	0.2180
18	Pyridines and derivatives	418	3	0.4720	0.0122	1.0000	0.3230
19	Biotin and derivatives	14	1	0.0158	0.0157	1.0000	0.3940
20	5'-deoxyribonucleosides	27	1	0.0305	0.0301	1.0000	0.7070
21	Non-metal oxoanionic compounds	28	1	0.0316	0.0312	1.0000	0.7070
22	Oxanes	35	1	0.0396	0.0388	1.0000	0.8010
23	Tropane alkaloids	36	1	0.0407	0.0399	1.0000	0.8010
24	Oxepanes	37	1	0.0418	0.0410	1.0000	0.8010
25	Carboximidic acids and derivatives	38	1	0.0429	0.0421	1.0000	0.8010
26	Cinnamic acids and derivatives	300	2	0.3390	0.0458	1.0000	0.8380
27	Coumarins and derivatives	341	2	0.3850	0.0575	1.0000	0.9890
28	Organic phosphonic acids and derivatives	53	1	0.0599	0.0582	1.0000	0.9890
29	Azoles	462	2	0.5220	0.0967	1.0000	1.0000
30	Linear 1,3-diarylpropanoids	106	1	0.1200	0.1130	1.0000	1.0000
31	Organic sulfuric acids and derivatives	122	1	0.1380	0.1290	1.0000	1.0000
32	Naphthalenes	334	1	0.3770	0.3150	1.0000	1.0000
33	Prenol lipids	3830	4	4.3200	0.6300	1.0000	1.0000

Table S7. Enrichment analysis of main-class chemical structures in *Priestia megaterium* cocultures with *Fusarium avenaceum*

**No.**	**Metabolite Set**	**Total**	**Hits**	**Expect**	**P value**	**Holm P**	**FDR**
1	Carboxylic acids and derivatives	3740	88	4.5700	4.78E-86	2.27E-83	2.27E-83
2	Organooxygen compounds	3160	27	3.8500	4.73E-15	2.25E-12	1.13E-12

3	Keto acids and derivatives	114	8	0.1390	2.18E-12	1.04E-09	3.47E-10
4	Phenols	434	10	0.5300	2.32E-10	1.10E-07	2.76E-08
5	Benzene and substituted derivatives	3050	21	3.7300	3.03E-10	1.43E-07	2.88E-08
6	Imidazopyrimidines	198	7	0.2420	6.51E-09	3.07E-06	5.17E-07
7	Purine nucleosides	121	5	0.1480	4.63E-07	2.18E-04	3.15E-05
8	Indoles and derivatives	559	8	0.6830	5.60E-07	2.62E-04	3.33E-05
9	Purine nucleotides	134	5	0.1640	7.68E-07	3.59E-04	4.06E-05
10	Fatty Acyls	4680	20	5.7200	1.72E-06	8.01E-04	8.17E-05
11	Organic phosphoric acids and derivatives	93	4	0.1140	5.83E-06	2.72E-03	2.52E-04
12	Organonitrogen compounds	618	6	0.7550	1.27E-04	5.90E-02	5.03E-03
13	Hydroxy acids and derivatives	116	3	0.1420	0.0004	0.1910	0.0151
14	Non-metal oxoanionic compounds	28	2	0.0342	0.0006	0.2550	0.0187
15	Diazines	342	4	0.4180	0.0009	0.4070	0.0280
16	Pyrimidine nucleotides	77	2	0.0941	0.0041	1.0000	0.1180
17	Phenylpropanoic acids	78	2	0.0953	0.0042	1.0000	0.1180
18	Pyrimidine nucleosides	87	2	0.1060	0.0052	1.0000	0.1370
19	Flavin nucleotides	6	1	0.0073	0.0073	1.0000	0.1830
20	Organic sulfuric acids and derivatives	122	2	0.1490	0.0100	1.0000	0.2370
21	Ribonucleoside 3'-phosphates	9	1	0.0110	0.0109	1.0000	0.2480
22	Lactones	136	2	0.1660	0.0123	1.0000	0.2650
23	Biotin and derivatives	14	1	0.0171	0.0170	1.0000	0.3510
24	Cinnamaldehydes	18	1	0.0220	0.0218	1.0000	0.4320
25	5'-deoxyribonucleosides	27	1	0.0330	0.0325	1.0000	0.6180
26	Furans	30	1	0.0366	0.0360	1.0000	0.6590
27	Oxanes	35	1	0.0428	0.0419	1.0000	0.7380
28	Oxepanes	37	1	0.0452	0.0442	1.0000	0.7520
29	Organic phosphonic acids and derivatives	53	1	0.0647	0.0627	1.0000	1.0000
30	Pyridines and derivatives	418	2	0.5110	0.0932	1.0000	1.0000
31	Pteridines and derivatives	100	1	0.1220	0.1150	1.0000	1.0000
32	Tetrapyrroles and derivatives	103	1	0.1260	0.1180	1.0000	1.0000
33	Linear 1,3-diarylpropanoids	106	1	0.1290	0.1220	1.0000	1.0000
34	Cinnamic acids and derivatives	300	1	0.3660	0.3070	1.0000	1.0000
35	Coumarins and derivatives	341	1	0.4170	0.3410	1.0000	1.0000
36	Quinolines and derivatives	416	1	0.5080	0.3990	1.0000	1.0000
37	Azoles	462	1	0.5640	0.4320	1.0000	1.0000
38	Prenol lipids	3830	5	4.6700	0.5020	1.0000	1.0000
39	Flavonoids	1800	1	2.2000	0.8900	1.0000	1.0000
40	Steroids and steroid derivatives	2040	1	2.4900	0.9180	1.0000	1.0000
41	Glycerophospholipids	40000	1	48.9000	1.0000	1.0000	1.0000

Table S8. LC-MS data concerning LARAPPI/CI-MSI 2D and LARAPPI/CI-MSI 3D ion images

Name	Molecular formula	Ion polarity	Formula of detected ions	RT [s]	$m/z_{meas.}$[a]	$M_{meas.}$[b]	$\Delta m/z$ [ppm]	ΔRT [s]	MS/MS score
Acetone	C_3H_6O	NEG	$[M+HCOO]^-$	13.62	103.04026	58.04205	1.827	-	-
Acetic acid	$C_2H_4O_2$	POS	$[M+H+CH_3CN]^+$	73.5	102.05477	60.02094	-1.816	-	-
2,3-Butanediol	$C_4H_{10}O_2$	POS	$[M+H+CH_3CN]^+$	404.33	132.10178	90.06796	-0.924	-	-
Uracil	$C_4H_4N_2O_2$	POS	$[M+H]^+$	123.99	113.03441	112.02713	-1.028		-
3-Hydroxybutyric acid	$C_4H_8O_3$	NEG	$[M-H]^-$	147.78	103.04027	104.04754	1.924	-10.08	-
Pentadecanoic acid	$C_{15}H_{30}O_2$	POS	$[M+H-H_2O]^+$, $[M+H]^+$	941.78	225.22113	242.22422	-0.716	-	-
Linoleic acid	$C_{18}H_{32}O_2$	NEG	[M-H]-	826.56	265.14840	266.1557	1.853		947.6
Methylsuccinic acid	$C_5H_8O_4$	POS	$[M+Na+CH_3CN]^+$	140.73	196.0580	132.0422	-0.301		
Propionic acid	$C_3H_6O_2$	POS	$[M+H+CH_3CN]^+$	311.91	116.07045	74.03662	-1.362	-0.27	-
Fumaric acid	$C_4H_4O_4$	NEG	$[M-H]^-$	71.32	115.0038	116.0111	1.168	-	630.6
Succinic acid	$C_4H_6O_4$	POS	$[M+Na]^+$	135.41	141.01584	118.02662	0.395	-2.59	-
L-Aspartic acid	$C_4H_7NO_4$	POS	[M+Na]+	64.43	156.0265	133.0373	-1.58	1.25	-
Malic acid	$C_4H_6O_5$	NEG	[M-H]-	78.82	133.0145	134.0217	1.526	0.64	-
p-Aminobenzoic acid	$C_7H_7NO_2$	POS	$[M+H-H_2O]^+$, $[M+H]^+$	459.07	120.04412	137.04743	-2.216	-	997.7
3-Formylindole	C_9H_7NO	POS	$[M+H]^+$	511.07	146.05977	145.0525	-1.82	-	985.6
3-Hydroxyphenylacetic acid	$C_8H_8O_3$	NEG	$[M-H]^-$	402.99	151.0404	152.0477	0.042	-7.59	-

Indole-3-carboxylic acid	$C_9H_7NO_2$	NEG	[M+OH]-	495.77	178.0514	161.0481	2.13	9.47	-
2-Phenylbutyric acid	$C_{10}H_{12}O_2$	POS	$[M+H]^+$	682.14	165.09067	164.08339	-2.043	-	829.3
2-Isopropylmalic acid	$C_7H_{12}O_5$	POS	$[M+Na]^+$	386.28	199.05749	176.06827	-1.04	14.52	-
Hippuric acid	$C_9H_9NO_3$	POS	[M+H]+	384.07	180.0652	179.0579	-1.904	-9.17	
3-Indoleacrylic acid	$C_{11}H_9NO_2$	POS	$[M+H]^+$, $[M+H-H_2O]^+$, $[M+NH_4]^+$, $[M+H-CO_2]^+$	361.56	188.07037	187.06308	-0.775	-	765.9
Diaminopimelic acid	$C_7H_{14}N_2O_4$	POS	$[M+H]^+$, $[M+K]^+$, $[M+Na]^+$	66.3	191.10284	190.09599	1.055	8.82	-
5-Methoxytryptophan	$C_{12}H_{14}N_2O_3$	POS	$[M+H]^+$	366.53	235.10755	234.10028	-0.705	0.71	-
Betaine	$C_5H_{11}NO_2$	POS	[M+K]+	61.61	156.0419	117.0788	-2.045	-1.39	
Pipecolic acid	$C_6H_{11}NO_2$	POS	$[M+NH_4]^+$	73.3	147.11267	129.07885	-0.878	-7.1	-
2-Methylindole	C_9H_9N	POS	$[M+H]^+$	362.82	132.0806	131.0734	-1.098		839.6
2-Hydroxycaproic acid	$C_6H_{12}O_3$	NEG	[M-H]-	434.06	131.0717	132.0789	2.387	0.32	
L-Aspartic acid	$C_4H_7NO_4$	NEG	$[M-H]^-$	65.13	132.0304	133.03767	0.433	1.95	-
Trans-Cinnamic acid	$C_9H_8O_2$	POS	$[M+H]^+$, $[M+H-H_2O]^+$	306.6	149.05952	148.05218	-1.261	-	890.9
4-Ethylbenzoic acid	$C_9H_{10}O_2$	POS	[M+H]+	638.42	151.0748	150.0675	-3.644	0.86	-
N-[2-(1H-Indol-3-yl)ethyl]acetamide	$C_{12}H_{14}N_2O$	POS	$[M+H]^+$, $[M+Na]^+$	526.77	203.11751	202.11026	-1.889	-	958.9

Pentadecanoic acid	$C_{15}H_{30}O_2$	POS	$[M+H-H_2O]^+$, $[M+H]^+$	941.78	225.22113	242.22422	-0.716	-	-
Monolaurin	$C_{15}H_{30}O_4$	POS	$[M+H-H_2O]$+, [M+H]+, [M+Na]+, $[M+Na-CO_2]$+	895.03	257.2109	274.2142	-0.714		825.6
Linolenic acid	$C_{18}H_{30}O_2$	POS	$[M+H]^+$, $[M+H+CH_3CN]^+$, $[M+H-H_2O]^+$, $[M+Na]^+$	1031.21	279.23155	278.22349	-1.509	-	923.8

[a]experimental *m/z* of monoisotopic signal; [b]experimental neutral monoisotopic mass
